# Oncogenic GPRIN1 sustains proliferation and mitochondrial homeostasis via dual‑layer CDK1-PI3K/Akt signalling in gallbladder cancer

**DOI:** 10.1038/s41419-026-08550-2

**Published:** 2026-03-21

**Authors:** Chang Xu, Zijun Gong, Xiaojian Ni, Lingxi Nan, Wentao Sun, Houbao Liu, Xuanming Luo, Min Li

**Affiliations:** 1 Department of Biliary Surgery, Zhongshan Hospital, Fudan University, Shanghai, China; 2 Department of General Surgery, Xuhui Central Hospital, Fudan University, Shanghai, China

**Keywords:** Cancer, Cancer metabolism

## Abstract

Novel therapeutic targets are urgently needed for the aggressive malignancy gallbladder cancer (GBC). G-protein regulated inducer of neurite outgrowth 1 (GPRIN1) is a candidate oncogene, but its function in GBC and its connection to mitochondrial dysregulation remain unknown. In this study, we analyzed clinical samples and demonstrated that GPRIN1 is significantly upregulated in GBC tissues, where its high expression correlates with advanced clinical stage and poor patient prognosis. Functional assays revealed that GPRIN1 is essential for GBC progression, driving cell cycle advancement and maintaining mitochondrial homeostasis. By integrating proteomic and molecular analyses, our study delineates a bimodal and hierarchical regulatory program commanded by GPRIN1 to ensure the robust activation of CDK1. In the nucleus, GPRIN1 functions as a transcriptional co-activator, scaffolding and stabilizing E2F1 to drive CDK1 expression. In parallel, it functions at a post-translational level to directly promote CDK1 activation by physically steering the kinase away from its inhibitor, MYT1, and toward its activator, Cdc25C. This dual-pronged regulation culminates in hyperactivated CDK1, which in turn unleashes a PI3K-Akt signaling cascade to couple relentless cell proliferation with the necessary mitochondrial support. Importantly, genetic or pharmacological disruption of this GPRIN1-CDK1-PI3K/Akt axis completely abrogated tumorigenesis in vitro and in vivo. Taken together, these results reveal GPRIN1 as a master regulator whose dual transcriptional and post-translational control of CDK1 integrates cell cycle progression with mitochondrial homeostasis, suggesting that targeting GPRIN1 may represent a highly specific therapeutic strategy in this lethal malignancy.

## Introduction

Gallbladder cancer (GBC) is the most aggressive malignancy of the biliary tract [1], with a dismal 5-year survival rate of less than 10% due to late diagnosis and high rates of recurrence [2, 3]. The lack of robust molecular stratification has hindered the development of effective targeted therapies, making the identification of core molecular drivers a paramount goal for this lethal disease [4, 5].

Two fundamental processes are recognized as hallmarks of cancer progression: the dysregulation of the cell cycle and the strategic maintenance of mitochondrial homeostasis. Mitochondria are not only cellular powerhouses but also critical signaling hubs that dictate cell fate [6, 7]. To sustain relentless proliferation, cancer cells are profoundly dependent on mitochondrial quality control (MQC) systems [8, 9], such as mitophagy, to manage metabolic stress and evade apoptosis [10]. While targeting MQC is a promising therapeutic strategy, the regulation of these pathways in GBC remains a complete knowledge gap [11–16].

Intriguingly, evidence is mounting for a direct crosstalk between the core cell cycle machinery and mitochondrial homeostasis. Cyclin-dependent kinase 1 (CDK1), the master regulator of the G2/M transition, and the pro-survival PI3K-Akt signaling cascade are both frequently hyperactivated in biliary tract cancers and are prime therapeutic targets [17–20]. However, a common upstream regulator that coordinately orchestrates these two critical axes—cell cycle progression and mitochondrial integrity—has not been identified.

The G-protein regulated inducer of neurite outgrowth 1 (GPRIN1) has emerged as a multifaceted signaling protein implicated in various human cancers [21–23]. Our prior work revealed GPRIN1 as a key pro-tumorigenic factor in GBC [24]. Crucially, despite lacking intrinsic kinase activity, GPRIN1 functions as a phosphoprotein-binding adapter and is itself subject to extensive phosphorylation, placing it at the core of phosphorylation-dependent signaling networks [21, 25–27]. This positions GPRIN1 as a potential regulator of core cellular processes that are themselves tightly controlled by phosphorylation, such as the profound rewiring of mitochondrial pathways [28]. Maintaining mitochondrial homeostasis—including bioenergetics, membrane potential, and mitophagy—is a key dependency and therapeutic vulnerability in cancer [29]. Therefore, a critical unanswered question is whether GPRIN1’s oncogenic program extends to the regulation of these phosphorylation-dependent mitochondrial processes. Answering this is crucial, as it could provide a direct mechanistic rationale for targeting GPRIN1 in GBC.

In this study, we address this gap by revealing a dual-pronged mechanism where GPRIN1 acts as a master regulator of CDK1 at both transcriptional and post-translational levels. We demonstrate that this GPRIN1-CDK1 regulatory core is essential for synchronizing cell cycle progression with mitochondrial homeostasis to drive GBC aggressiveness, thus identifying the GPRIN1-CDK1 interface as a highly specific therapeutic target in this lethal malignancy.

## Materials and methods

### Cell culture and reagents

Human GBC cell lines GBC-SD and NOZ were obtained from the Cell Bank of the Chinese Academy of Sciences (Shanghai, China). The identity of the cell lines was authenticated by STR profiling, and all cultures were routinely tested and found to be free of mycoplasma contamination. Cells were cultured in RPMI-1640 medium (Gibco, Thermo Fisher Scientific, Cat# 11875093) supplemented with 10% fetal bovine serum (FBS; Gibco, Cat# 10099141 C), 100 U/mL penicillin, and 100 µg/mL streptomycin (Gibco, Cat# 15140122) at 37°C in a humidified atmosphere with 5% CO_2_. The PI3K inhibitor Buparlisib (MCE, Cat# HY-70063) was dissolved in DMSO and used at a final concentration of 1 µM. Cycloheximide (CHX; Sigma-Aldrich, Cat# C7698) was used at 50 µg/mL to inhibit protein synthesis.

### Plasmids, shRNA, and transfection

Short hairpin RNAs (shRNAs) targeting human GPRIN1 (shGPRIN1-1, shGPRIN1-2), CDK1 (shCDK1), E2F1 (shE2F1), and a non-targeting shNC were synthesized and cloned into the pLKO.1-puro vector (Addgene plasmid #8453) by Tsingke Biotechnology Co.Ltd (Shanghai, China). Target sequences were listed **in** Supplementary Table 1. The full-length human GPRIN1 cDNA (NM_001135705.2), CDK1 cDNA (NM_001786.5), and E2F1 cDNA (NM_005225.3) were PCR-amplified from human GBC cell cDNA and cloned into the pcDNA3.1(+) vector (Invitrogen, Thermo Fisher Scientific, Cat# V79020) for overexpression (GPRIN1-OE, CDK1-OE, E2F1-OE). GPRIN1 truncation mutants (1-350, 351-868, 869-1008) were generated by PCR and cloned into pEGFP-C1 (Clontech, Takara Bio, Cat# 6084-1) or pGEX-4T-1 (GE Healthcare, Cat# 27-1542-01) vectors. The GPRIN1-MUT (deficient in E2F1 binding, specific mutations in 869-1008 region to be detailed if known, or described as site-directed mutagenesis product) was generated using a QuikChange Site-Directed Mutagenesis Kit (Agilent Technologies, Cat# 200513). All constructs were verified by DNA sequencing.

Transient transfections were performed using Lipofectamine 3000 Reagent (Invitrogen, Thermo Fisher Scientific, Cat# L3000015) according to the manufacturer’s protocol. Stable cell lines were established by transfecting cells with shRNA plasmids followed by selection with puromycin (2 µg/mL; Sigma-Aldrich, Cat# P8833) for 2 weeks.

### RNA extraction and quantitative real-time PCR (qRT-PCR)

Total RNA was extracted using TRIzol Reagent (Invitrogen, Thermo Fisher Scientific, Cat# 15596026CN). cDNA was synthesized using the PrimeScript™ RT Reagent Kit with gDNA Eraser (Takara Bio, Cat# RR047A). qRT-PCR was performed using TB Green® Premix Ex Taq™ II (Tli RNaseH Plus) (Takara Bio, Cat# RR820A) on a QuantStudio 5 Real-Time PCR System (Applied Biosystems, Thermo Fisher Scientific). Relative mRNA expression was calculated using the 2^−ΔΔCt^ method, with GAPDH as the internal control. Primer sequences were listed in Supplementary Table 2.

### Western blotting and antibodies

Cells or tissues were lysed in RIPA buffer (Thermo Fisher Scientific, Cat# 89901) supplemented with protease and phosphatase inhibitor cocktails (Sigma-Aldrich, Cat# P9599 and Cat# P0001). Protein concentrations were determined using the BCA Protein Assay Kit (Thermo Fisher Scientific, Cat# 23225). Equal amounts of protein (20–30 µg) were separated by SDS-PAGE (Bio-Rad, Cat# 1610156, various percentages) and transferred to PVDF membranes (Millipore, Cat# IPVH00010). Membranes were blocked with 5% non-fat milk or 5% BSA in TBST for 1 h at room temperature and then incubated overnight at 4 °C with primary antibodies. After washing with TBST, membranes were incubated with HRP-conjugated secondary antibodies (Goat anti-Rabbit IgG HRP, CST# 7074, 1:3000; Goat anti-Mouse IgG HRP, CST# 7076, 1:3000) for 1 hour at room temperature. Protein bands were visualized using an ECL Western Blotting Substrate (Thermo Fisher Scientific, Cat# 32106) and imaged with a ChemiDoc XRS+ System (Bio-Rad). antibody informations were list in Supplement Table 3.

### Co-immunoprecipitation (Co-IP) and GST pulldown

For Co-IP, cells were lysed in IP lysis buffer (20 mM Tris-HCl pH 7.5, 150 mM NaCl, 1 mM EDTA, 1% Triton X-100, supplemented with protease and phosphatase inhibitors). Lysates (500 µg-1mg) were incubated with 2–4 µg of primary antibody or control IgG (CST, Cat# 3900 for rabbit, Cat# 5415 for mouse) overnight at 4 °C, followed by incubation with Protein A/G Magnetic Beads (Thermo Fisher Scientific, Cat# 88802) for 2–4 h at 4 °C. Beads were washed three times with IP lysis buffer, and immunoprecipitated proteins were eluted with SDS loading buffer and analyzed by Western blotting.

For GST pulldown, GST-tagged GPRIN1 full-length or fragments expressed in E. coli BL21(DE3) were purified using Glutathione Sepharose 4B beads (GE Healthcare, Cat# 17-0756-01). His-tagged E2F1 protein was expressed and purified or cell lysates containing His-E2F1 were used. GST-fusion proteins bound to beads were incubated with His-E2F1 or cell lysates containing His-E2F1 for 4 h at 4 °C. After washing, bound proteins were analyzed by Western blotting with anti-His antibody. Antibody informations were list in Supplement Table 3.

### Luciferase reporter assay

The human CDK1 promoter region (−2000 to +100 bp relative to TSS) was cloned into the pGL3-Basic vector (Promega, Cat# E1751) to generate CDK1-WT-luc. A mutant CDK1 promoter construct with mutated E2F1 binding sites (CDK1-MUT-luc) was generated by site-directed mutagenesis. Cells were co-transfected with reporter plasmids, a Renilla luciferase internal control plasmid (pRL-TK, Promega, Cat# E2241), and GPRIN1/E2F1 expression vectors or empty vector. After 48 h, luciferase activity was measured using the Dual-Luciferase Reporter Assay System (Promega, Cat# E1910) on a GloMax Navigator Microplate Luminometer (Promega).

### Chromatin immunoprecipitation (ChIP) and sequential ChIP (Re-ChIP)

ChIP assays were performed using the SimpleChIP® Enzymatic Chromatin IP Kit (Magnetic Beads) (Cell Signaling Technology, Cat# 9003) according to the manufacturer’s protocol. Briefly, cells were cross-linked with 1% formaldehyde, lysed, and sonicated to shear chromatin (Bioruptor Pico, Diagenode). Chromatin was immunoprecipitated overnight at 4 °C with antibodies against GPRIN1, E2F1, or normal rabbit IgG. DNA was purified and analyzed by qRT-PCR using primers specific for the CDK1 promoter region containing putative E2F1 binding sites.

For Re-ChIP, after the first IP (GPRIN1), complexes were eluted with 10 mM DTT, diluted, and subjected to a second IP with another antibody (E2F1). Antibody informations were list in Supplement Table 3.

### Cell proliferation assay

Cell proliferation was assessed using the Cell Counting Kit-8 (CCK-8; Dojindo Molecular Technologies, Cat# CK04). Cells (2 × 10^3^ per well) were seeded in 96-well plates. At indicated time points, 10 µL of CCK-8 solution was added to each well and incubated for 2 h at 37 °C. Absorbance at 450 nm was measured using a microplate reader (SpectraMax M5, Molecular Devices).

### Cell cycle analysis

Cells were harvested, washed with PBS, and fixed in 70% ice-cold ethanol overnight at −20 °C. Fixed cells were washed with PBS and incubated with RNase A (100 µg/mL; Thermo Fisher Scientific, Cat# EN0531) and propidium iodide (PI, 50 µg/mL; Sigma-Aldrich, Cat# P4170) for 30 min at 37 °C in the dark. Cell cycle distribution was analyzed using a FACSC alibur flow cytometer (BD Biosciences) and ModFit LT software.

### Mitochondrial function assays

ATP Levels: Cellular ATP levels were measured using the ATP Bioluminescence Assay Kit HS II (Roche, Cat# 11699695001) according to the manufacturer’s instructions. Luminescence was measured on a GloMax luminometer.

Reactive Oxygen Species (ROS) Levels: Cellular ROS levels were measured using the Reactive Oxygen Species (ROS) Assay Kit (Beyotime, Cat# S0033S) according to the manufacturer’s instructions. Following treatment, cells were incubated with a DCFH-DA probe for 20 min at 37 °C. Fluorescence was subsequently measured on a GloMax multi-mode reader with an excitation wavelength of 488 nm and an emission wavelength of 525 nm.

#### Mitochondrial membrane potential (MMP)

MMP was assessed using the JC-1 MMP Assay Kit (Cayman Chemical, Cat# 10009172). Cells were stained with JC-1 dye and analyzed by fluorescence microscopy (Zeiss Axio Observer).

#### MitoTracker red staining

Active mitochondria were stained with MitoTracker Red CMXRos (Invitrogen, Thermo Fisher Scientific, Cat# M7512) at 100 nM for 30 min. Fluorescence intensity was observed using a fluorescence microscope.

### Transmission electron microscopy (TEM)

Cells or tumor tissues were fixed with 2.5% glutaraldehyde in 0.1 M phosphate buffer (pH 7.4) overnight at 4 °C, post-fixed with 1% osmium tetroxide, dehydrated in a graded ethanol series, and embedded in Epon 812 resin (Electron Microscopy Sciences, Cat# 14120). Ultrathin sections (70 nm) were cut using an ultramicrotome (Leica EM UC7), stained with uranyl acetate and lead citrate, and examined under a transmission electron microscope (JEOL JEM-1400).

### Free phosphate assay

Intracellular free phosphate levels were measured using a Phosphate Assay Kit (Abcam, Cat# ab65622) according to the manufacturer’s protocol. Absorbance was read at 650 nm.

### Cycloheximide (CHX) chase assay

Cells were treated with CHX (50 µg/mL) for the indicated time points (0, 4, 6, 8 h). Cell lysates were collected at each time point and subjected to Western blot analysis.

### Nuclear and cytoplasmic extraction

Nuclear and cytoplasmic fractions were isolated using the NE-PER Nuclear and Cytoplasmic Extraction Reagents (Thermo Fisher Scientific, Cat# 78833) according to the manufacturer’s instructions. Lamin B1 (CST, Cat# 13435, 1:1000) and GAPDH were used as nuclear and cytoplasmic markers, respectively.

### Proteomics and phosphoproteomics analysis

GBC-SD cells transfected with shNC or shGPRIN1 were lysed, and proteins were digested with trypsin. Peptides were labeled with Tandem Mass Tags (TMTpro™ 16plex Label Reagent Set, Thermo Fisher Scientific, Cat# A44520). For phosphoproteomics, phosphopeptides were enriched using TiO_2_ beads (GL Sciences). Labeled peptides were fractionated by high-pH reversed-phase liquid chromatography and analyzed by LC-MS/MS on an Orbitrap Fusion Lumos Tribrid Mass Spectrometer (Thermo Fisher Scientific) coupled with an EASY-nLC 1200 system (Thermo Fisher Scientific). Data were analyzed using Proteome Discoverer software (version 2.4, Thermo Fisher Scientific) against a human protein database (UniProt). Differentially expressed proteins/phosphosites were identified based on fold change >1.2 and *p* < 0.05. Pathway enrichment analysis was performed using Metascape or DAVID.

### Immunohistochemistry (IHC)

Paraffin-embedded tumor sections (4 µm) or human GBC tissue microarrays were deparaffinized and rehydrated. Antigen retrieval was performed by boiling in citrate buffer (pH 6.0, Vector Labs, Cat# H-3300) for 15 min. Sections were blocked with 3% H_2_O_2_ and then with 5% BSA. Slides were incubated with primary antibodies (GPRIN1, Ki67, p-Akt, p-PI3K, PARKIN, PINK1, CyclinB1 - dilutions as per WB or optimized for IHC) overnight at 4 °C. After washing, sections were incubated with an HRP-conjugated secondary antibody (EnVision™+ Dual Link System-HRP, Dako, Agilent, Cat# K4065) and visualized with 3,3’-diaminobenzidine (DAB; Dako, Cat# K3468). Representative high-magnification images of individual tumor sections were captured using a light microscope (Olympus BX53). For comprehensive quantitative analysis, the human GBC tissue microarrays were digitized using a whole-slide scanner (KF-SCAN-PL) to create high-resolution digital images. These digital images were subsequently analyzed using the open-source quantitative image analysis software QuPath (version 0.6.0) [30]. The analysis workflow involved automated identification of tumor tissue within each core, followed by individual cell segmentation based on the nuclear hematoxylin counterstain. Each cell was then classified based on the intensity of its DAB signal into four categories: negative (0), weakly positive (1 + ), moderately positive (2 + ), and strongly positive (3 + ). A quantitative H-score was calculated for each core using the formula: H-score = [1 × (% of cells scoring 1 + ) + 2 × (% of cells scoring 2 + ) + 3 × (% of cells scoring 3 + )], resulting in a continuous score from 0 to 300. The automated scoring results and region-of-interest selections were reviewed and confirmed by two independent pathologists who were blinded to the clinical data. For the survival analysis, patients were stratified into GPRIN1-high and GPRIN1-low expression groups using the median H-score of the entire cohort as the cutoff.

### Animal studies (Xenografts)

All animal experiments were approved by the Institutional Animal Care and Use Committee of Zhongshan Hospital and conducted in accordance with institutional guidelines. Female BALB/c nude mice (4–6 weeks old; Vital River Laboratory Animal Technology Co., Beijing, China) were used. For xenograft tumor models, GBC-SD cells (5 × 10^6^ in 100 μL PBS) stably expressing shNC, shGPRIN1, GPRIN1-OE, GPRIN1-OE + shCDK1, or NC were subcutaneously injected into the right flank of mice. Once tumors reached ~100 mm^3^, mice were randomized to treatment groups. The investigators performing tumor measurements and subsequent data analysis were blinded to the group assignments until the study was complete.

For inhibitor studies, mice bearing GPRIN1-OE or NC tumors received Buparlisib (BKM120; 30–35 mg/kg, p.o., once daily) formulated in 10% N-methyl-2-pyrrolidone (NMP) / 90% PEG300 (v/v); vehicle controls received the matching solvent (dose volume 10 mL/kg). Tumor volume was measured every 3–4 days using calipers (Volume = length × width^2 / 2). If body-weight loss exceeded 10%, dosing was modified to a 5-days-on/2-days-off schedule. At the endpoint (4 weeks or when tumors reached ethical limits), mice were euthanized, and tumors were excised, weighed, photographed, and either snap-frozen in liquid nitrogen for biochemical analyses or fixed in 4% paraformaldehyde for IHC.

### Human tissue samples

A cohort of 100 GBC tissues and matched adjacent non-tumorous tissues were obtained from patients who underwent surgical resection at Zhongshan Hospital between 2015 and 2022. This study was approved by the Clinical Research Ethics Committee of Zhongshan Hospital, and written informed consent was obtained from all patients. GPRIN1 expression was assessed by IHC as described above.

### Statistical analysis

Data are presented as mean ± standard deviation (SD) from at least three independent experiments. Assumptions of normality and homogeneity of variance were verified before the application of parametric tests. Statistical analyses were performed using GraphPad Prism 8.0 (GraphPad Software, Inc.). Differences between two groups were analyzed using Student’s *t*-test. Comparisons among multiple groups were performed using one-way ANOVA followed by Tukey’s post hoc test. Kaplan–Meier survival analysis with log-rank test was used to compare overall survival rates. Correlations were analyzed using Pearson’s correlation coefficient. A *p* < 0.05 was considered statistically significant (*p < 0.05, **p < 0.01, ***p < 0.001).

## Results

### GPRIN1 is essential for mitochondrial homeostasis and prevents mitophagy failure in GBC cells

**Fig. 1:**
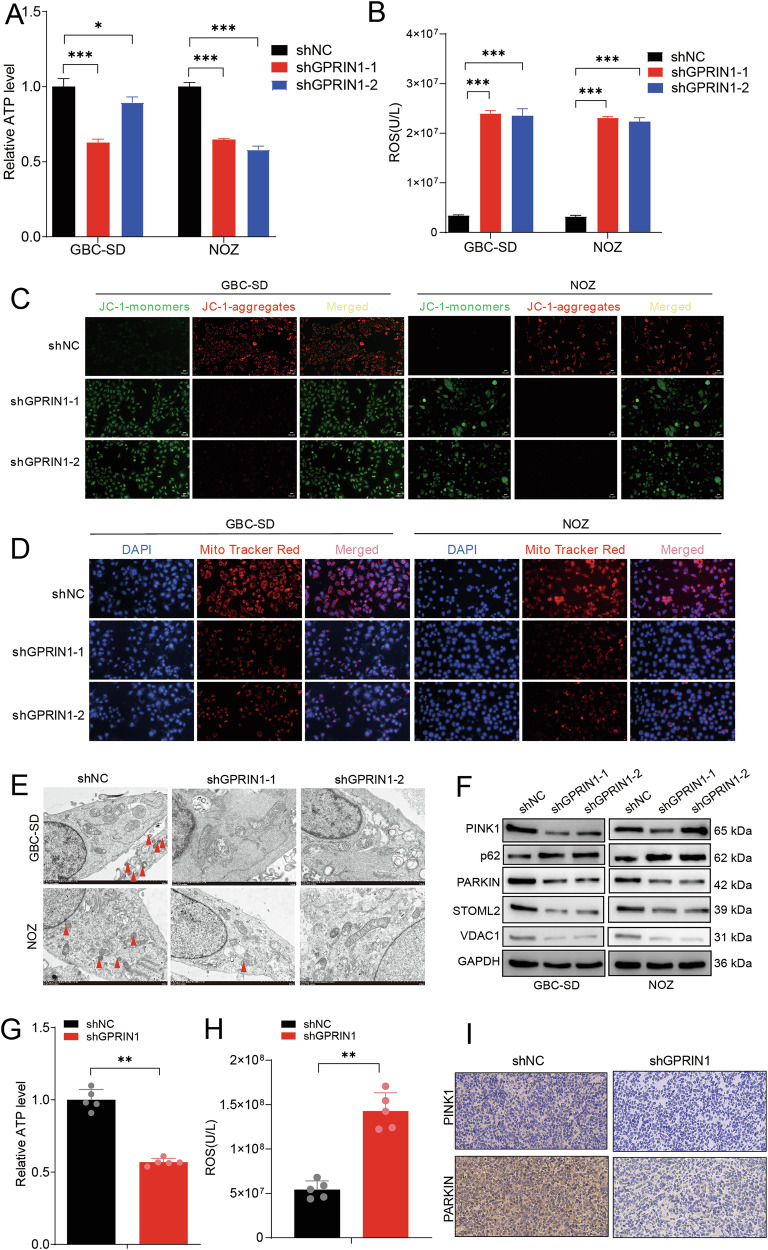
GPRIN1 is crucial for mitochondrial function and regulates mitophagy in GBC cells. Relative ATP levels (**A**) and ROS levels (**B**) in GBC-SD and NOZ cells with GPRIN1 knockdown (shGPRIN1-1, shGPRIN1-2 vs. shNC). **C** JC-1 fluorescence microscopy of GBC-SD and NOZ cells with GPRIN1 knockdown. Green: monomers (low MMP), Red: aggregates (high MMP). Scale bar = 50 µm. **D** MitoTracker Red (active mitochondria) and DAPI (nuclei) staining in GBC-SD and NOZ cells with GPRIN1 knockdown. Scale bar = 50 µm. **E** TEM images of GBC-SD and NOZ cells with GPRIN1 knockdown. Red arrowheads: mitophagosomes. Scale bars indicated. **F** Western blots for mitophagy proteins (PINK1, p62, PARKIN, STOML2, VDAC1) in GBC-SD and NOZ cells with GPRIN1 knockdown. Relative ATP levels (**G**) and ROS levels (**H**) in xenograft tumors from GBC-SD cells with GPRIN1 knockdown or shNC (*n* = 5 mice/group). **I** IHC for PINK1 and PARKIN in xenograft tumor sections from (**G**). Scale bar = 50 µm. *P < 0.05, **P < 0.01, ***P < 0.001.

To dissect how GPRIN1 fuels GBC proliferation [24], we investigated its role in governing mitochondrial homeostasis. We first assessed cellular ATP content following GPRIN1 manipulation in GBC-SD and NOZ cells. Genetic depletion of GPRIN1 using two independent shRNAs (Fig. S1A) led to a significant reduction in intracellular ATP levels compared to control cells (Fig. 1A), indicating compromised energy production. We also found that GPRIN1 depletion induced a profound state of mitochondrial distress. This was evidenced by a dramatic increase in intracellular ROS levels (Fig. 1B) and a concurrent loss of MMP, visualized as a shift from red-fluorescent JC-1 aggregates to green monomers (Fig. 1C). Consistent with this mitochondrial dysfunction, staining with MitoTracker Red, a dye that accumulates in active mitochondria, revealed a markedly diminished mitochondrial mass in GPRIN1-depleted cells (Fig. 1D). To directly visualize the morphological consequences of GPRIN1 loss and investigate potential defects in mitochondrial quality control, we performed TEM with GPRIN1-knockdown cells, which revealed swollen mitochondria with disorganized and fragmented cristae, hallmark features of severe mitochondrial damage. Critically, despite this extensive damage, we observed a decreased incidence of mitophagosomes—identified as double-membrane structures engulfing mitochondria—suggesting an impairment in their clearance (Fig. 1E). To test this hypothesis at the molecular level, we examined the canonical PINK1/PARKIN mitophagy pathway [31]. Intriguingly, GPRIN1 depletion led to a suppression of this pathway, characterized by reduced PINK1 and PARKIN protein levels. This was accompanied by an accumulation of the autophagy receptor p62/SQSTM1 and decreased levels of the mitochondrial proteins STOML2 and VDAC1 (Fig. 1F). Together, these data indicate that GPRIN1 loss causes severe mitochondrial damage but simultaneously is associated with impaired mitophagy machinery required for clearing these dysfunctional organelles. Interestingly, the knockdown of GPRIN1 did not affect the protein levels of key receptors involved in non-canonical mitophagy pathways, suggesting that GPRIN1’s role is specific to the canonical PINK1/PARKIN pathway (Fig. S1H).

Conversely, ectopic GPRIN1 overexpression (Fig. S1B) demonstrated a protective effect on mitochondrial function. GPRIN1 overexpression significantly increased basal ATP levels and reduced ROS production (Fig. S1C, D). To further test the protective capacity of GPRIN1, we challenged the cells with the mitochondrial uncoupler CCCP. As shown by JC-1 and MitoTracker Red staining, GPRIN1 overexpression markedly preserved mitochondrial membrane potential and mitochondrial mass in the face of CCCP-induced stress (Fig. S1E, F). Consistent with this protective role, transmission electron microscopy revealed that while CCCP induced robust mitophagy in control cells, GPRIN1-overexpressing cells displayed a significantly attenuated mitophagic response, indicating a reduced need for mitochondrial clearance due to diminished damage (Fig. S1G).

To determine if these GPRIN1-mediated effects were recapitulated in vivo, we analyzed xenograft tumors derived from GBC-SD cells. Consistent with our in vitro findings, GPRIN1-depleted tumors displayed clear signs of mitochondrial distress, exhibiting significantly lower ATP levels and a marked increase in oxidative stress, as evidenced by elevated ROS (Fig. 1G, H). IHC analysis further substantiated the molecular data, showing markedly weaker staining for PINK1 and PARKIN in shGPRIN1 tumors (Fig. 1I). Taken together, these data establish that GPRIN1 is essential for maintaining mitochondrial health and bioenergetic capacity in GBC. Its depletion triggers mitochondrial damage while crippling the PINK1/PARKIN-mediated mitophagy pathway required for their clearance.

### CDK1 as a key downstream target of GPRIN1 in gallbladder cancer

To identify downstream effectors of GPRIN1, we performed TMT-based global proteomic and phosphoproteomic profiling in GBC cells. GPRIN1 knockdown induced broad alterations in both the total proteome (Fig. S2A) and, more prominently, the phosphoproteome (Fig. S2B). GO enrichment of downregulated phosphosites revealed terms associated with cell-cycle regulation, including mitotic nuclear division (Fig. S2C). Motif-X analysis further identified a strong enrichment of a proline-directed CDK consensus motif (.R….SP…..) (Fig. S2D). Consistently, kinase–substrate network modeling highlighted CDK1 as a central kinase showing coordinated loss of substrate phosphorylation (Fig. S2E).

**Fig. 2:**
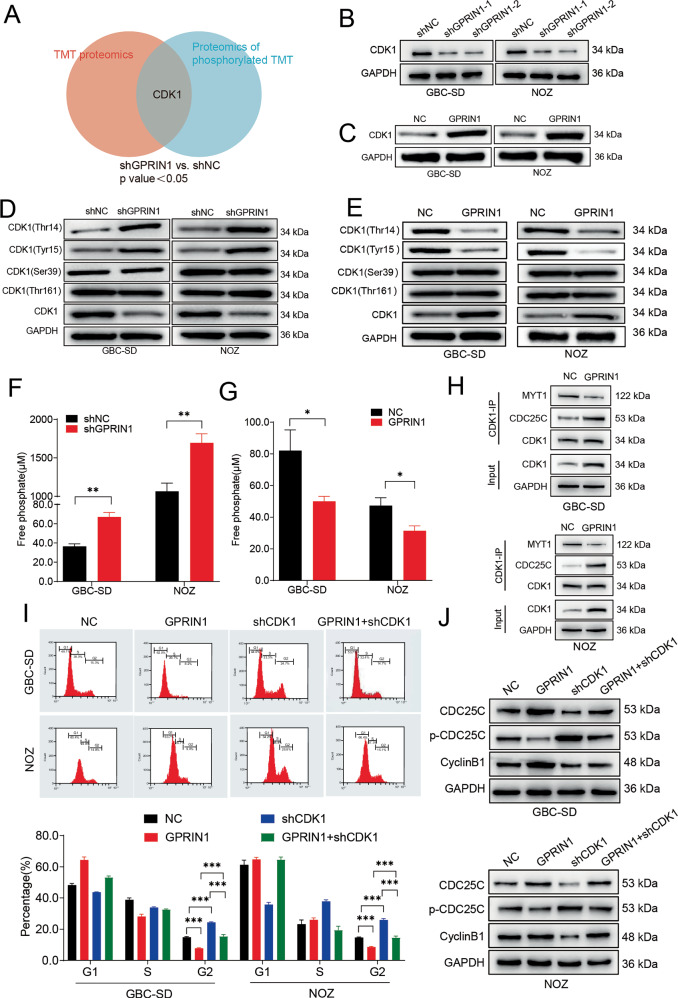
GPRIN1 modulates CDK1 to drive G2/M progression in GBC cells. **A** Venn diagram of proteins altered in TMT proteomics and phosphoproteomics upon GPRIN1 knockdown, highlighting CDK1 (*p* < 0.05). Western blots for total CDK1 in GBC-SD and NOZ cells with (**B**) GPRIN1 knockdown (shGPRIN1-1, shGPRIN1-2 vs. shNC) or (**C**) GPRIN1 overexpression (GPRIN1 vs. NC). Western blots for CDK1 phosphorylation (Thr14, Tyr15, Ser39, Thr161) and total CDK1 in GBC-SD and NOZ cells with (**D**) GPRIN1 knockdown or (**E**) GPRIN1 overexpression. Intracellular free phosphate levels in GBC-SD and NOZ cells with (**F**) GPRIN1 knockdown or (**G**) GPRIN1 overexpression. **H** Co-IP of CDK1 with MYT1 or CDC25C in GBC-SD and NOZ cells overexpressing GPRIN1 or NC. **I** Flow cytometry cell cycle analysis of GBC-SD and NOZ cells: NC, GPRIN1, shCDK1, or GPRIN1+shCDK1. Representative histograms and quantification are shown. **J** Western blots for CDC25C, p-CDC25C(Ser216), and CyclinB1 in GBC-SD and NOZ cells under conditions in (**I**). *P < 0.05, **P < 0.01, ***P < 0.001.

Based on these integrated analyses, we focused on CDK1. The significance of CDK1 as a primary candidate was underscored by its dual-level dysregulation; it was significantly altered at both the total protein level and within the phosphoproteomic network, a finding visually summarized by the Venn diagram (Fig. 2A). Western blot analysis confirmed that GPRIN1 knockdown reduced total CDK1 abundance, whereas GPRIN1 overexpression increased CDK1 protein levels (Fig. 2B, C). Interrogation of the phosphoproteomic dataset further revealed a significant increase in phosphorylation within the N-terminal regulatory region that contains the canonical inhibitory residues Thr14/Tyr15 (log2 Fold Change = 1.38, *P* < 0.001; Supplementary Table 4). Because this tryptic peptide sequence is shared between CDK1 and CDK2, site-specific attribution is not possible in discovery-mode MS; however, the observed increase in phosphorylation within this inhibitory domain is fully consistent with the direction of change identified by our targeted Western blotting. Specifically, phospho-specific Western blotting demonstrated that GPRIN1 depletion increased inhibitory phosphorylation at CDK1-Thr14/Tyr15 and decreased activating phosphorylation at CDK1-Thr161, whereas GPRIN1 overexpression produced the opposite pattern (Fig. 2D, E). Phosphorylation at the non-regulatory Ser39 site remained unchanged in the MS dataset (Supplementary Table 4).

These data reveal GPRIN1 both increases CDK1 levels and promotes its activation. Consistent with a role for GPRIN1 in governing cellular phosphorylation, its depletion led to a marked accumulation of free intracellular phosphate, whereas conversely, its overexpression caused a significant consumption of this pool (Fig. 2F, G), suggesting GPRIN1 tips the cellular kinase-phosphatase balance toward net protein phosphorylation. Since CDK1 activity is tightly balanced by the inhibitory kinase MYT1 and the activating phosphatase Cdc25C [32, 33], we hypothesized that GPRIN1 could modulate their interaction with CDK1. Co-IP assays revealed that GPRIN1 overexpression weakened CDK1’s interaction with the inhibitory kinase MYT1, while enhancing its association with the activating phosphatase Cdc25C (Fig. 2H). This indicates GPRIN1 orchestrates CDK1 activation by physically steering it away from an inhibitory complex and toward an activating one.

Functionally, CDK1 knockdown abolished the GPRIN1-induced acceleration of the G2/M transition (Fig. 2I). CDK1 depletion also reversed GPRIN1-driven molecular changes, including increased Cyclin B1 and Cdc25C expression and reduced inhibitory phosphorylation of Cdc25C at Ser216 (Fig. 2J). Together, these findings establish CDK1 as a required downstream mediator of GPRIN1 signaling in GBC.

### CDK1 is required for GPRIN1-mediated maintenance of mitochondrial homeostasis and mitophagy

**Fig. 3:**
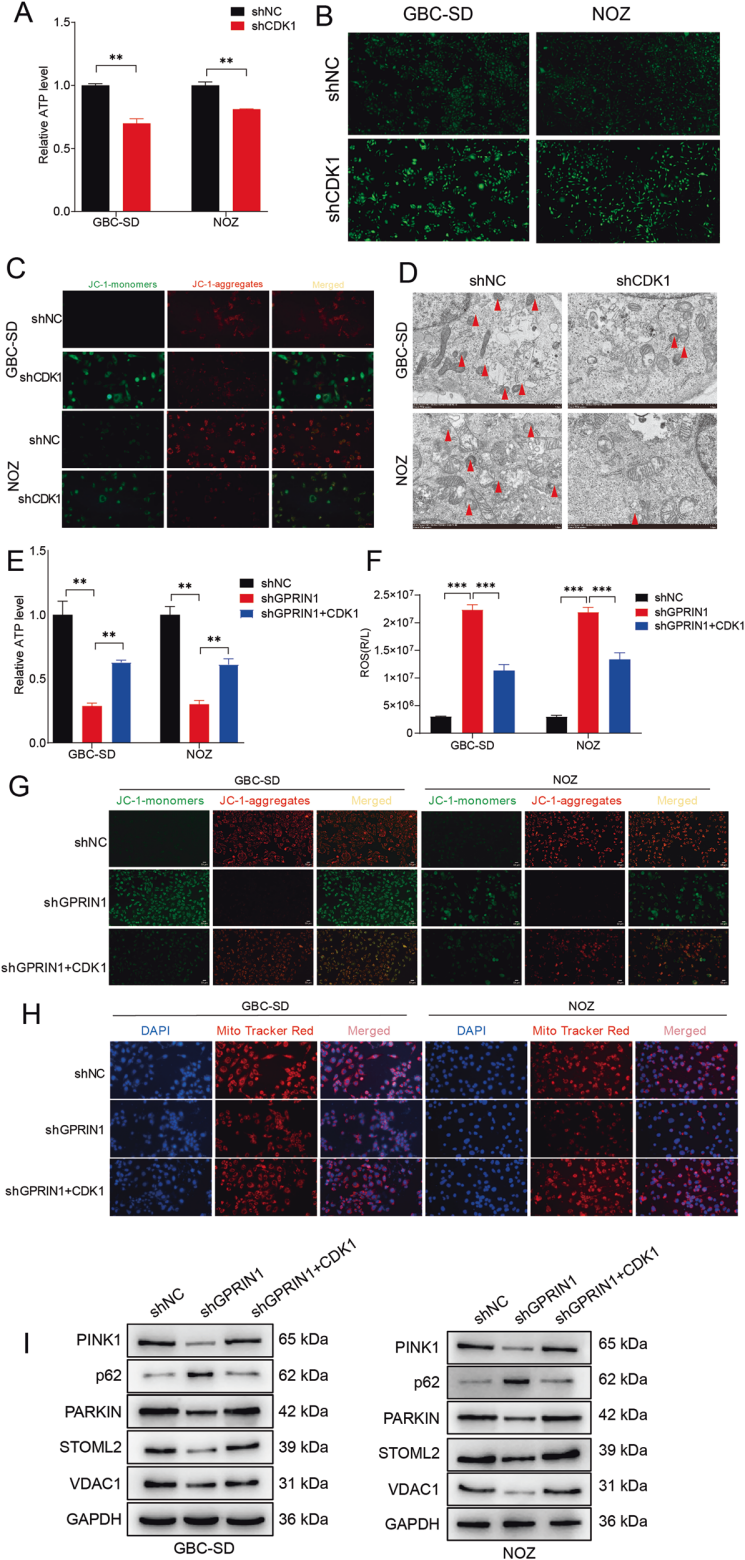
GPRIN1 maintains mitochondrial homeostasis and promotes mitophagy via CDK1 in GBC cells. **A** Relative ATP levels in GBC-SD and NOZ cells with CDK1 knockdown (shCDK1 vs. shNC). **B** Cellular green fluorescence patterns in GBC-SD and NOZ cells with CDK1 knockdown. Scale bar = 100 µm. **C** JC-1 fluorescence microscopy of GBC-SD and NOZ cells with CDK1 knockdown. Scale bar = 50 µm. **D** TEM images of GBC-SD and NOZ cells with CDK1 knockdown. Red arrowheads: mitophagosomes. Scale bars indicated. Relative ATP levels (**E**) and ROS levels (**F**) in GBC-SD and NOZ cells: shNC, shGPRIN1, or shGPRIN1 + CDK1 overexpression. **G** JC-1 fluorescence microscopy of GBC-SD and NOZ cells under conditions in (**E**). Scale bar = 50 µm. **H** MitoTracker Red and DAPI staining in GBC-SD and NOZ cells under conditions in (**E**). Scale bar = 50 µm. **I** Western blots for mitophagy proteins in GBC-SD and NOZ cells under conditions in (**E**). *P < 0.05, **P < 0.01, ***P < 0.001.

Having established that GPRIN1 regulates CDK1 activity and is independently critical for mitochondrial homeostasis, we next sought to determine whether CDK1 acts as the downstream mediator of GPRIN1’s effects on mitochondria. Initially, we examined the direct impact of CDK1 on mitochondrial function. Genetic depletion of CDK1 in GBC cells (Supplementary Fig. S3A, B) significantly reduced intracellular ATP levels (Fig. 3A), induced profound mitochondrial dysfunction as evidenced by changes in cellular fluorescence (Fig. 3B), and caused a marked loss of MMP, revealed by a shift from red to green fluorescence in JC-1 staining assays (Fig. 3C). TEM further showed that CDK1 knockdown resulted in severe mitochondrial damage, characterized by swollen organelles with disorganized cristae, and a concomitant decrease in the formation of mitophagosomes (Fig. 3D). These data confirm that CDK1 itself is essential for maintaining mitochondrial integrity and function in GBC cells [34, 35].

To test the hypothesis that the mitochondrial defects caused by GPRIN1 loss are mediated through CDK1, we performed a series of rescue experiments. As previously shown, GPRIN1 depletion severely impaired mitochondrial bioenergetics and stalled mitophagy (Fig. 1). Critically, re-expression of CDK1 in GPRIN1-depleted cells partially rescued the key mitochondrial defects, significantly restoring ATP production (Fig. 3E) and attenuating the excessive accumulation of ROS (Fig. 3F), and re-established the mitochondrial membrane potential, as indicated by JC-1 staining (Fig. 3G). Furthermore, the reduced mitochondrial mass observed in GPRIN1-knockdown cells was also partially restored upon CDK1 overexpression, suggesting a recovery of a healthier mitochondrial network (Fig. 3H).

Crucially, we investigated whether CDK1 could rescue the impaired mitophagy flux. GPRIN1 depletion led to a paradoxical state of mitochondrial damage coupled with reduced levels of the mitophagy initiators PINK1 and PARKIN and an accumulation of the autophagy receptor p62. Strikingly, CDK1 re-expression was sufficient to rescue this stalled mitophagy flux, leading to a partial restoration of PINK1 and PARKIN levels and a decrease in p62 accumulation (Fig. 3I).

Taken together, these rescue experiments establish a pivotal GPRIN1-CDK1 axis in GBC, wherein CDK1 acts as the indispensable downstream effector coupling cell cycle progression to mitochondrial quality control.

### GPRIN1 drives CDK1 transcription by enhancing E2F1 stability and nuclear function

**Fig. 4:**
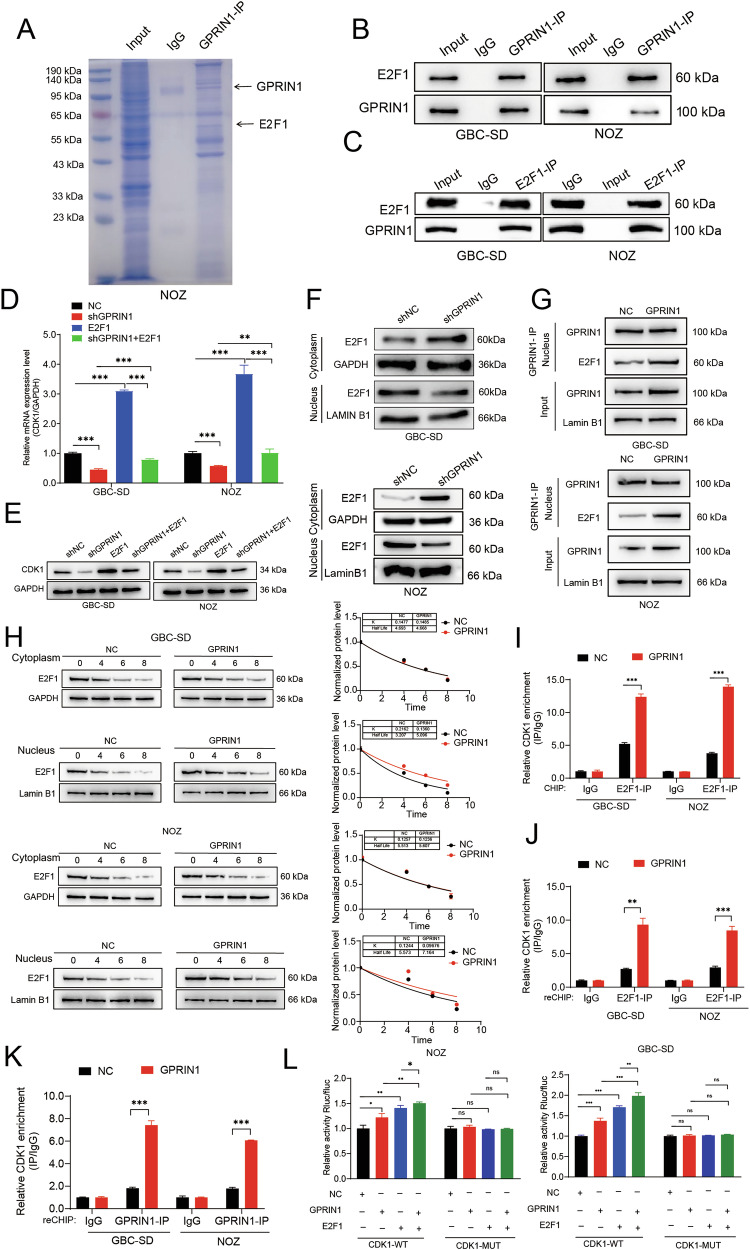
GPRIN1 promotes CDK1 transcription via E2F1 nuclear activity and stability. **A** Coomassie blue stain of GPRIN1-IP from NOZ lysates; GPRIN1 and E2F1 identified by MS. Co-IP of endogenous (**B**) GPRIN1 with E2F1 and (**C**) E2F1 with GPRIN1 in GBC-SD and NOZ cells. **D** Relative CDK1 mRNA in GBC-SD and NOZ cells: NC, shGPRIN1, E2F1, or shGPRIN1 + E2F1. **E** Western blot for CDK1 under conditions in (**D**). **F** Western blot for E2F1 in cytoplasmic/nuclear fractions of GBC-SD and NOZ cells with GPRIN1 knockdown. GAPDH/Lamin B1 as controls. **G** Co-IP of GPRIN1 with E2F1 in nuclear lysates of GBC-SD and NOZ cells with GPRIN1 overexpression. **H** Western blot for E2F1 stability in cytoplasmic and nuclear fractions of GBC-SD and NOZ cells with GPRIN1 overexpression after CHX treatment (0–8 h). Quantification is shown on the right. **I** ChIP assay for E2F1 enrichment on CDK1 promoter in GBC-SD and NOZ cells with GPRIN1 overexpression. Re-ChIP for co-occupancy of (**J**) GPRIN1 then E2F1, and (**K**) E2F1 then GPRIN1, on CDK1 promoter in GBC-SD and NOZ cells. **L** CDK1 promoter (WT or MUT E2F1 sites) luciferase activity in NOZ and GBC-SD cells with NC, GPRIN1, E2F1, or GPRIN1 + E2F1. *P < 0.05, **P < 0.01, ***P < 0.001.

Having established that GPRIN1 controls CDK1 abundance and activity, we next sought to determine the underlying mechanism. Given that GPRIN1 modulates CDK1 mRNA levels (Fig. S3C, D), we hypothesized it might cooperate with a transcription factor. To identify this partner, we performed an IP mass spectrometry screen and discovered the master cell cycle regulator E2F1 as a top candidate GPRIN1 interactor (Fig. 4A). We confirmed a bona fide interaction between endogenous GPRIN1 and E2F1 in both GBC-SD and NOZ cells through reciprocal co-immunoprecipitation (Fig. 4B, C). Given that E2F1 is a well-established transcriptional activator of CDK1 [36], we hypothesized that GPRIN1 drives CDK1 expression by potentiating E2F1 activity. Rescue experiments showed that GPRIN1 depletion alone suppressed CDK1 mRNA and protein levels, an effect that was partially but significantly rescued by the overexpression of E2F1 in both GBC-SD and NOZ cells (Fig. 4D, E). These results establish E2F1 as a critical downstream mediator of GPRIN1 and establish that GPRIN1-mediated transcriptional control of CDK1 is dependent on E2F1. We next sought to understand the underlying mechanism. Cellular fractionation revealed that GPRIN1 depletion reduced the nuclear pool of E2F1, suggesting GPRIN1 promotes its nuclear accumulation or retention (Fig. 4F). Co-IP from nuclear lysates confirmed their physical interaction specifically within the nuclear compartment (Fig. 4G). To distinguish whether GPRIN1 promotes E2F1 nuclear translocation or enhances its stability within the nucleus, we performed a cycloheximide (CHX) chase assay coupled with nuclear and cytoplasmic fractionation. The results were definitive: GPRIN1 overexpression markedly prolonged the half-life of E2F1 specifically within the nuclear fraction, while having no effect on the stability of cytoplasmic E2F1 (Fig. 4H). This provides direct evidence that GPRIN1’s primary function is to selectively protect nuclear E2F1 from proteolytic degradation, rather than promoting its nuclear import.

Having established this mechanism of stabilization, the critical next step was to determine if GPRIN1 and E2F1 form a functional transcriptional complex at their target site. ChIP-PCR assays revealed GPRIN1 overexpression enhanced the binding of E2F1 to the CDK1 promoter (Fig. 4I). Furthermore, sequential Re-ChIP provided conclusive evidence of a ternary complex, demonstrating that GPRIN1 and E2F1 co-occupy the same DNA fragment of the CDK1 promoter (Fig. 4J, K). Finally, a luciferase reporter assay showed that GPRIN1 and E2F1 synergistically drove reporter expression from a wild-type CDK1 promoter, an effect that was completely abolished upon mutation of the E2F1 binding sites (Fig. 4L). Importantly, this transcriptional mechanism appears to operate independently of GPRIN1’s effect on CDK1 phosphorylation, as direct modulation of E2F1 levels did not alter the phosphorylation of CDK1 at key regulatory sites or affect CDC25C phosphatase activity (Fig. S4A–D). Collectively, GPRIN1 interacts with and stabilizes E2F1 to drive CDK1 expression, a process that runs in parallel to its post-translational control of CDK1 activity.

### Dual-level regulation of CDK1 by GPRIN1 through spatially distinct transcriptional and post-translational mechanisms

**Fig. 5:**
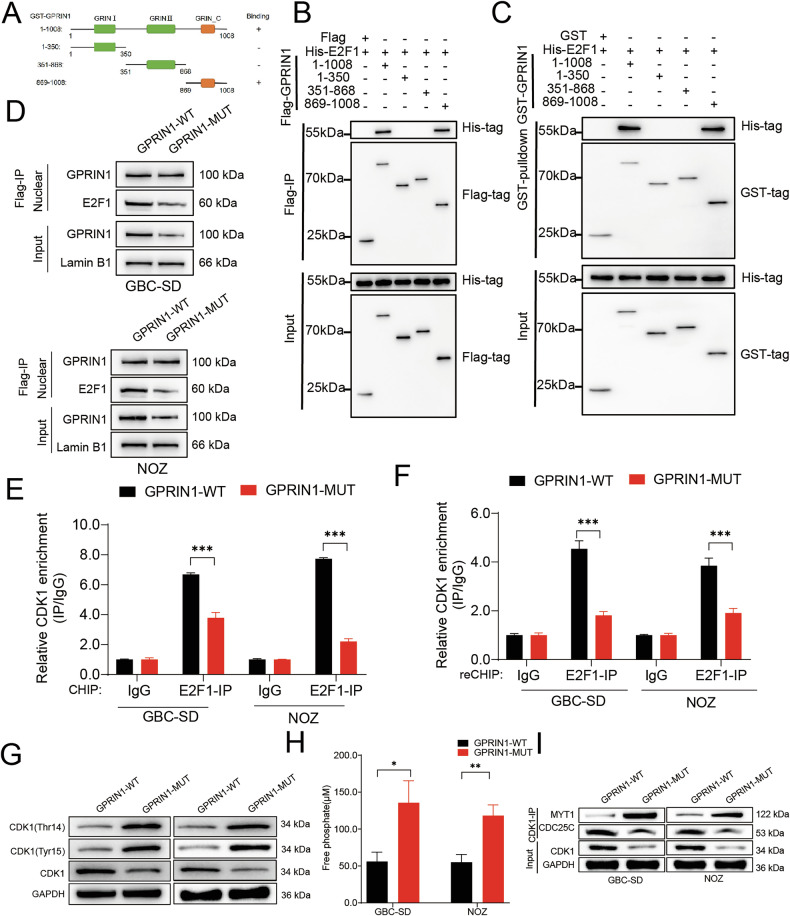
GPRIN1 C-terminus mediates E2F1 interaction for transcriptional co-regulation and is essential for post-translational CDK1 activation. **A** Schematic of GPRIN1 truncations and E2F1 binding summary. **B** Co-IP of His-E2F1 with GFP-GPRIN1 full-length or truncations. **C** GST pulldown of His-E2F1 with GST-GPRIN1 full-length or truncations. **D** Co-IP of Flag-GPRIN1-WT or -MUT with endogenous E2F1 in NOZ and GBC-SD cells. **E** ChIP for E2F1 enrichment on CDK1 promoter in GBC-SD and NOZ cells with GPRIN1-WT or -MUT. **F** Re-ChIP for GPRIN1 and E2F1 co-occupancy on CDK1 promoter in GBC-SD and NOZ cells with GPRIN1-WT or -MUT. **G** Western blot for CDK1 phosphorylation (Thr14, Tyr15) and total CDK1 in GBC-SD and NOZ cells with GPRIN1-WT or -MUT. **H** Intracellular free phosphate levels in GBC-SD and NOZ cells with GPRIN1-WT or -MUT. **I** Co-IP of CDK1 with MYT1 or CDC25C in GBC-SD and NOZ cells with GPRIN1-WT or -MUT. *P < 0.05, **P < 0.01, ***P < 0.001.

To dissect the GPRIN1-E2F1 interaction, we mapped the specific GPRIN1 domain responsible for binding E2F1. We generated a series of GPRIN1 truncation mutants covering the N-terminal, central, and C-terminal regions, with domain boundaries defined according to the UniProt database (https://www.uniprot.org/) (Fig. 5A). Co-IP assays using these fragments revealed that the interaction with E2F1 is mediated exclusively by the C-terminus of GPRIN1 (Fig. 5B). This finding was further corroborated by GST-pull down assays (Fig. 5C), demonstrating that the C-terminal region of GPRIN1 is both necessary and sufficient for its interaction with E2F1.

To functionally validate the importance of this specific domain, we engineered a GPRIN1 mutant (GPRIN1-MUT) with a deletion of this C-terminal E2F1-binding region and compared its function to wild-type GPRIN1 (GPRIN1-WT). As expected, Co-IP experiments from nuclear lysates confirmed that GPRIN1-MUT exhibited markedly diminished binding to E2F1 compared to GPRIN1-WT (Fig. 5D). We next tested whether this interaction was required for GPRIN1’s co-activator function at the CDK1 promoter. GPRIN1-WT robustly enhanced E2F1 occupancy at the CDK1 promoter. In stark contrast, this effect was completely abrogated in cells expressing the GPRIN1-MUT (Fig. 5E). Sequential re-ChIP assays also provided GPRIN1-WT, but not GPRIN1-MUT, significantly increased the co-occupancy of GPRIN1 and E2F1 on the promoter (Fig. 5F). These findings solidify a model where GPRIN1 drives CDK1 transcription through a direct, C-terminal-mediated interaction with E2F1.

This domain-specific mutant provided a unique tool to determine the functional extent of the C-terminal region. When we examined its direct impact on CDK1 phosphorylation, we found that GPRIN1-MUT, which cannot bind E2F1, also lost the ability to regulate CDK1 activity in the cytoplasm. Specifically, unlike the wild-type protein, GPRIN1-MUT failed to reduce the inhibitory phosphorylation of CDK1 at Thr14 and Tyr15 (Fig. 5G). Consistently, the mutant was unable to induce the decrease in intracellular free phosphate levels observed with the wild-type protein (Fig. 5H). Furthermore, GPRIN1-MUT lost the ability to remodel CDK1’s protein interactions; it failed to dissociate CDK1 from the inhibitor MYT1 and could not enhance its binding to the activator Cdc25C (Fig. 5I). In summary, our findings reveal that GPRIN1 orchestrates CDK1 activation through a dual mechanism that is crucially dependent on its C-terminus. In the nucleus, this domain binds E2F1 to drive CDK1 transcription. In the cytoplasm, this same domain is indispensable for steering the CDK1 protein towards its active state by controlling its regulatory partners.

### The GPRIN1-CDK1 axis promotes GBC phenotypes by activating PI3K-Akt signaling

**Fig. 6:**
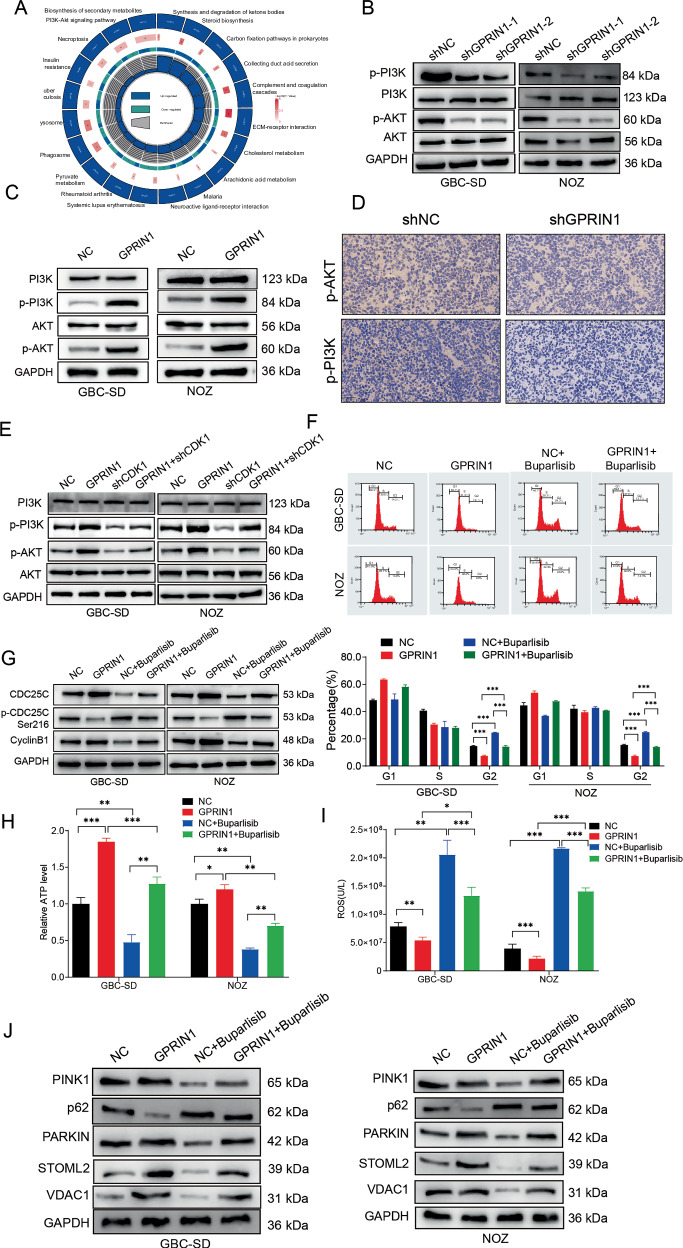
GPRIN1 activates PI3K-Akt signaling, essential for its GBC pro-tumorigenic effects. **A** Pathway enrichment (circos plot) of proteomic changes upon GPRIN1 manipulation, highlighting PI3K-Akt. Western blots for p-PI3K, PI3K, p-Akt, Akt in GBC-SD and NOZ cells with (**B**) GPRIN1 knockdown (shGPRIN1-1, shGPRIN1-2 vs. shNC) or (**C**) GPRIN1 overexpression (GPRIN1 vs. NC). **D** IHC for p-Akt and p-PI3K in shGPRIN1 vs. shNC xenograft tumors (*n* = 5 mice/group). Scale bar = 50 µm. **E** Western blots for PI3K/Akt pathway proteins in GBC-SD and NOZ cells: NC, GPRIN1, shCDK1, or GPRIN1+shCDK1. **F** Flow cytometry cell cycle analysis of GBC-SD and NOZ cells (NC or GPRIN1) ± Buparlisib. **G** Western blots for CDC25C, p-CDC25C, CyclinB1 under conditions in (**F**). **H** Relative ATP levels and (**I**) ROS levels in GBC-SD and NOZ cells under conditions in (**F**). **J** Western blots for mitophagy proteins under conditions in (**F**).*P < 0.05, **P < 0.01, ***P < 0.001.

Our findings establish the GPRIN1-CDK1 axis as a critical nexus linking cell cycle control with mitochondrial quality. To understand how this axis exerts such broad influence, we next aimed to map its downstream effector networks. Pathway enrichment analysis of our proteomics data identified the PI3K-Akt signaling cascade, a central regulator of cell survival and metabolism, as a top candidate pathway robustly activated by GPRIN1 (Fig. 6A). To validate this connection, we first assessed the phosphorylation status of key nodes within the pathway. Consistent with our proteomic data, GPRIN1 knockdown markedly attenuated, while its overexpression robustly enhanced, the activating phosphorylation of both PI3K and its canonical downstream target, Akt (Fig. 6B, C). This direct regulatory relationship was further corroborated in vivo, where tumor xenografts from GPRIN1-knockdown cells displayed a profound decrease in p-PI3K and p-Akt levels compared to control tumors (Fig. 6D). Remarkably, the ability of GPRIN1 to induce PI3K and Akt phosphorylation was completely abrogated by concurrent CDK1 knockdown (Fig. 6E). This critical finding demonstrates that PI3K-Akt activation is not a parallel event but a direct downstream consequence of GPRIN1-mediated CDK1 hyperactivity. To definitively establish the functional importance of this entire GPRIN1-CDK1-PI3K/Akt cascade, we employed pharmacological inhibition to disrupt the terminal node. Treatment with the PI3K inhibitor Buparlisib was sufficient to completely abolish GPRIN1’s multifaceted oncogenic effects, reversing its promotion of G2/M progression, its enhancement of ATP production, and its protective effect against oxidative stress, while also disrupting its regulation of mitophagy (Fig. 6F–J). Taken together, our data reveal GPRIN1-driven CDK1 hyperactivity is required to engage the PI3K-Akt cascade, which then functions as a central hub to coordinate diverse oncogenic outputs.

### GPRIN1 promotes GBC tumor growth in vivo through a CDK1-PI3K/Akt axis

**Fig. 7:**
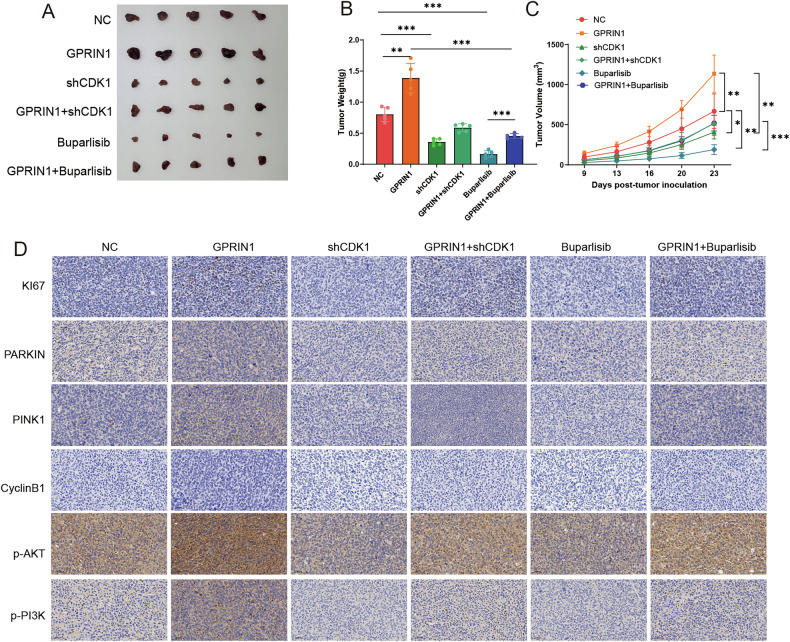
GPRIN1 promotes GBC tumor growth in vivo via CDK1 and PI3K-Akt signaling. **A** Macroscopic images of xenograft tumors from GBC-SD cells: NC, GPRIN1, shCDK1, GPRIN1+shCDK1, Buparlisib, or GPRIN1+Buparlisib (*n* = 5 mice/group). **B** Tumor weights from NC, GPRIN1, shCDK1, GPRIN1+shCDK1, Buparlisib, or GPRIN1+Buparlisib groups. P-values indicated. **C** Tumor growth curves for NC, GPRIN1, shCDK1, GPRIN1+shCDK1, Buparlisib, or GPRIN1+Buparlisib. *P*-values indicated. **D** IHC for Ki67, PARKIN, PINK1, CyclinB1, p-Akt, p-PI3K in tumor sections from (**A**). Scale bar = 50 µm.*P < 0.05, **P < 0.01, ***P < 0.001.

Our previous work suggested that GPRIN1 is essential for GBC tumor growth in vivo [24]. To determine if this pro-tumorigenic function is mechanistically dependent on GPRIN1-CDK1-PI3K/Akt axis, we conducted a series of in vivo rescue and inhibition experiments. As expected, the accelerated tumorigenesis driven by GPRIN1 was completely rescued by the concurrent genetic knockdown of CDK1. Strikingly, pharmacological inhibition of the terminal node, PI3K, with Buparlisib perfectly phenocopied this rescue effect, entirely neutralizing the growth advantage conferred by GPRIN1(Fig. 7A–C). Together, these data demonstrate that GPRIN1-mediated tumor promotion in vivo is strictly dependent on the downstream CDK1-PI3K/Akt pathway. To elucidate the molecular underpinnings of these macroscopic growth phenotypes, we performed IHC on tumor sections. As anticipated, tumors overexpressing GPRIN1 exhibited a clear signature of aggressive growth, including heightened proliferation markers (Ki67, CyclinB1), upregulated mitophagy components (PARKIN, PINK1), and strong activation of the PI3K-Akt signaling cascade (p-Akt, p-PI3K). The elevated levels of all these markers were driven back to basal conditions by both the genetic knockdown of CDK1 and inhibition of PI3K (Fig. 7D). This demonstrates that the molecular state of the tumor is as dependent on the GPRIN1-CDK1-PI3K/Akt axis as its physical growth. In conclusion, these in vivo data establish that GPRIN1 promotes GBC progression by activating a hierarchical CDK1-PI3K/Akt cascade to orchestrate key oncogenic programs.

### GPRIN1 is upregulated in GBC and correlates with poor clinical prognosis

**Fig. 8:**
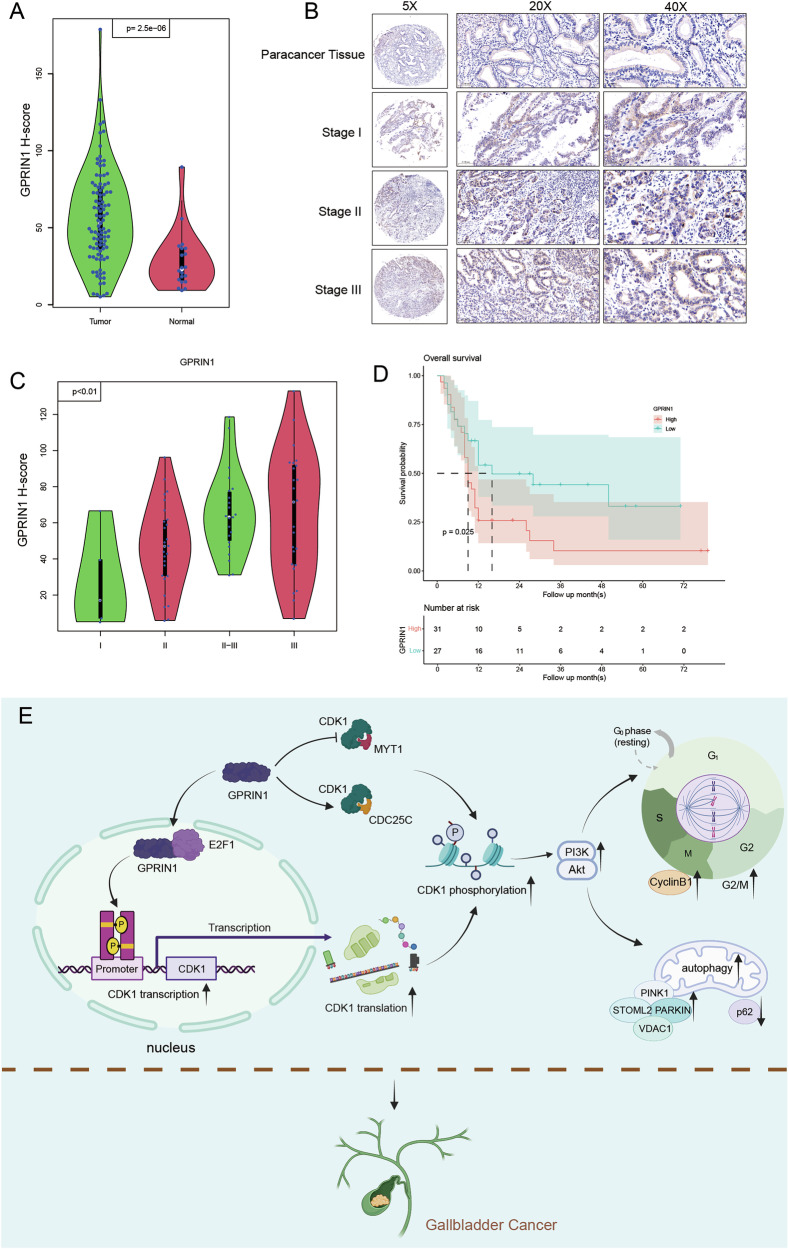
GPRIN1 is upregulated in GBC, correlates with poor prognosis; proposed model. **A** Violin plot of GPRIN1 H-scores in GBC tumor vs. normal tissues. *p* < 0.001. **B** Representative GPRIN1 IHC in paracancerous and GBC Stage I-III tissues (5X, 20X, 40X). Scale bars indicated. **C** Violin plot of GPRIN1 H-scores by GBC clinical stage. *p* < 0.01. **D** Kaplan–Meier overall survival curves for GBC patients stratified by GPRIN1 expression levels determined by quantitative H-score. The cohort was dichotomized into ‘High’ and ‘Low’ expression groups using the median H-score as the cutoff. *p* = 0.025, log-rank test. **E** Proposed model of GPRIN1’s role in GBC progression via E2F1/CDK1 and PI3K/AKT pathways.

To establish the clinical relevance of our mechanistic findings, we investigated the expression and prognostic significance of GPRIN1 in a cohort of GBC patient tissues. IHC analysis revealed that GPRIN1 protein expression was significantly elevated in GBC tumor tissues compared to adjacent non-tumorous counterparts (Fig. 8A). Critically, this upregulation was not static; GPRIN1 staining intensity showed a strong positive correlation with advanced TNM stage (Fig. 8B, C), indicating that GPRIN1 levels increase in lockstep with disease progression. Kaplan-Meier survival analysis demonstrated unequivocally that patients within the high-GPRIN1 expression group experienced significantly shorter overall survival (Fig. 8D). These clinical data therefore identify GPRIN1 as a potent prognostic biomarker and strongly suggest that its elevated expression is a key driver of GBC malignancy.

Finally, our study converges on a comprehensive model that mechanistically explains how GPRIN1 drives GBC progression (Fig. 8E). In summary, our study reveals a dual mechanism for GPRIN1-driven GBC progression. In the nucleus, GPRIN1 binds and stabilizes E2F1 to drive CDK1 transcription. In parallel, it directly promotes CDK1’s activation post-translationally. This hyperactivated CDK1 then propels the G2/M cell cycle transition and switches on PI3K-Akt signaling. Ultimately, this axis couples cell proliferation with mitochondrial quality control to fuel aggressive tumor growth.

## Discussion

Gallbladder Cancer (GBC) remains a highly aggressive malignancy with limited therapeutic avenues and a dismal prognosis [37, 38]. While the dysregulation of the cell cycle and a reliance on mitochondrial homeostasis are known dependencies in GBC,a coordinated regulator that links these two processes has not been clearly defined [39, 40]. In this study, we identify GPRIN1 as a previously unrecognized regulator capable of integrating these pathways. We demonstrate that GPRIN1 executes a bimodal regulatory program—employing both transcriptional co-activation and post-translational modulation—to promote CDK1 activation. This GPRIN1–CDK1 axis supports cell-cycle progression and mitochondrial quality control via PI3K/Akt signaling, suggesting potential value as a prognostic indicator and therapeutic target in GBC.

A key mechanistic insight from our study is the discovery of a previously uncharacterized, dual-level regulatory control that GPRIN1 exerts over CDK1. While aberrant CDK1 activity is a hallmark of GBC [41, 42], the mechanisms for its sustained activation are poorly understood. Our work refines the canonical E2F1–CDK1 transcriptional model by identifying GPRIN1 as a nuclear co-activator that enhances E2F1 stability and function [43, 44]. Importantly, this transcriptional control is complemented by a parallel post-translational mechanism, where GPRIN1 physically steers CDK1 away from its inhibitor MYT1 and toward its activator Cdc25C. This dual-layered regulation provides a coherent framework to facilitate CDK1 activation, highlighting the GPRIN1–CDK1 interface as a potential point of therapeutic intervention.

Our investigation also provides significant new insights into mitochondrial quality control in GBC, a largely unexplored research area. We establish that GPRIN1 is essential for maintaining mitochondrial health. Critically, we observed that GPRIN1 loss leads to a paradoxical state of severe mitochondrial damage coupled with the suppression of the canonical PINK1/PARKIN-mediated mitophagy pathway [45, 46]. This finding of ‘stalled mitophagic flux’ distinguishes our work from studies in other cancers that report simple on/off regulation of mitophagy [47, 48], suggesting that GBC cells may depend on a tightly regulated mitophagy equilibrium. This reveals a potential vulnerability in GPRIN1-high tumors, positioning GPRIN1 as a candidate biomarker for mitochondrial-targeted therapies.

We further demonstrate that these two fundamental processes—CDK1 activation and mitochondrial maintenance—are linked through the PI3K/Akt pathway. To our knowledge, this is the first study to identify an upstream factor that connects CDK1 activation with PI3K/Akt signaling in GBC. The resulting positive regulatory circuit may contribute to sustaining proliferative and survival signals, indicating that GBC cells could be dependent on the GPRIN1–CDK1–PI3K/Akt axis and highlighting a potential therapeutic strategy for disrupting this pathway.

The elucidation of this axis carries clinical and translational implications. Our work identifies high GPRIN1 expression as a potential prognostic biomarker and provides a rationale for therapeutically targeting this pathway. The anti-tumor efficacy observed with the PI3K inhibitor Buparlisib in our models highlights the feasibility of such approaches [49]. Moreover, our findings support a biomarker-guided strategy for applying CDK1 or PI3K inhibitors in GPRIN1-high GBC, supplementing current empirical treatment regimens [50, 51].

Despite the comprehensive nature of our study, several limitations remain, including the need for validation in patient-derived organoids and larger clinical cohorts [52–54]. Future work should also explore the upstream regulators of GPRIN1 and delineate the downstream PI3K/Akt effectors that mediate mitophagy regulation.

In conclusion, this study identifies GPRIN1 as an important regulator in GBC and provides three key contributions: (i) GPRIN1 couples cell-cycle progression with mitochondrial homeostasis; (ii) we delineate a GPRIN1–E2F1–CDK1–PI3K/Akt signaling network governed by a positive regulatory loop; and (iii) we identify GPRIN1 as a potential biomarker and therapeutic target. Together, these findings offer a more integrated view of GBC pathobiology and provide a foundation for biomarker-driven therapeutic strategies.

## Supplementary information

Supplement WB

Supplement Table

Supplementary Figure legends

Figure S1

Figure S2

Figure S3

Figure S4

## Data Availability

The datasets generated and analysed during the current study are not publicly available due to data ownership and confidentiality considerations but are available from the corresponding author on reasonable request. The original Western blot images can be found in Supplementary WB.

## References

[1] Valle JW, Kelley RK, Nervi B, Oh DY, Zhu AX. Biliary tract cancer. Lancet. 2021;397:428–44. 33516341 10.1016/S0140-6736(21)00153-7

[2] Roa JC, García P, Kapoor VK, Maithel SK, Javle M, Koshiol J. Gallbladder cancer. Nat Rev Dis Prim. 2022;8:69. 36302789 10.1038/s41572-022-00398-y PMC12314663

[3] de Bitter TJJ, de Reuver PR, de Savornin Lohman EAJ, Kroeze LI, Vink-Börger ME, van Vliet S, et al. Comprehensive clinicopathological and genomic profiling of gallbladder cancer reveals actionable targets in half of patients. NPJ Precis Oncol. 2022;6:83. 36335173 10.1038/s41698-022-00327-y PMC9637208

[4] Yan S, Liu Z, Wang T, Sui Y, Wu X, Shen J, et al. Super-enhancer reprograming driven by SOX9 and TCF7L2 represents transcription-targeted therapeutic vulnerability for treating gallbladder cancer. Adv Sci (Weinh). 2024;11:e2406448. 39492805 10.1002/advs.202406448 PMC11653766

[5] Ao J, Hu M, Wang J, Jiang X. Advancing biliary tract malignancy treatment: emerging frontiers in cell-based therapies. Front Immunol. 2025;16:1559465. 40013133 10.3389/fimmu.2025.1559465 PMC11862832

[6] Ciccarone F, Ciriolo MR. Reprogrammed mitochondria: a central hub of cancer cell metabolism. Biochem Soc Trans. 2024;52:1305–15. 38716960 10.1042/BST20231090

[7] Stoolman JS, Porcelli AM, Martínez-Reyes I. Editorial: Mitochondria as a hub in cellular signaling. Front Cell Dev Biol. 2022;10:981464. 36046344 10.3389/fcell.2022.981464 PMC9421365

[8] Chen F, Xue Y, Zhang W, Zhou H, Zhou Z, Chen T, et al. The role of mitochondria in tumor metastasis and advances in mitochondria-targeted cancer therapy. Cancer Metastasis Rev. 2024;43:1419–43. 39307891 10.1007/s10555-024-10211-9 PMC11554835

[9] Liu Y, Sun Y, Guo Y, Shi X, Chen X, Feng W, et al. An overview: the diversified role of mitochondria in cancer metabolism. Int J Biol Sci. 2023;19:897–915. 36778129 10.7150/ijbs.81609 PMC9910000

[10] Arif T, Shteinfer-Kuzmine A, Shoshan-Barmatz V. Decoding Cancer through Silencing the Mitochondrial Gatekeeper VDAC1. Biomolecules. 2024;14:1304. 39456237 10.3390/biom14101304 PMC11506819

[11] Hao X, Bu W, Lv G, Xu L, Hou D, Wang J, et al. Disrupted mitochondrial homeostasis coupled with mitotic arrest generates antineoplastic oxidative stress. Oncogene. 2022;41:427–43. 34773075 10.1038/s41388-021-02105-9 PMC8755538

[12] Roca-Portoles A, Tait SWG. Mitochondrial quality control: from molecule to organelle. Cell Mol Life Sci. 2021;78:3853–66. 33782711 10.1007/s00018-021-03775-0 PMC8106605

[13] Qiu YH, Zhang TS, Wang XW, Wang MY, Zhao WX, Zhou HM, et al. Mitochondria autophagy: a potential target for cancer therapy. J Drug Target. 2021;29:576–91. 33554661 10.1080/1061186X.2020.1867992

[14] Deepak K, Roy PK, Das CK, Mukherjee B, Mandal M. Mitophagy at the crossroads of cancer development: Exploring the role of mitophagy in tumor progression and therapy resistance. Biochim Biophys Acta Mol Cell Res. 2024;1871:119752. 38776987 10.1016/j.bbamcr.2024.119752

[15] Glytsou C, Chen X, Zacharioudakis E, Al-Santli W, Zhou H, Nadorp B, et al. Mitophagy promotes resistance to BH3 mimetics in acute myeloid leukemia. Cancer Discov. 2023;13:1656–77. 37088914 10.1158/2159-8290.CD-22-0601 PMC10330144

[16] Zheng XX, Chen JJ, Sun YB, Chen TQ, Wang J, Yu SC. Mitochondria in cancer stem cells: Achilles heel or hard armor. Trends Cell Biol. 2023;33:708–27. 37137792 10.1016/j.tcb.2023.03.009

[17] Ren L, Yang Y, Li W, Zheng X, Liu J, Li S, et al. CDK1 serves as a therapeutic target of adrenocortical carcinoma via regulating epithelial-mesenchymal transition, G2/M phase transition, and PANoptosis. J Transl Med. 2022;20:444. 36184616 10.1186/s12967-022-03641-y PMC9528181

[18] Yu L, Wei J, Liu P. Attacking the PI3K/Akt/mTOR signaling pathway for targeted therapeutic treatment in human cancer. Semin Cancer Biol. 2022;85:69–94. 34175443 10.1016/j.semcancer.2021.06.019

[19] Tewari D, Patni P, Bishayee A, Sah AN, Bishayee A. Natural products targeting the PI3K-Akt-mTOR signaling pathway in cancer: a novel therapeutic strategy. Semin Cancer Biol. 2022;80:1–17. 31866476 10.1016/j.semcancer.2019.12.008

[20] Shi Z, Tian L, Qiang T, Li J, Xing Y, Ren X, et al. From structure modification to drug launch: a systematic review of the ongoing development of cyclin-dependent kinase inhibitors for multiple cancer therapy. J Med Chem. 2022;65:6390–418. 35485642 10.1021/acs.jmedchem.1c02064

[21] Nordman JC, Phillips WS, Kodama N, Clark SG, Del Negro CA, Kabbani N. Axon targeting of the alpha 7 nicotinic receptor in developing hippocampal neurons by Gprin1 regulates growth. J Neurochem. 2014;129:649–62. 24350810 10.1111/jnc.12641 PMC4496803

[22] Jiang F, Yang L, Jiao X. Dynamic network biomarker to determine the critical point of breast cancer stage progression. Breast Cancer. 2023;30:453–65. 36807044 10.1007/s12282-023-01438-5

[23] Cui X, Zhang B, Li B, Li X. Circular RNA circ_0002360 regulates the Taxol resistance and malignant behaviors of Taxol-resistant non-small cell lung cancer cells by microRNA-585-3p-dependent modulation of G protein regulated inducer of neurite outgrowth 1. Bioengineered. 2022;13:9070–85. 35293280 10.1080/21655979.2022.2053803 PMC9162002

[24] Li M, Shen F, Ni X, Gong Z, Song L, Suo T, et al. GPRIN1 promotes gallbladder cancer progression through the CDK1 pathway. Research Square [Preprint].2022.

[25] Tagawa K, Homma H, Saito A, Fujita K, Chen X, Imoto S, et al. Comprehensive phosphoproteome analysis unravels the core signaling network that initiates the earliest synapse pathology in preclinical Alzheimer’s disease brain. Hum Mol Genet. 2015;24:540–58. 25231903 10.1093/hmg/ddu475

[26] Chagraoui A, Thibaut F, De Deurwaerdère P. 5-HT6 receptors: contemporary views on their neurobiological and pharmacological relevance in neuropsychiatric disorders. Dialog Clin Neurosci. 2025;27:112–28. 10.1080/19585969.2025.2502028 PMC12068339 40347153

[27] Hornbeck PV, Kornhauser JM, Tkachev S, Zhang B, Skrzypek E, Murray B, et al. PhosphoSitePlus: a comprehensive resource for investigating the structure and function of experimentally determined post-translational modifications in man and mouse. Nucleic Acids Res. 2012;40:D261–70. 22135298 10.1093/nar/gkr1122 PMC3245126

[28] Missiroli S, Perrone M, Genovese I, Pinton P, Giorgi C. Cancer metabolism and mitochondria: finding novel mechanisms to fight tumours. EBioMedicine. 2020;59:102943. 32818805 10.1016/j.ebiom.2020.102943 PMC7452656

[29] Kenny TC, Birsoy K. Mitochondria and cancer. Cold Spring Harb Perspect Med. 2024;14:a041534. 38692736 10.1101/cshperspect.a041534 PMC11610758

[30] Bankhead P, Loughrey MB, Fernández JA, Dombrowski Y, McArt DG, Dunne PD, et al. QuPath: open source software for digital pathology image analysis. Sci Rep. 2017;7:16878. 29203879 10.1038/s41598-017-17204-5 PMC5715110

[31] Narendra DP, Youle RJ. The role of PINK1-Parkin in mitochondrial quality control. Nat Cell Biol. 2024;26:1639–51. 39358449 10.1038/s41556-024-01513-9

[32] Sokhi S, Lewis CW, Bukhari AB, Hadfield J, Xiao EJ, Fung J, et al. Myt1 overexpression mediates resistance to cell cycle and DNA damage checkpoint kinase inhibitors. Front Cell Dev Biol. 2023;11:1270542. 38020882 10.3389/fcell.2023.1270542 PMC10652759

[33] Massacci G, Perfetto L, Sacco F. The Cyclin-dependent kinase 1: more than a cell cycle regulator. Br J Cancer. 2023;129:1707–16. 37898722 10.1038/s41416-023-02468-8 PMC10667339

[34] Wang Z, Fan M, Candas D, Zhang TQ, Qin L, Eldridge A, et al. Cyclin B1/Cdk1 coordinates mitochondrial respiration for cell-cycle G2/M progression. Dev Cell. 2014;29:217–32. 24746669 10.1016/j.devcel.2014.03.012 PMC4156313

[35] Liu R, Fan M, Candas D, Qin L, Zhang X, Eldridge A, et al. CDK1-mediated SIRT3 activation enhances mitochondrial function and tumor radioresistance. Mol Cancer Ther. 2015;14:2090–102. 26141949 10.1158/1535-7163.MCT-15-0017 PMC4560959

[36] Shao Z, Li C, Wu Q, Zhang X, Dai Y, Li S, et al. ZNF655 accelerates progression of pancreatic cancer by promoting the binding of E2F1 and CDK1. Oncogenesis. 2022;11:44. 35927248 10.1038/s41389-022-00418-2 PMC9352668

[37] Feo CF, Ginesu GC, Fancellu A, Perra T, Ninniri C, Deiana G, et al. Current management of incidental gallbladder cancer: a review. Int J Surg. 2022;98:106234. 35074510 10.1016/j.ijsu.2022.106234

[38] Zhou Y, Yuan K, Yang Y, Ji Z, Zhou D, Ouyang J, et al. Gallbladder cancer: current and future treatment options. Front Pharm. 2023;14:1183619. 10.3389/fphar.2023.1183619 PMC10213899 37251319

[39] Zong WX, Rabinowitz JD, White E. Mitochondria and cancer. Mol Cell. 2016;61:667–76. 26942671 10.1016/j.molcel.2016.02.011 PMC4779192

[40] Wang SF, Tseng LM, Lee HC. Role of mitochondrial alterations in human cancer progression and cancer immunity. J Biomed Sci. 2023;30:61. 37525297 10.1186/s12929-023-00956-w PMC10392014

[41] Wang Q, Bode AM, Zhang T. Targeting CDK1 in cancer: mechanisms and implications. NPJ Precis Oncol. 2023;7:58. 37311884 10.1038/s41698-023-00407-7 PMC10264400

[42] Pellarin I, Dall’Acqua A, Favero A, Segatto I, Rossi V, Crestan N, et al. Cyclin-dependent protein kinases and cell cycle regulation in biology and disease. Signal Transduct Target Ther. 2025;10:11. 39800748 10.1038/s41392-024-02080-z PMC11734941

[43] Sun M, Ji Y, Zhang G, Li Y, Dong F, Wu T. Posttranslational modifications of E2F family members in the physiological state and in cancer: roles, mechanisms and therapeutic targets. Biomed Pharmacother. 2024;178:117147. 39053422 10.1016/j.biopha.2024.117147

[44] Fischer M, Schade AE, Branigan TB, Müller GA, DeCaprio JA. Coordinating gene expression during the cell cycle. Trends Biochem Sci. 2022;47:1009–22. 35835684 10.1016/j.tibs.2022.06.007

[45] Wang S, Long H, Hou L, Feng B, Ma Z, Wu Y, et al. The mitophagy pathway and its implications in human diseases. Signal Transduct Target Ther. 2023;8:304. 37582956 10.1038/s41392-023-01503-7 PMC10427715

[46] Dong Y, Zhang X. Targeting cellular mitophagy as a strategy for human cancers. Front Cell Dev Biol. 2024;12:1431968. 39035027 10.3389/fcell.2024.1431968 PMC11257920

[47] Guan Y, Wang Y, Li B, Shen K, Li Q, Ni Y, et al. Mitophagy in carcinogenesis, drug resistance and anticancer therapeutics. Cancer Cell Int. 2021;21:350. 34225732 10.1186/s12935-021-02065-w PMC8256582

[48] Panigrahi DP, Praharaj PP, Bhol CS, Mahapatra KK, Patra S, Behera BP, et al. The emerging, multifaceted role of mitophagy in cancer and cancer therapeutics. Semin Cancer Biol. 2020;66:45–58. 31351198 10.1016/j.semcancer.2019.07.015

[49] Wang L, Yang M, Jin H. PI3K/AKT phosphorylation activates ERRα by upregulating PGC-1α and PGC-1β in gallbladder cancer. Mol Med Rep. 2021;24:613. 34184087 10.3892/mmr.2021.12252 PMC8258462

[50] De Santis MC, Gulluni F, Campa CC, Martini M, Hirsch E. Targeting PI3K signaling in cancer: challenges and advances. Biochim Biophys Acta Rev Cancer. 2019;1871:361–6. 30946868 10.1016/j.bbcan.2019.03.003

[51] Akl L, Abd El-Hafeez AA, Ibrahim TM, Salem R, Marzouk HMM, El-Domany RA, et al. Identification of novel piperazine-tethered phthalazines as selective CDK1 inhibitors endowed with in vitro anticancer activity toward the pancreatic cancer. Eur J Med Chem. 2022;243:114704. 36095992 10.1016/j.ejmech.2022.114704

[52] Qu S, Xu R, Yi G, Li Z, Zhang H, Qi S, et al. Patient-derived organoids in human cancer: a platform for fundamental research and precision medicine. Mol Biomed. 2024;5:6. 38342791 10.1186/s43556-023-00165-9 PMC10859360

[53] Jacob F, Salinas RD, Zhang DY, Nguyen PTT, Schnoll JG, Wong SZH, et al. A patient-derived glioblastoma organoid model and biobank recapitulates inter- and intra-tumoral heterogeneity. Cell. 2020;180:188–204.e22. 31883794 10.1016/j.cell.2019.11.036 PMC7556703

[54] Zhang R, Guo T, Ji L, Yin Y, Feng S, Lu W, et al. Development and application of patient-derived cancer organoidsin clinical management of gastrointestinal cancer: a state-of-the-art review. Front Oncol. 2021;11:716339. 34778032 10.3389/fonc.2021.716339 PMC8588806

