# Supplementary material for: Oncogenic GPRIN1 sustains proliferation and mitochondrial homeostasis via dual‑layer CDK1-PI3K/Akt signalling in gallbladder cancer
Source: Cell Death Dis. 2026 Mar 21;17(1):333. doi: 10.1038/s41419-026-08550-2 (PMC13039753; doi:10.1038/s41419-026-08550-2)
Supplement: Supplementary file 1 — Supplement WB [file 41419_2026_8550_MOESM1_ESM.pdf]

Figure 1 F

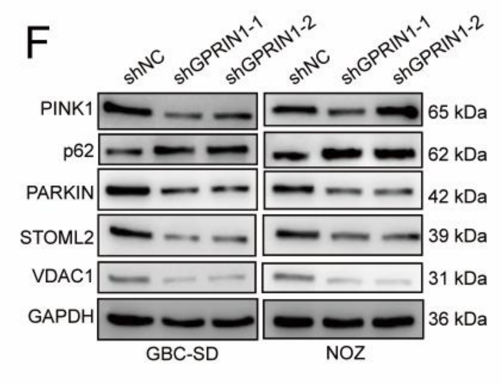

GBC-SD

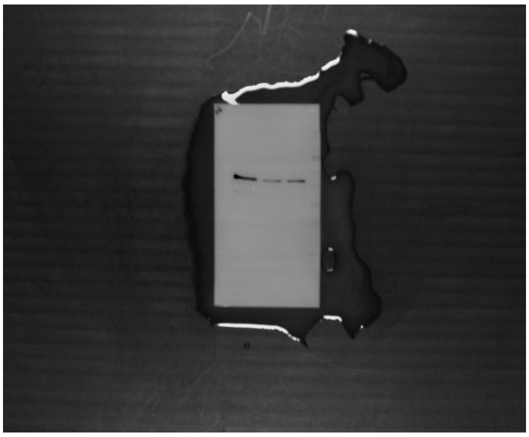

PINK1

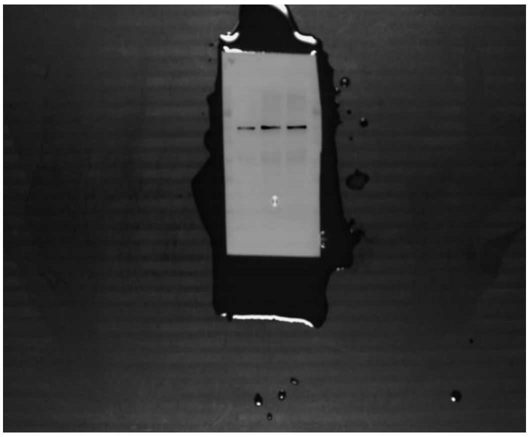

p62

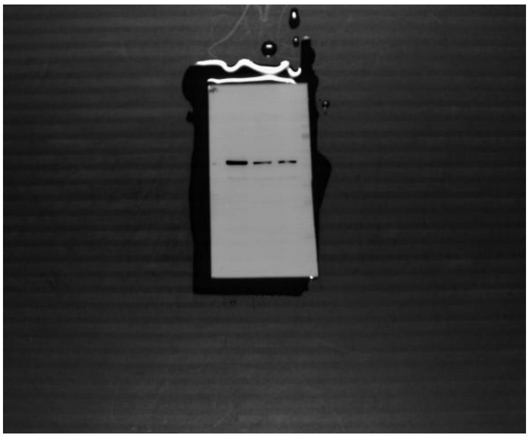

PARKIN

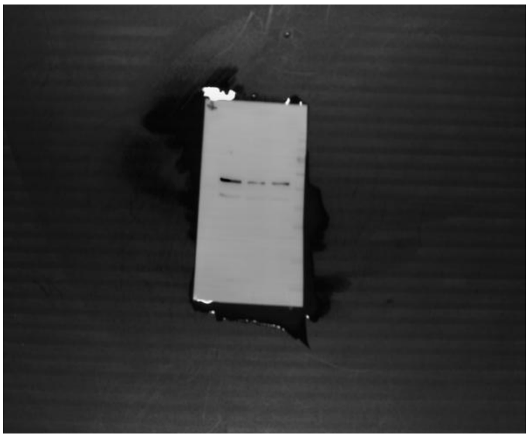

STOML2

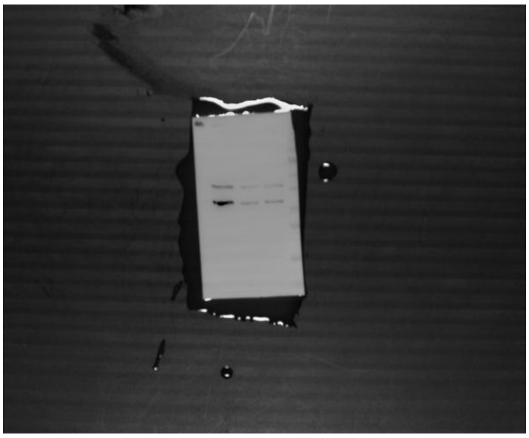

VDAC1

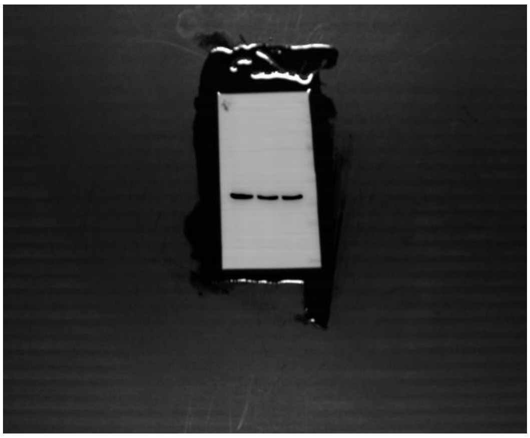

GAPDH

NOZ

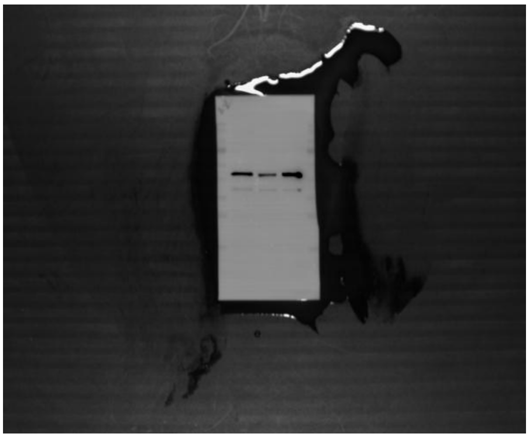

PINK1

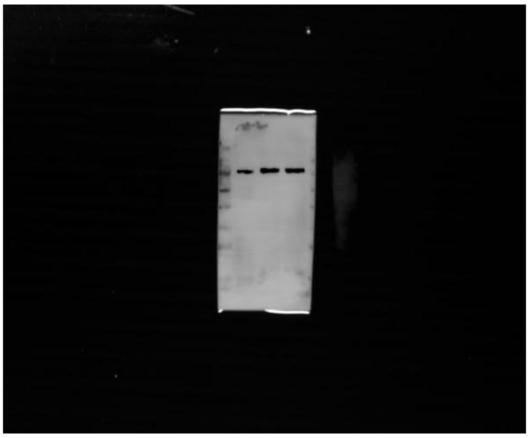

p62

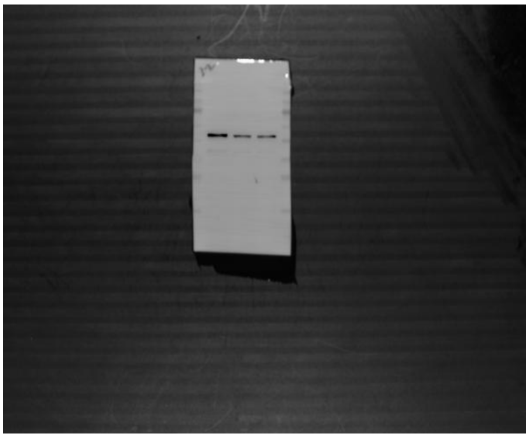

PARKIN

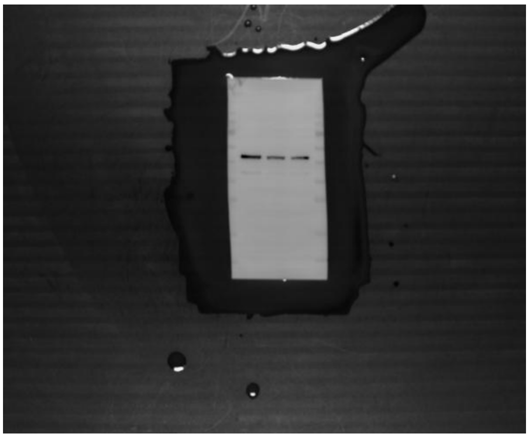

STOML2

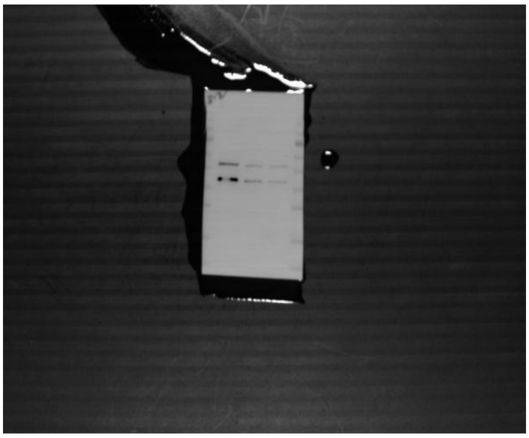

VDAC1

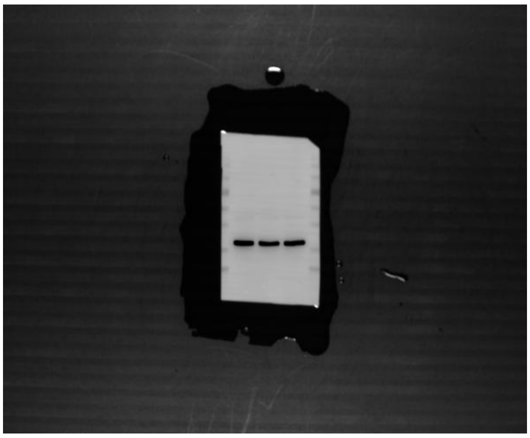

GAPDH

Figure 2 B

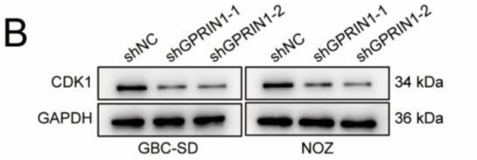

GBC-SD

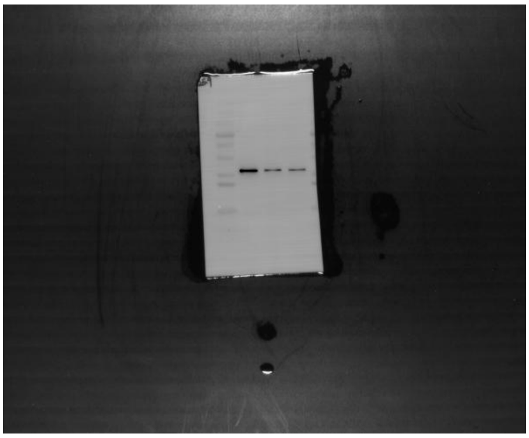

CDK1

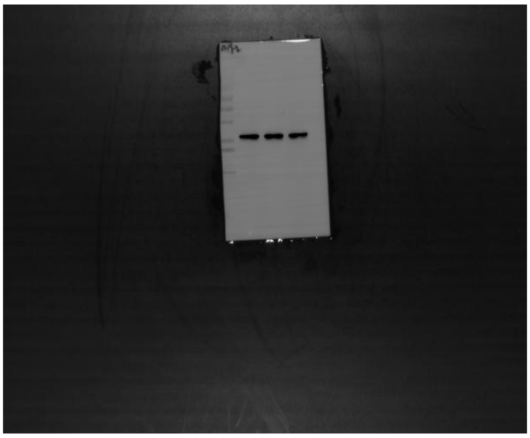

GAPDH

NOZ

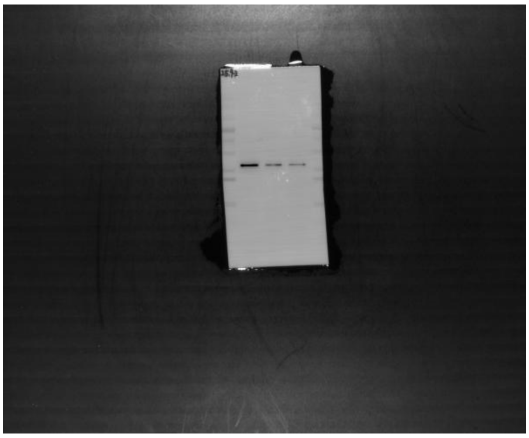

CDK1

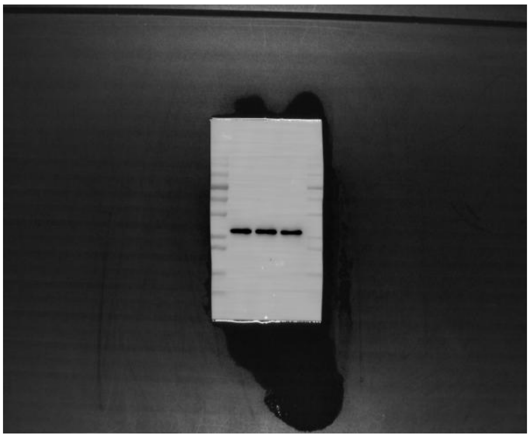

GAPDH

Figure 2 C

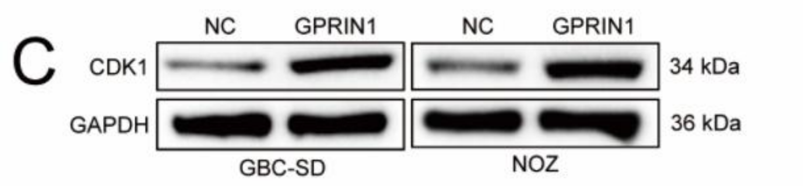

GBC-SD

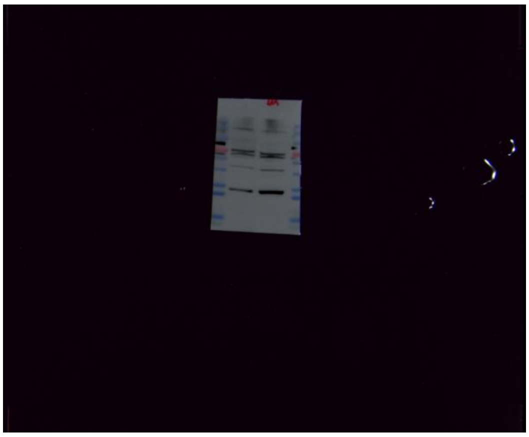

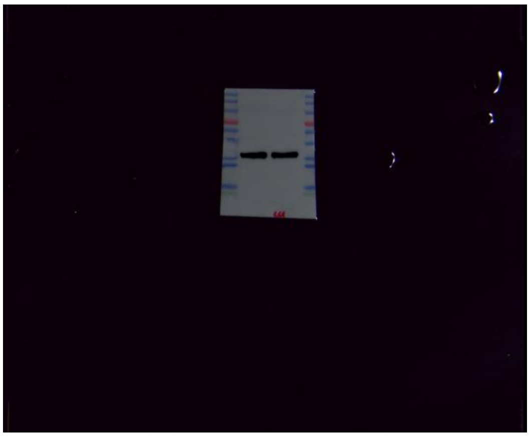

CDK1 GAPDH

NOZ

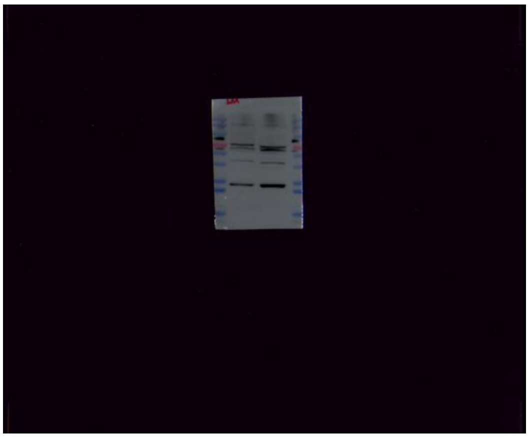

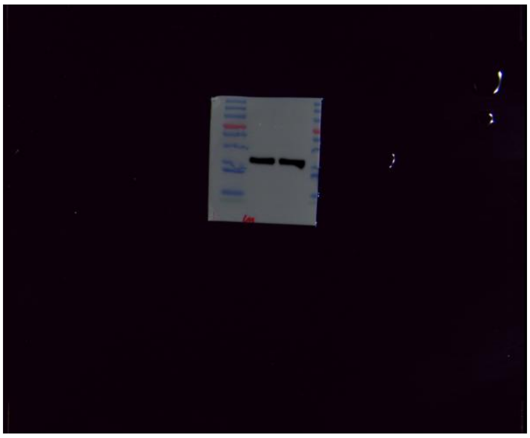

CDK1 GAPDH

Figure 2 D

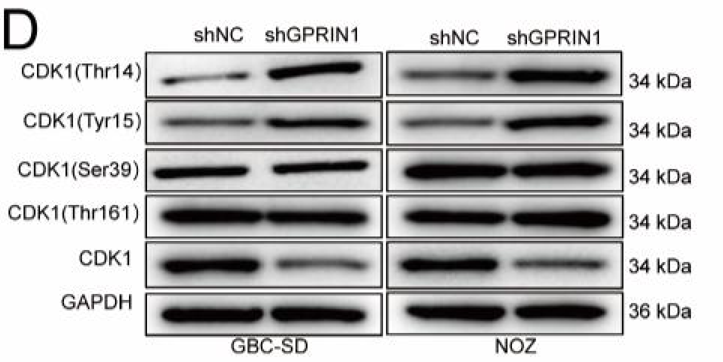

GBC-SD

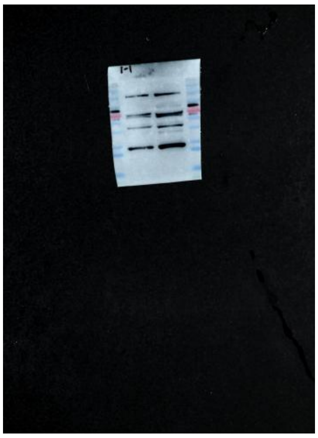

CDK1(Thr14)

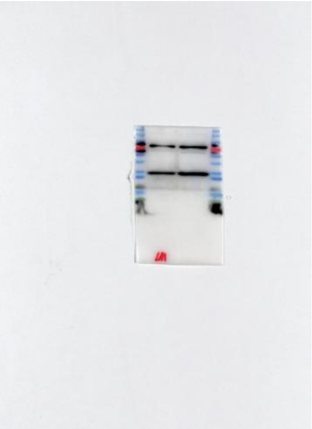

CDK1(TYr15)

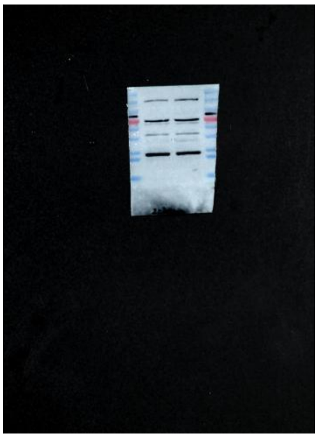

CDK1(Ser39)

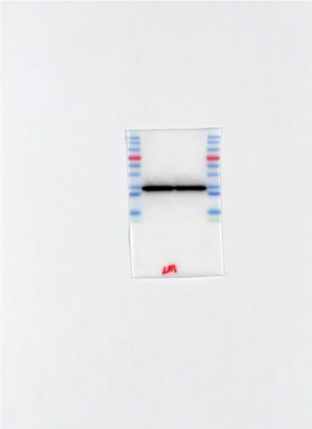

CDK1(Thr161)

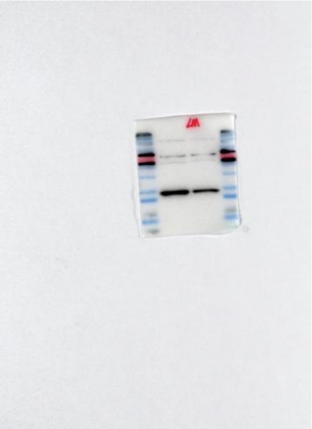

CDK1

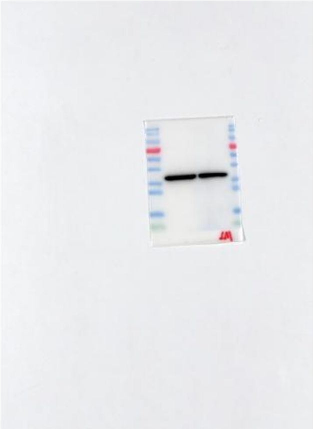

GAPDH

NOZ

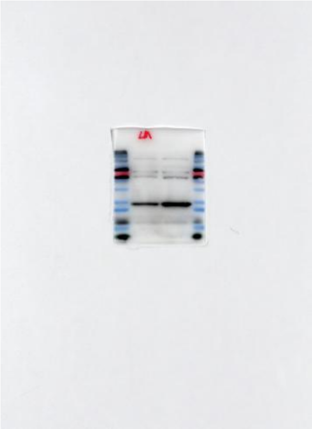

CDK1(Thr14)

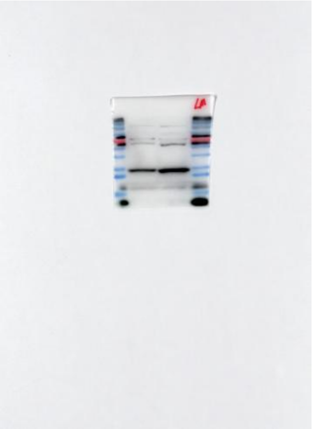

CDK1(TYr15)

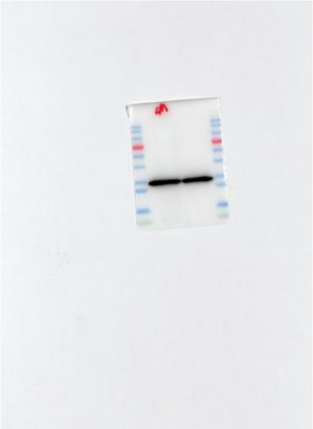

CDK1(Ser39)

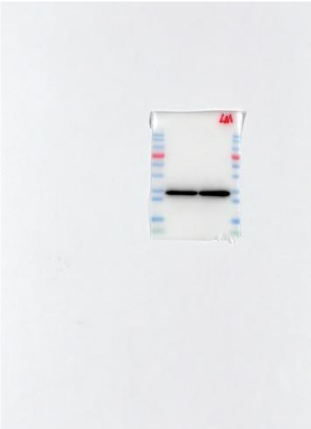

CDK1(Thr161)

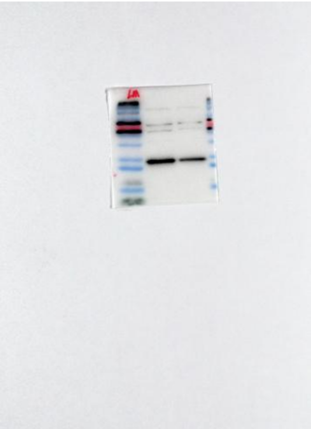

CDK1

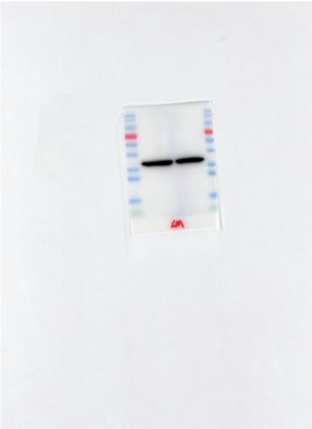

GAPDH

Figure 2 E

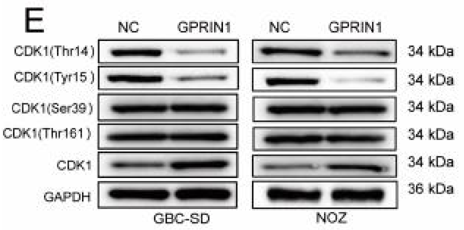

GBC-SD

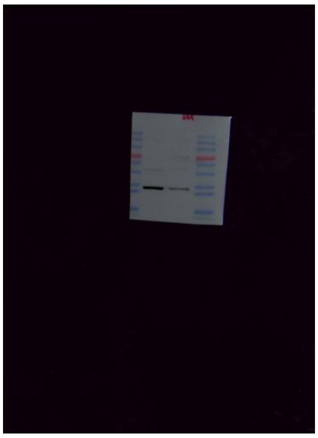

CDK1(Thr14)

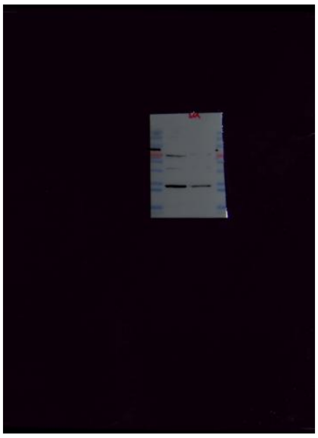

CDK1(TYr15)

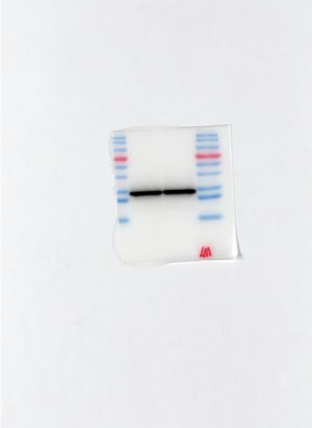

CDK1(Ser39)

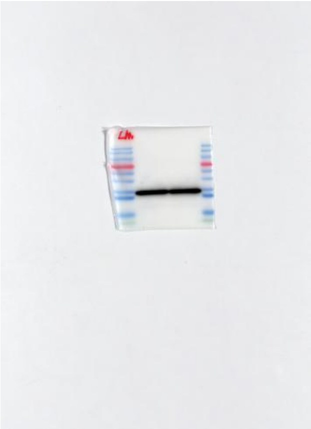

CDK1(Thr161)

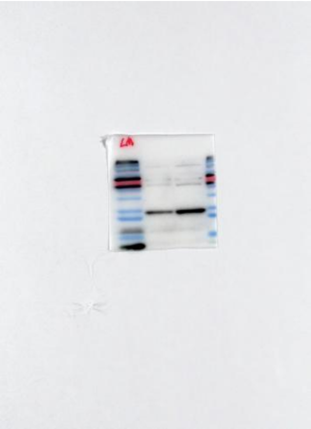

CDK1

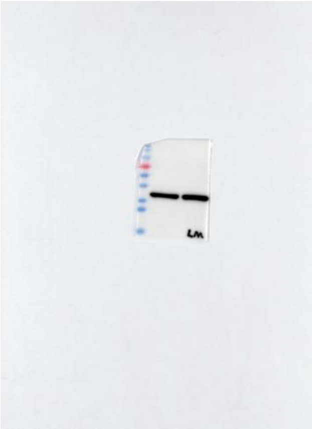

GAPDH

NOZ

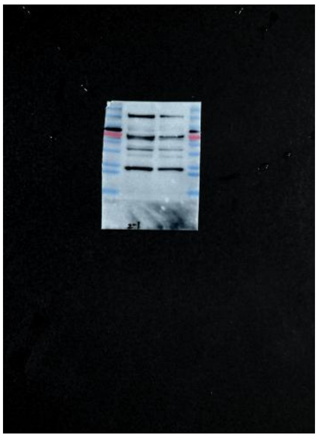

CDK1(Thr14)

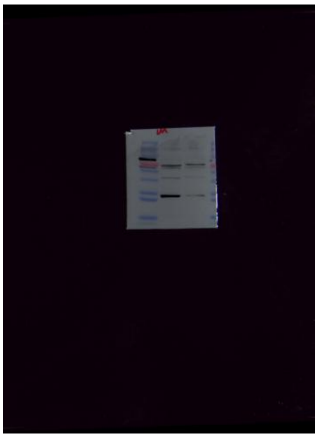

CDK1(TYr15)

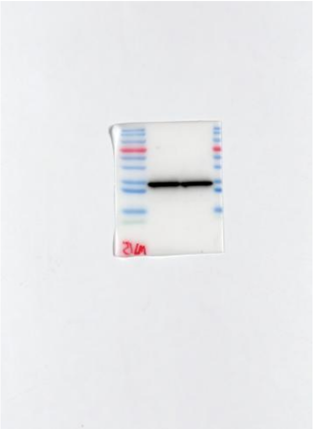

CDK1(Ser39)

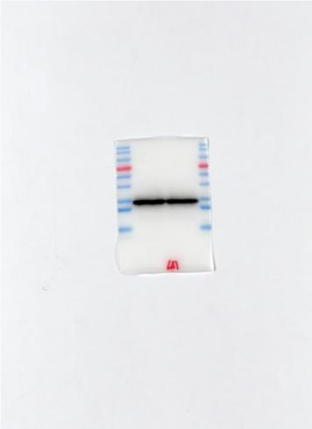

CDK1(Thr161)

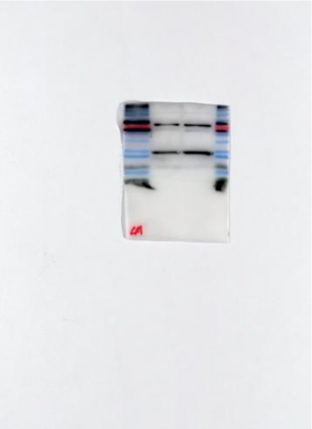

CDK1

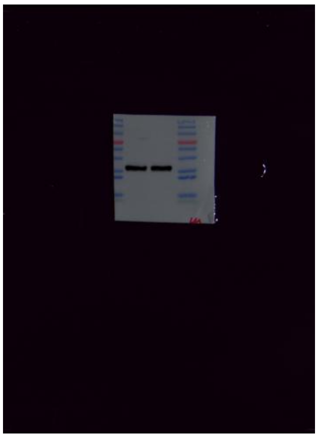

GAPDH

Figure 2 H

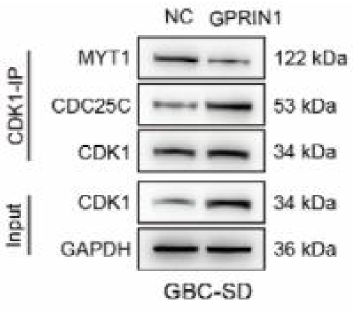

GBC-SD

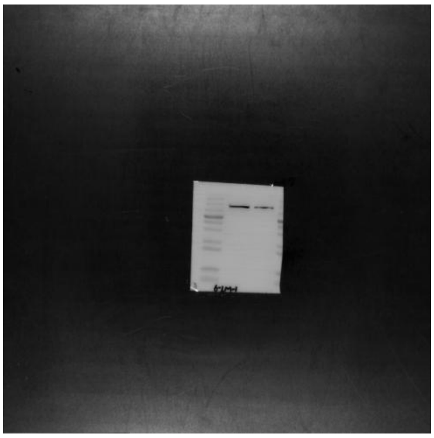

CDK1-IP-MYT1

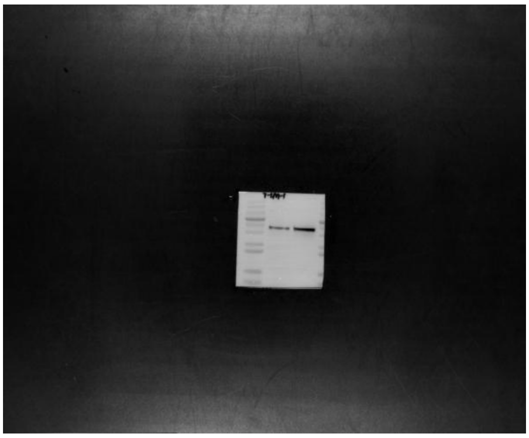

CDK1-IP-CDC25C

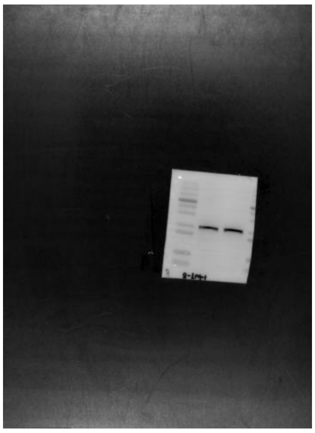

CDK1-IP

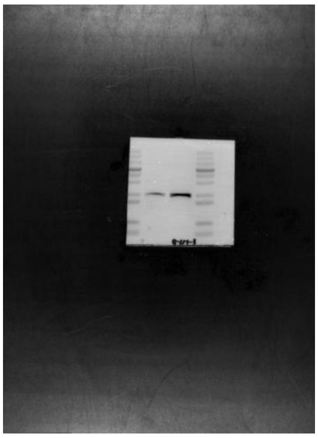

INPUT-CDKI

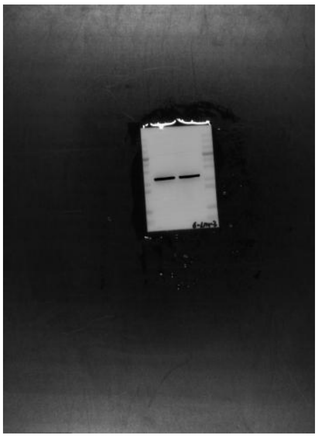

GAPDH

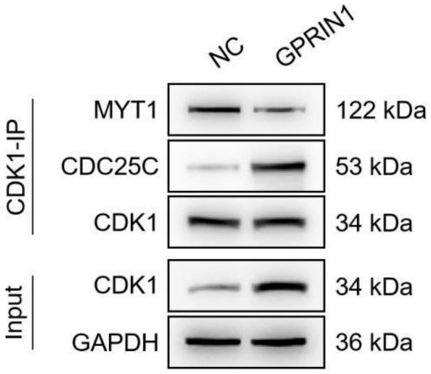

NOZ

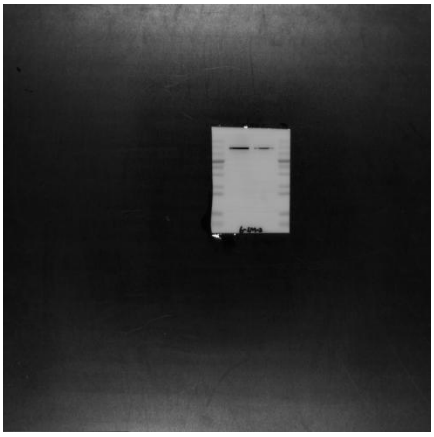

CDK1-IP-MYT1

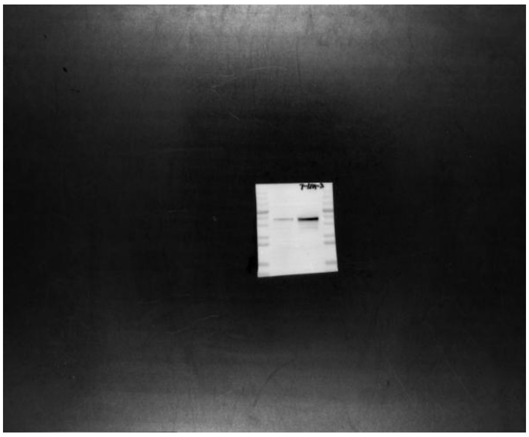

CDK1-IP-CDC25C

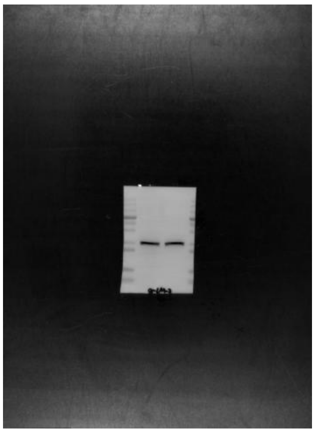

CDK1-IP

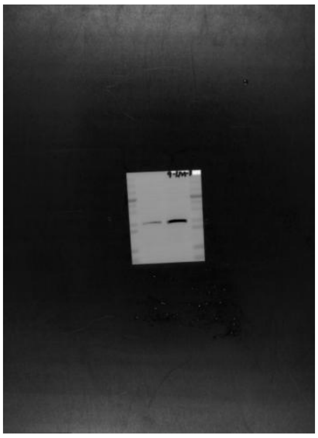

INPUT-CDKI

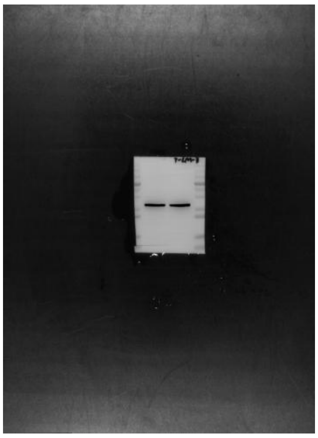

GAPDH

Figure 2 J

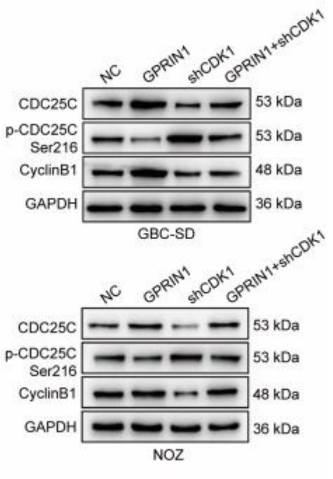

GBC-SD

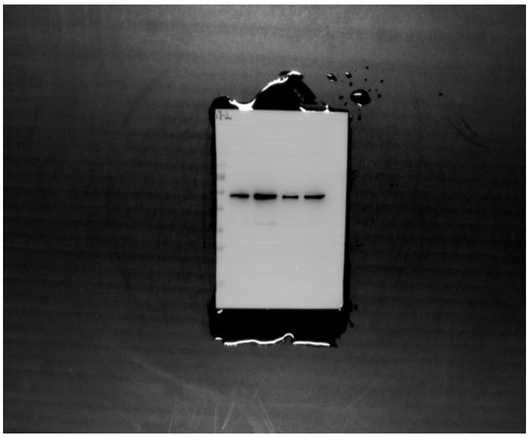

CDC25C

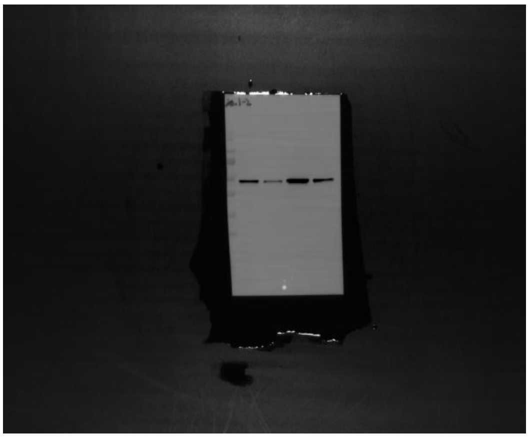

P-CDC25C

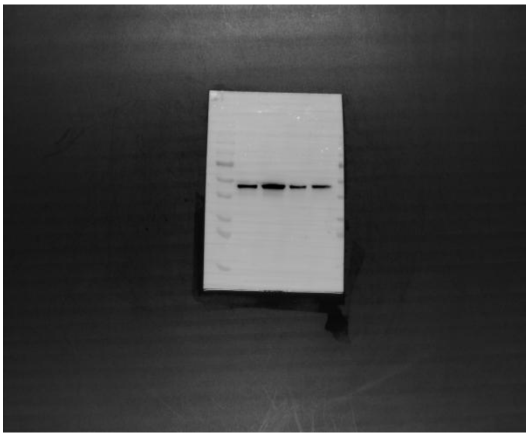

CyclinB1

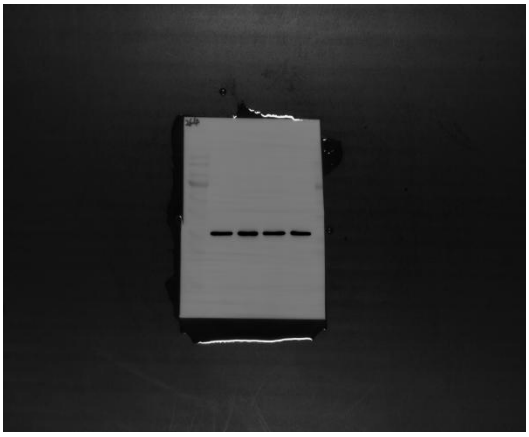

GAPDH

NOZ

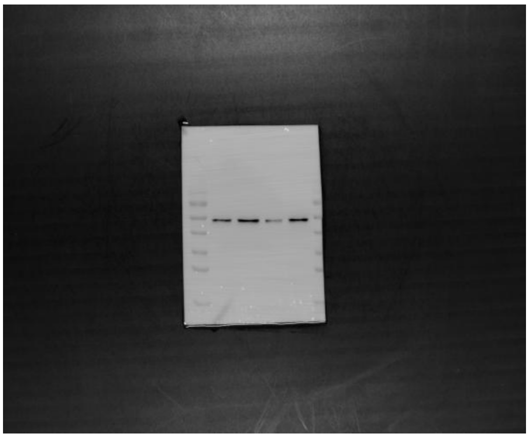

CDC25C

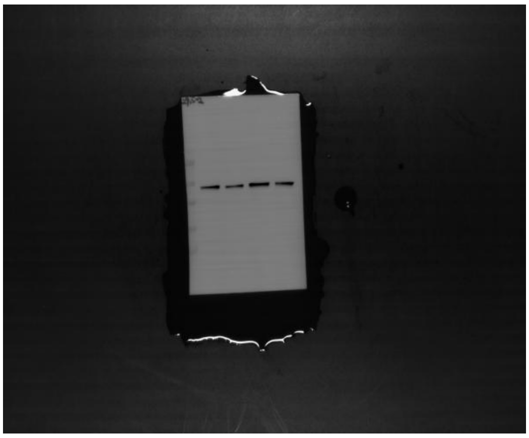

P-CDC25C

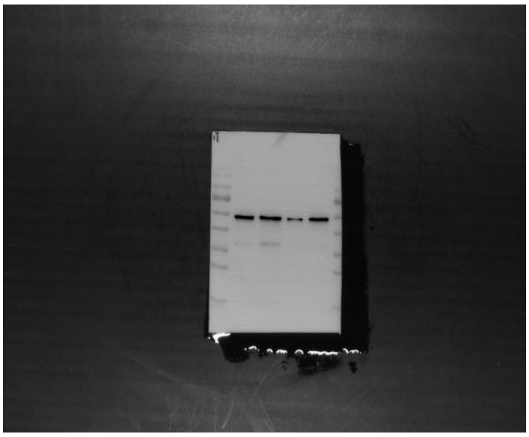

CyclinB1

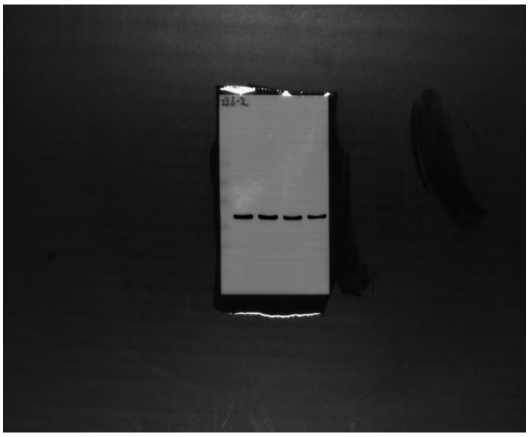

GAPDH

Figure 3 I

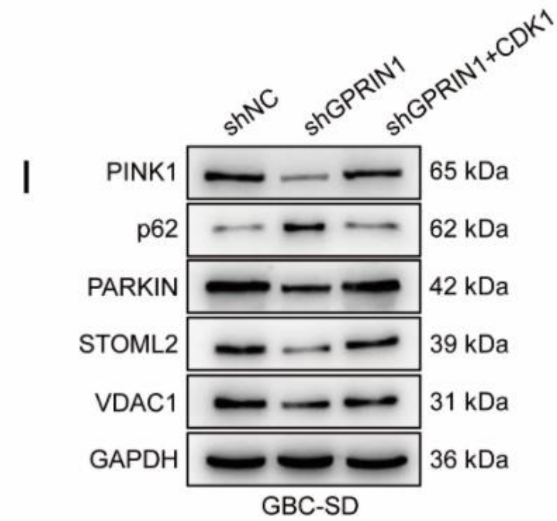

GBC-SD

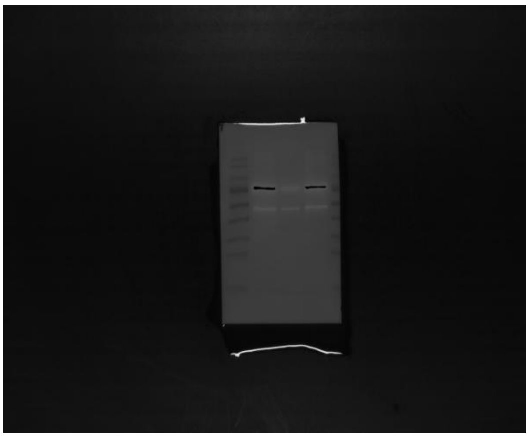

PINK1

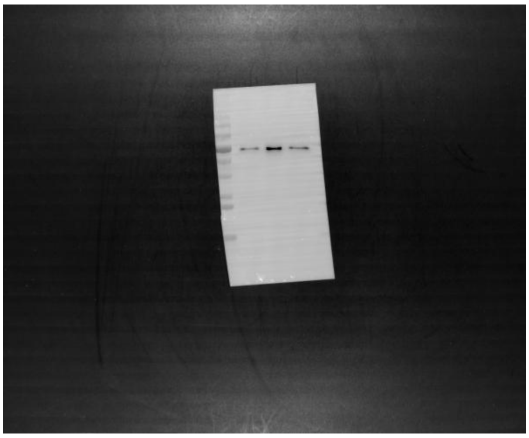

p62

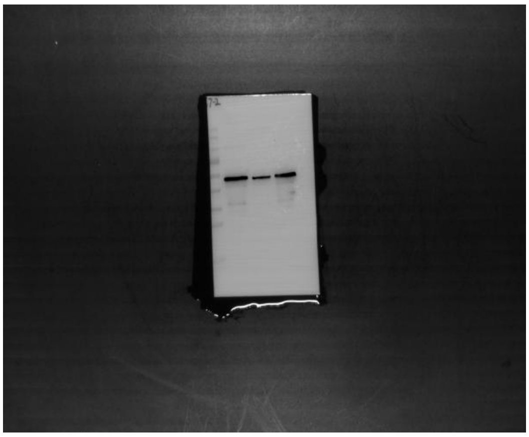

PARKIN

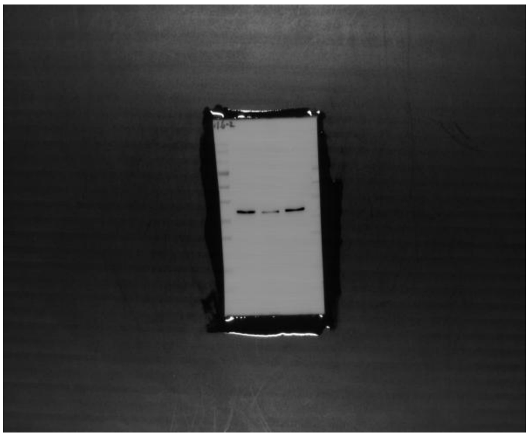

STOML2

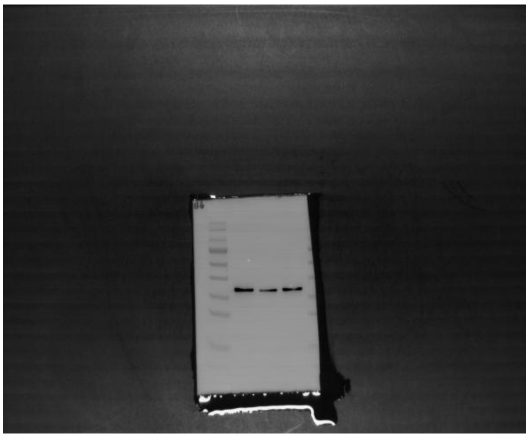

VDAC1

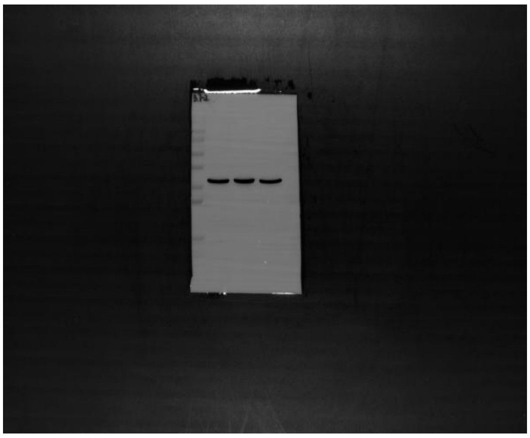

GAPDH

Figure 3 I

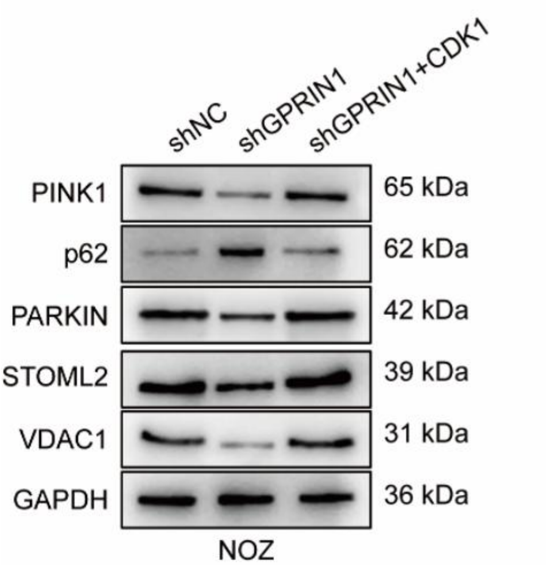

NOZ

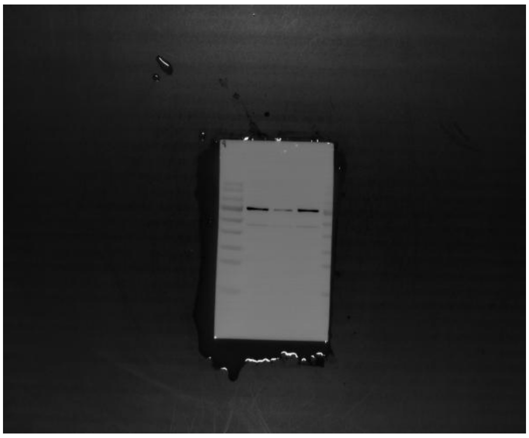

PINK1

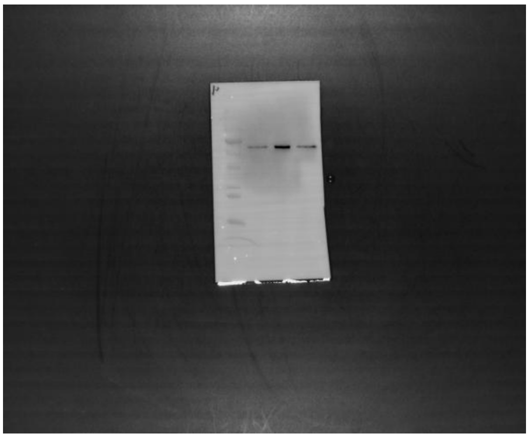

p62

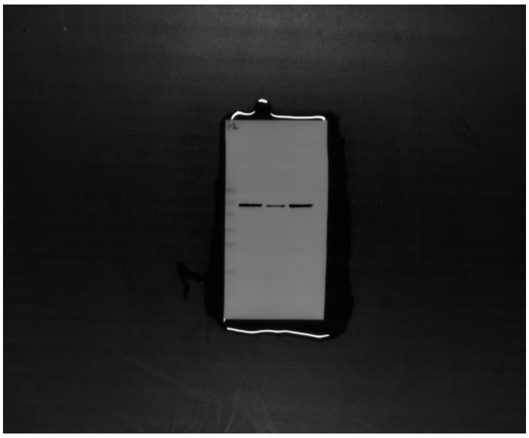

PARKIN

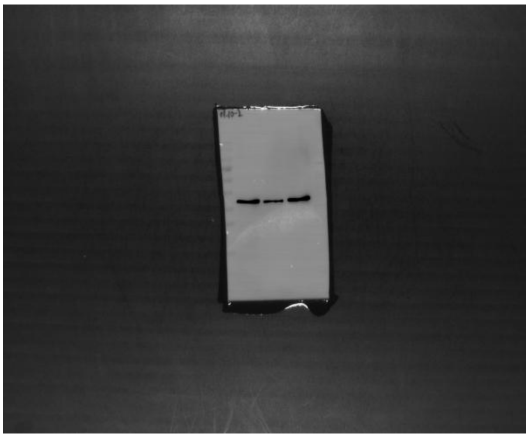

STOML2

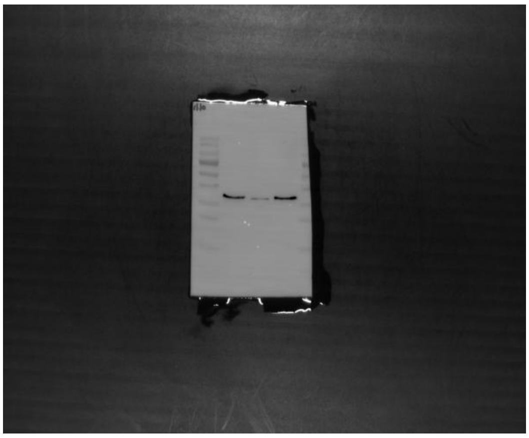

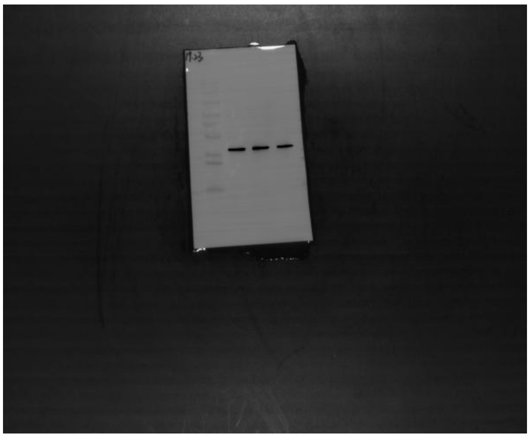

VDAC1

GAPDH

Figure 4 B

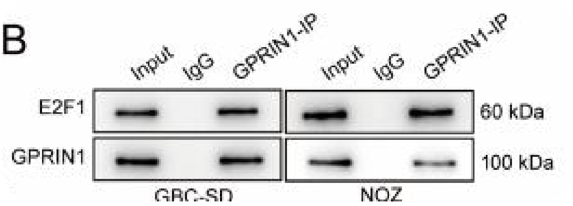

GBC-SD

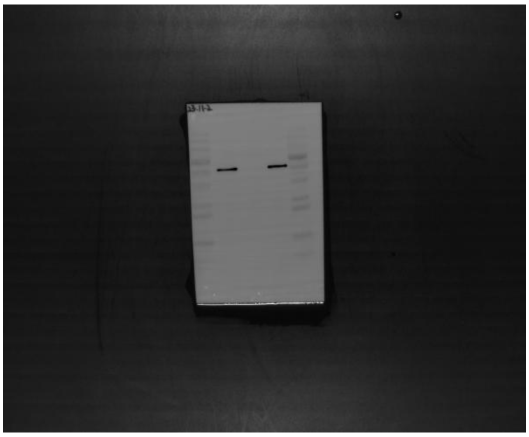

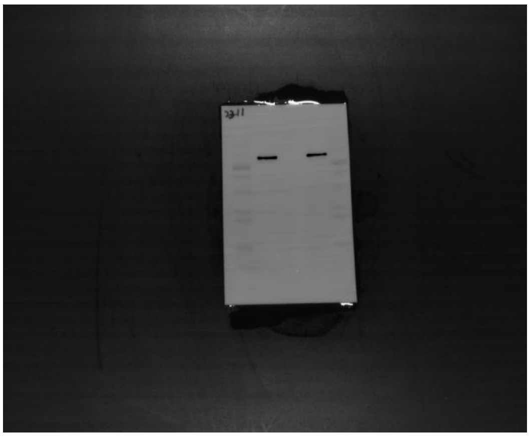

E2F1

GPRIN1

NOZ

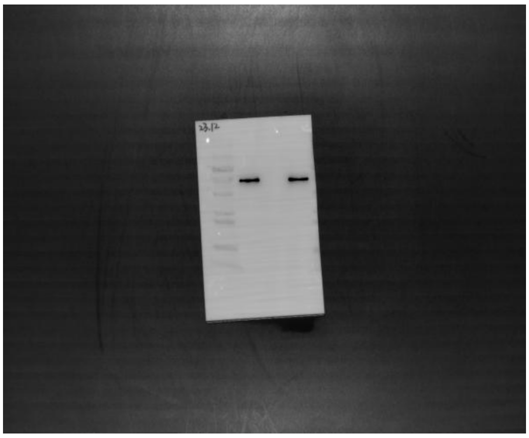

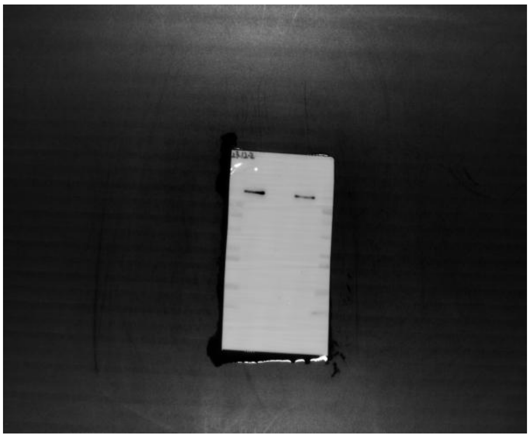

E2F1 GPRIN1

Figure 4 C

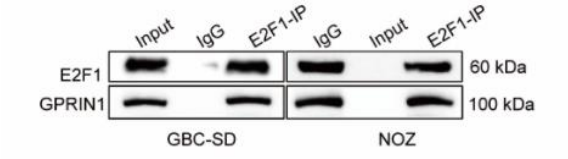

GBC-SD

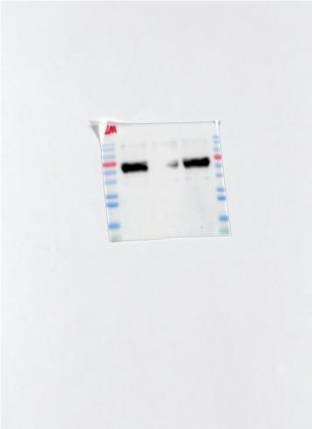

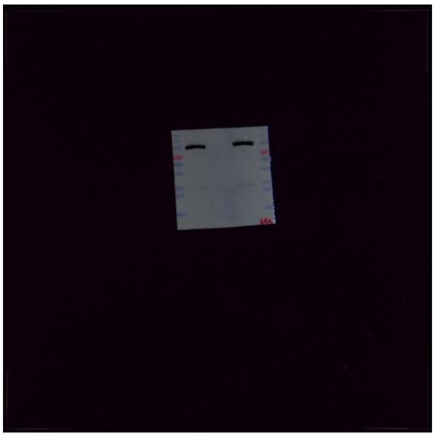

E2F1 GPRIN1

NOZ

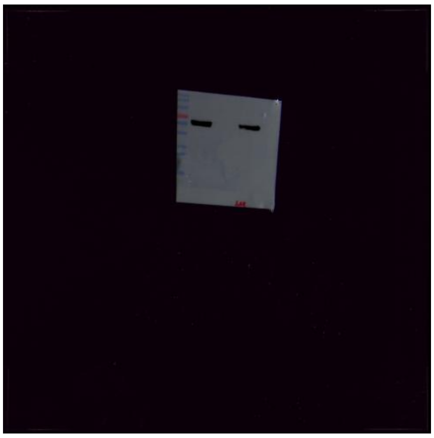

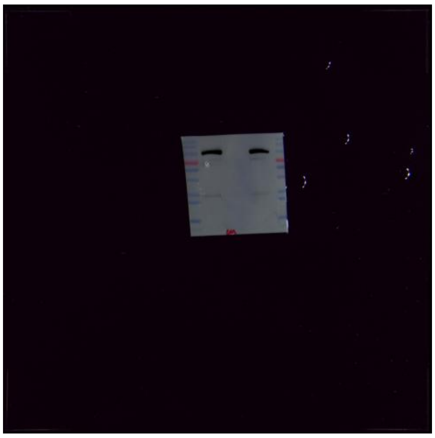

E2F1 GPRIN1

Figure 4 E

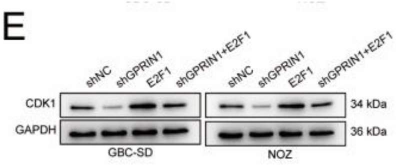

GBC-SD

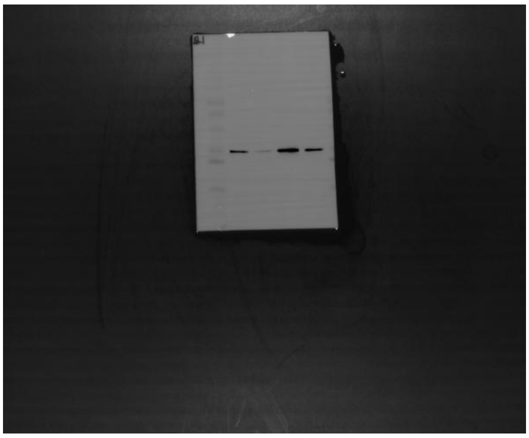

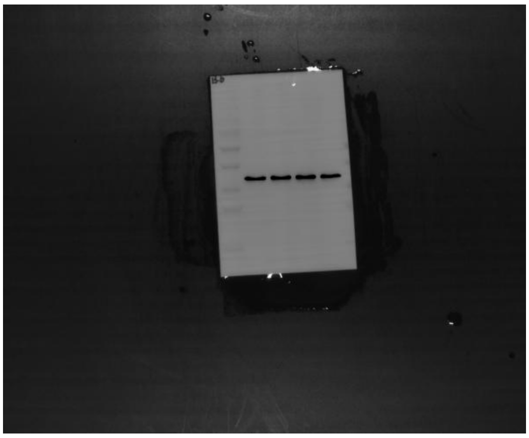

CDK1 GAPDH

NOZ

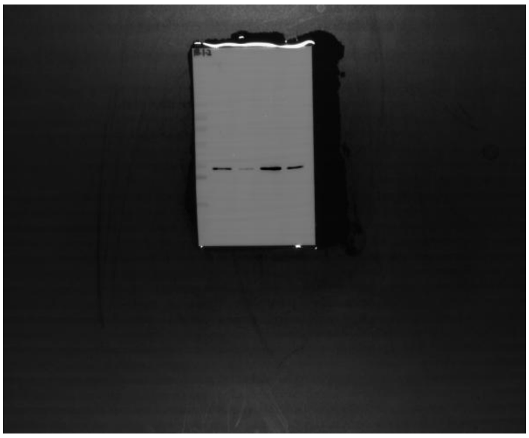

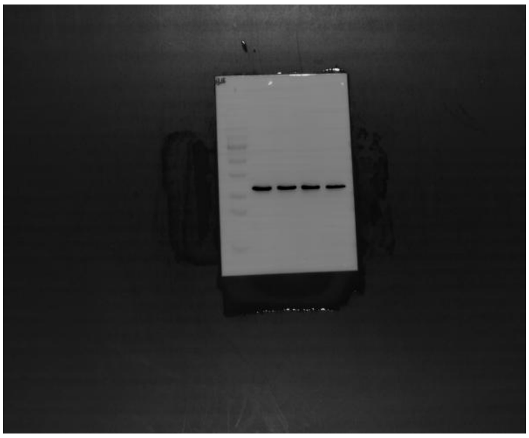

CDK1 GAPDH

Figure 4 F

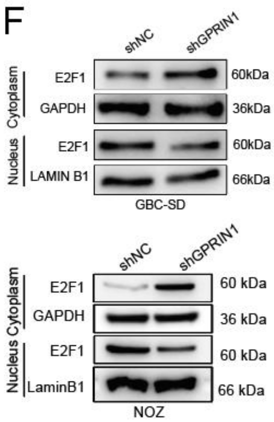

GBC-SD

Cytoplasm

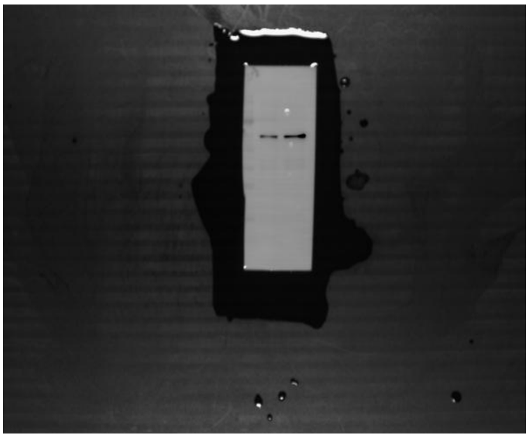

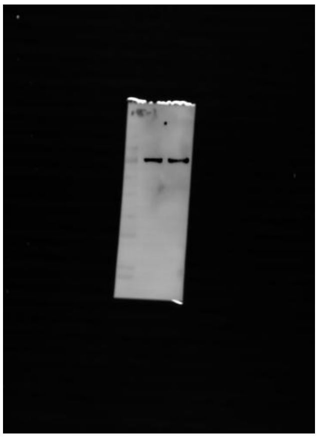

E2F1 GAPDH

Nucleus

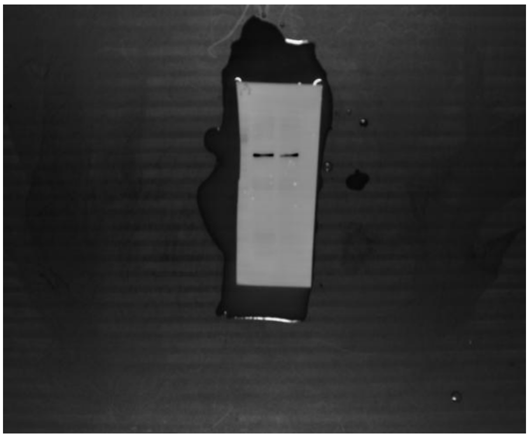

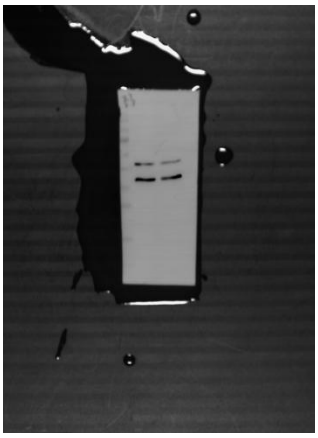

E2F1 Lamin B1

NOZ

Cytoplasm

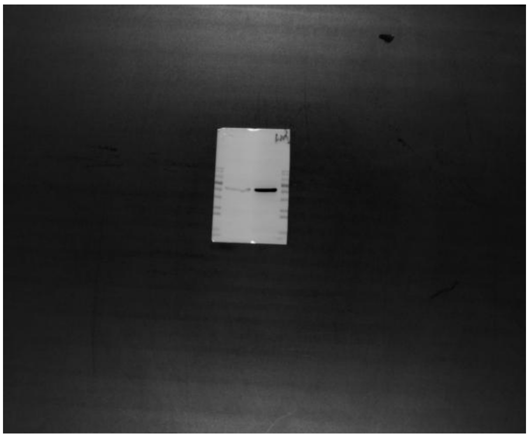

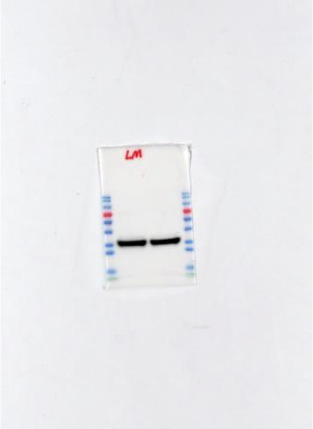

E2F1 GAPDH

Nucleus

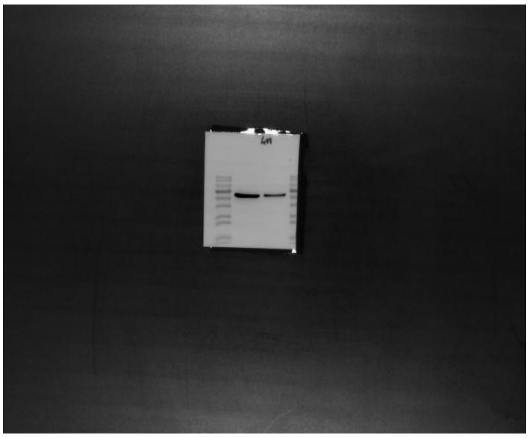

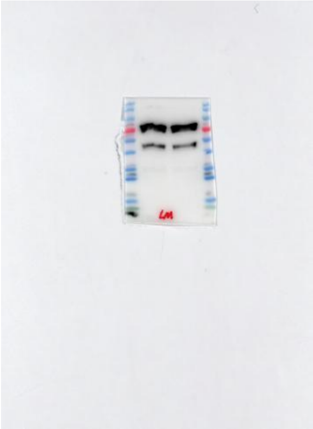

E2F1

Lamin B1

Figure 4G

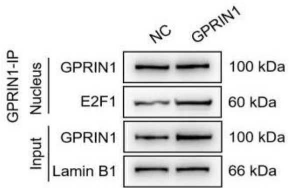

GBC-SD

IP

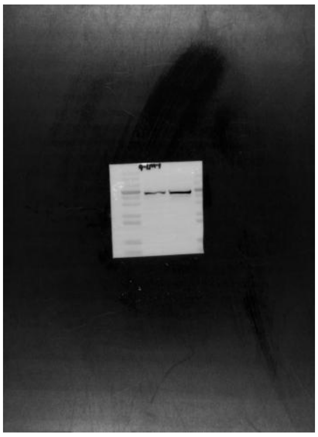

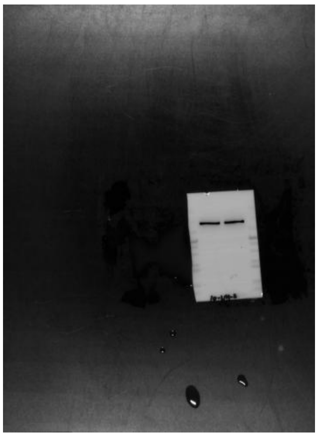

E2F1

GPRIN1

Input

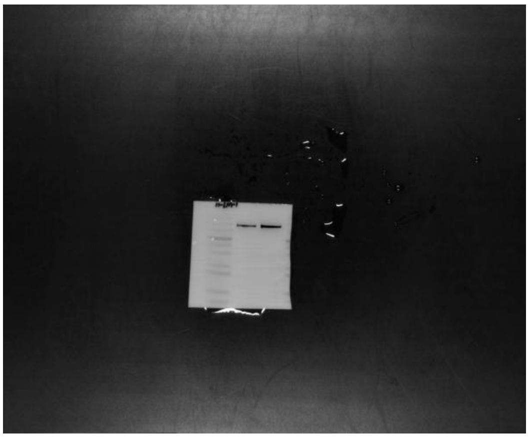

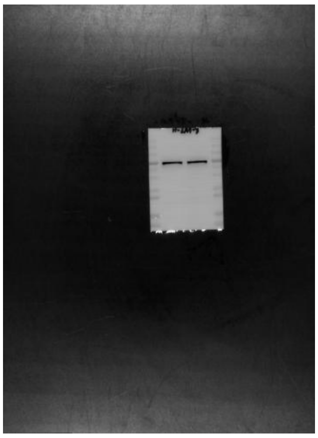

GPRIN1 Lamin B1

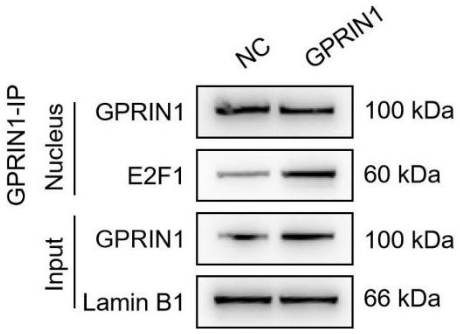

NOZ

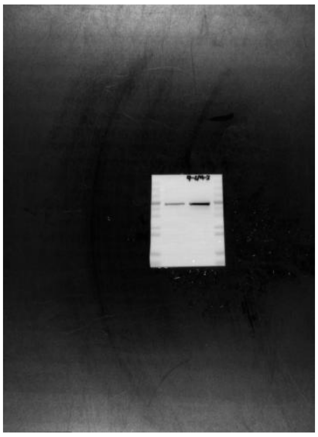

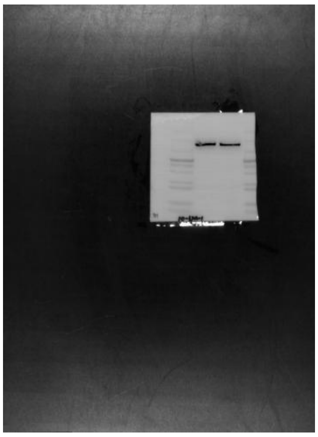

E2F1 GPRIN1

Input

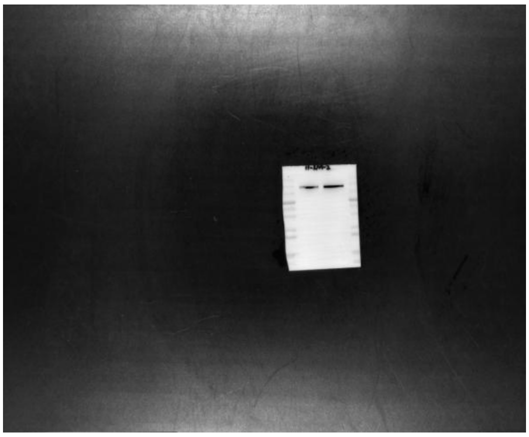

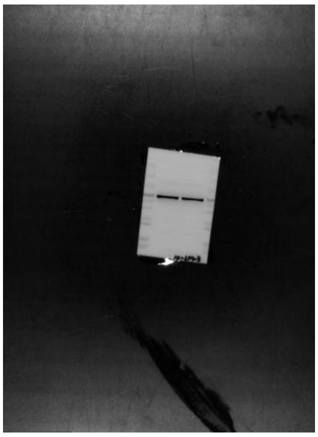

GPRIN1 Lamin B1

Figure 4H

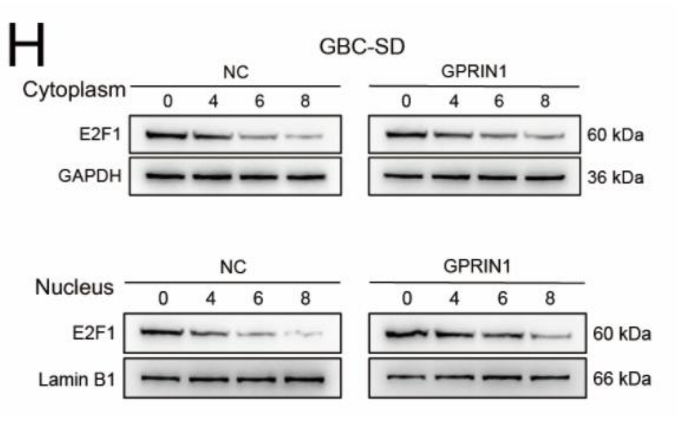

GBC-SD

Cytoplasm

NC

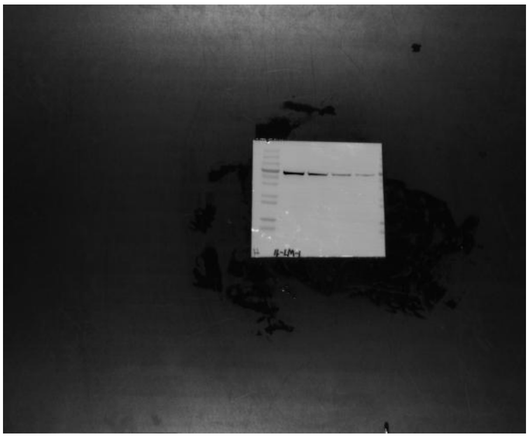
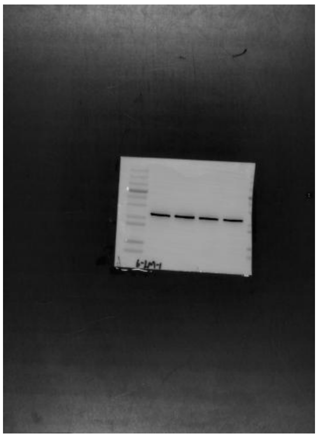

E2F1 GAPDH

GPRIN1

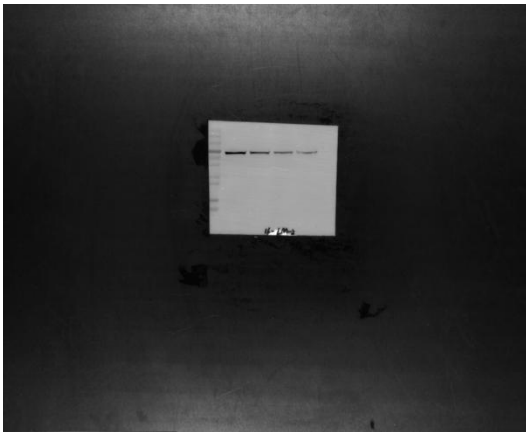
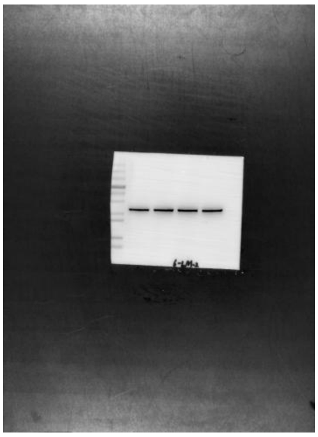

E2F1 GAPDH

Nucleu

NC

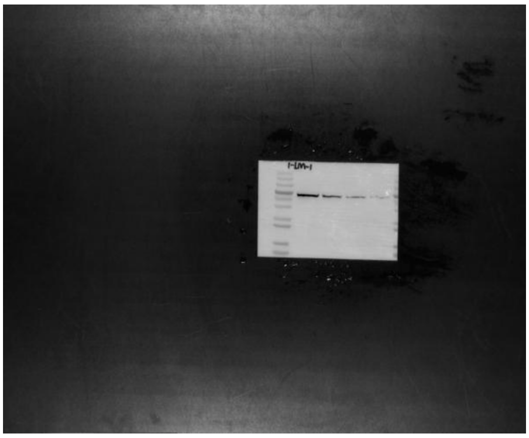

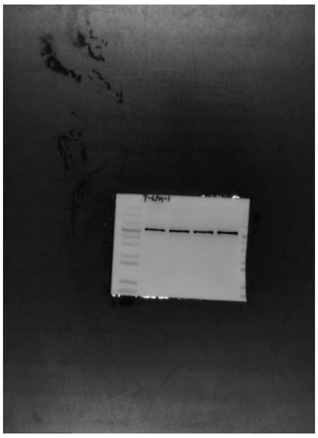

E2F1 LaminB1

GPRIN1

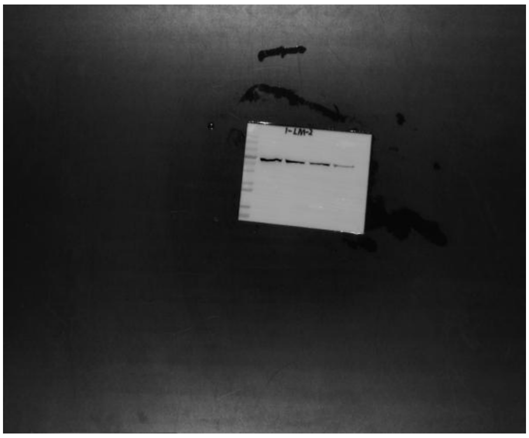

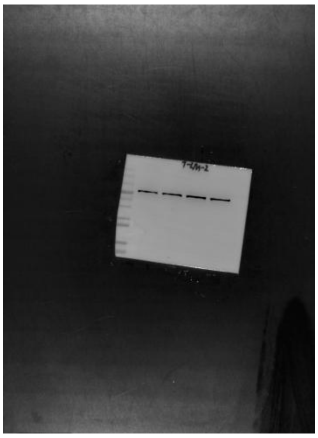

E2F1 LaminB1

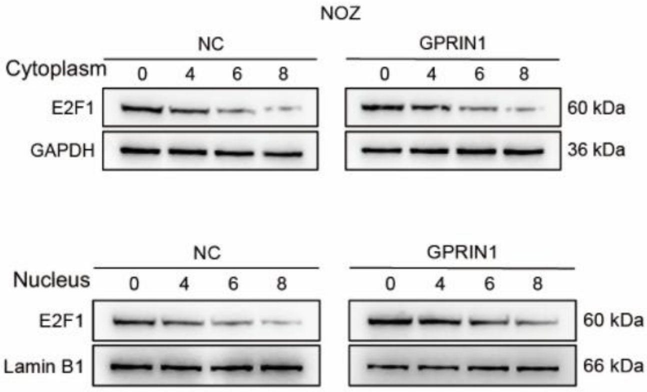

NOZ

NC

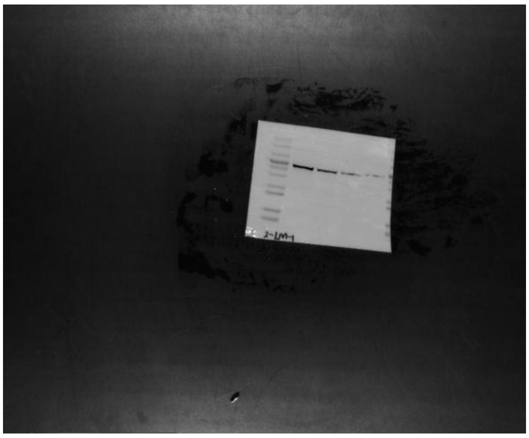

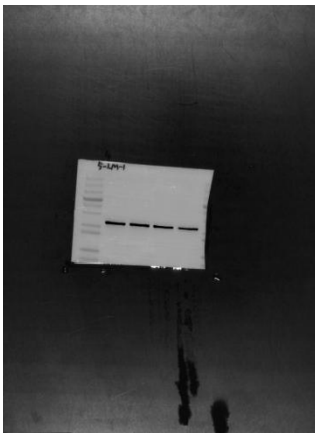

E2F1 GAPDH

GPRIN1

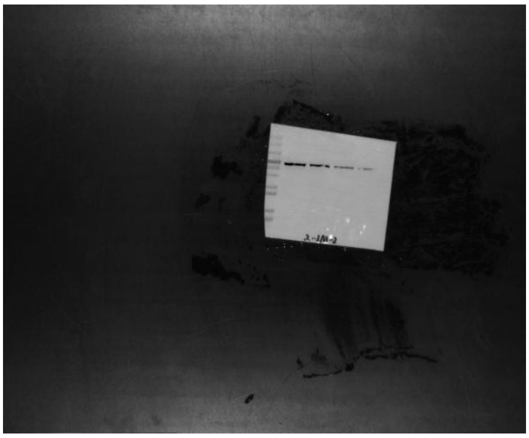

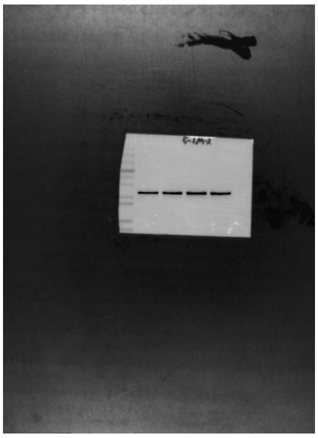

E2F1 GAPDH

Nucleu

NC

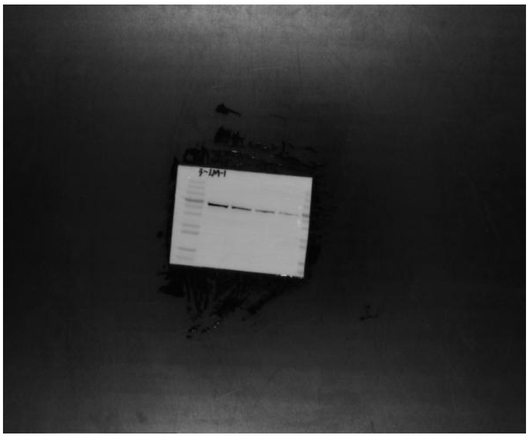

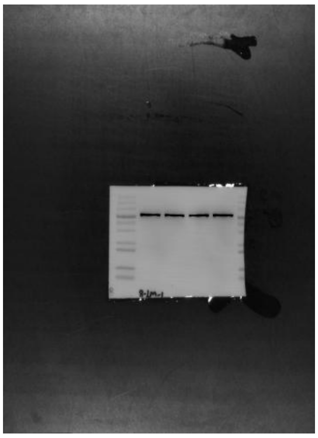

E2F1 LaminB1

GPRIN1

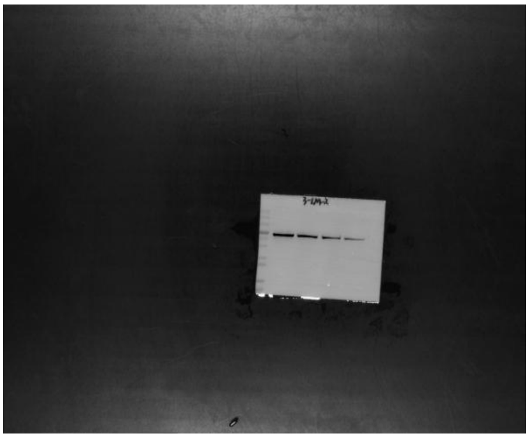

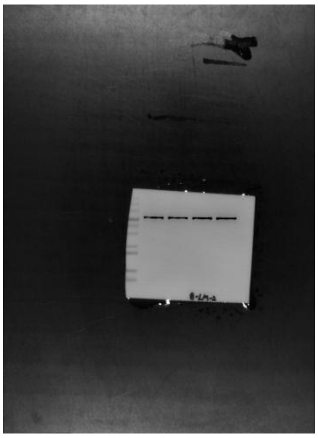

E2F1 LaminB1

Figure 5B

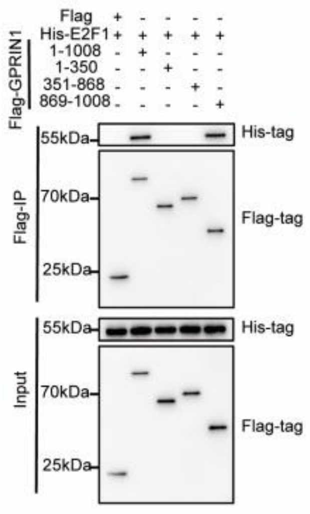

IP

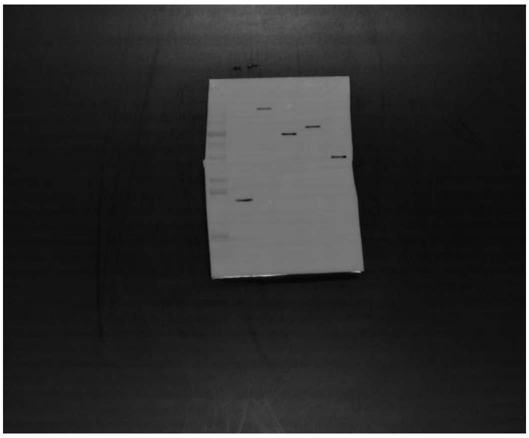

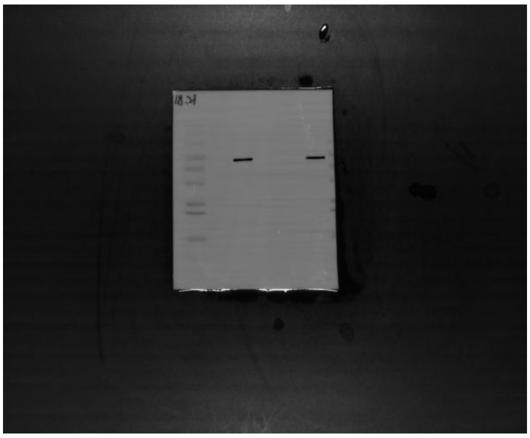

Flag-tag his-tag

Input

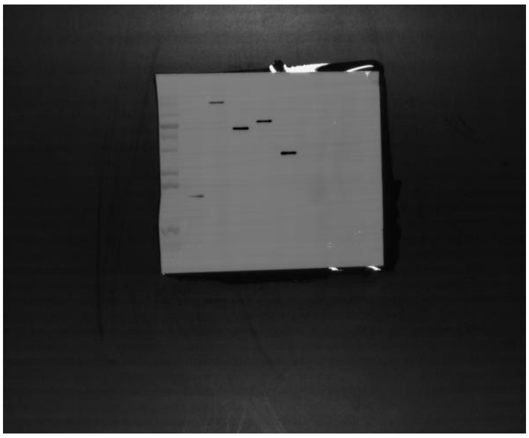

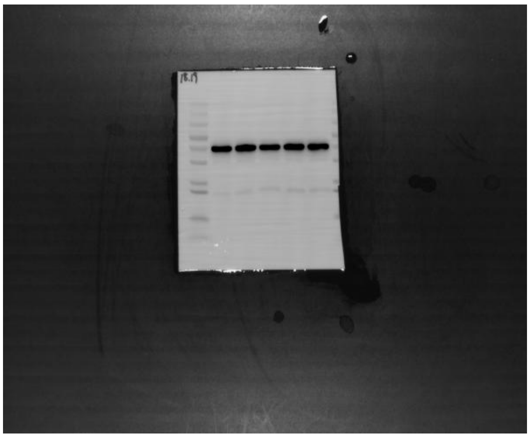

Flag-tag his-tag

Figure 5C

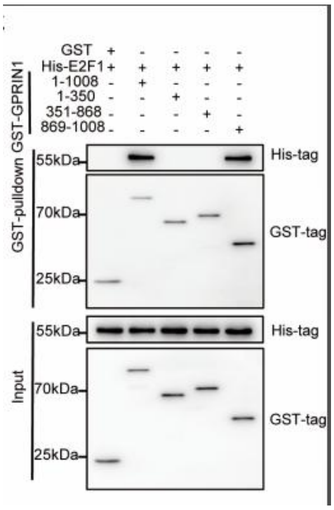

GST pulldown

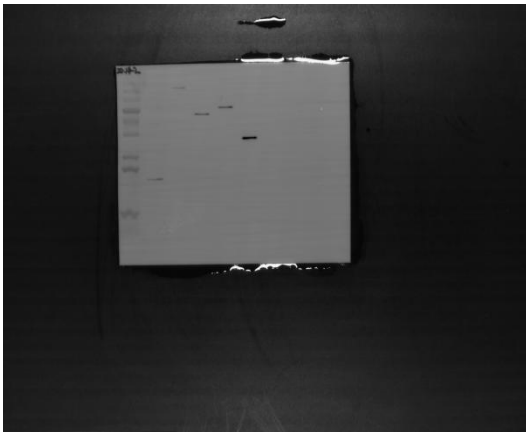

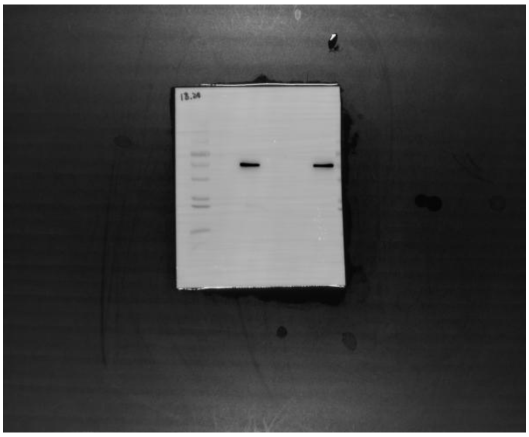

GST Tag His-Tag

Input

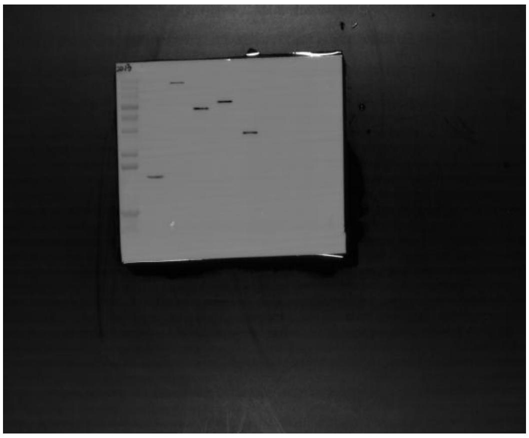 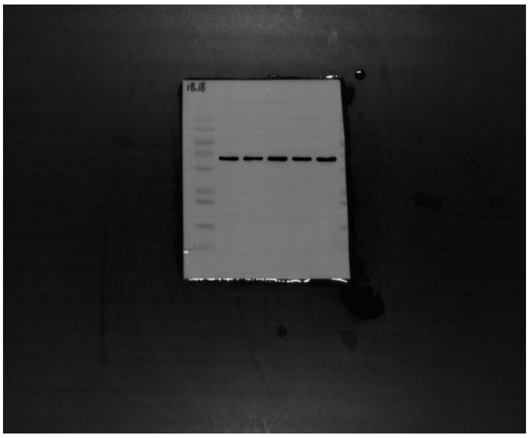

GST Tag His-Tag

Figure 5D

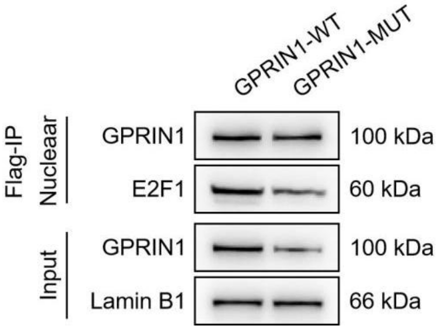

GBC-SD

Flag-IP

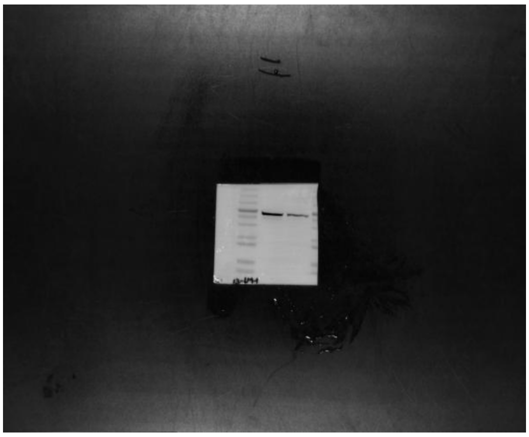 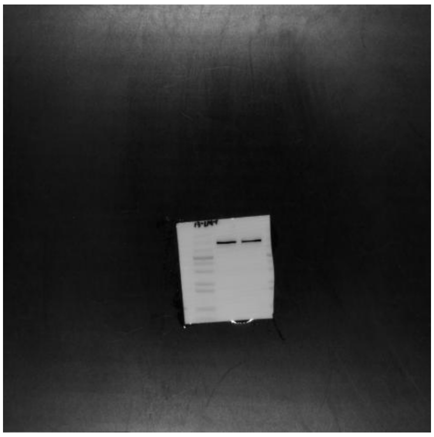

E2F1 GPRIN1

Input

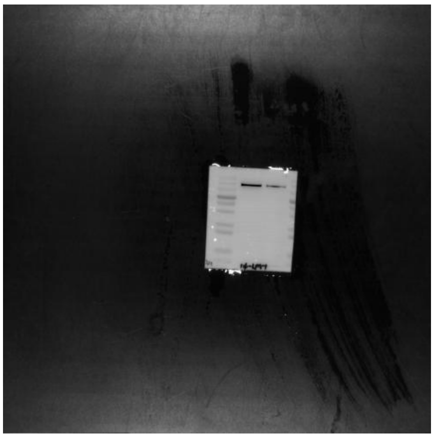

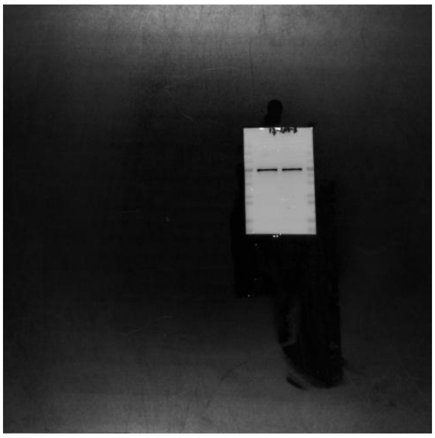

GPRIN1

LaminB1

NOZ

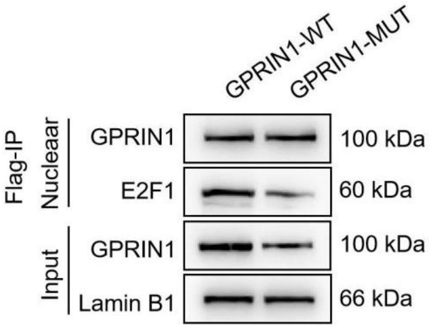

Flag-IP

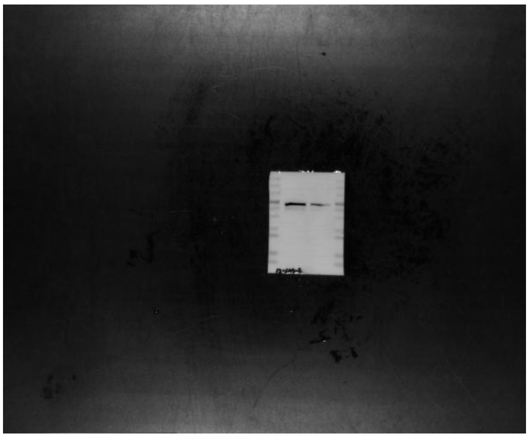

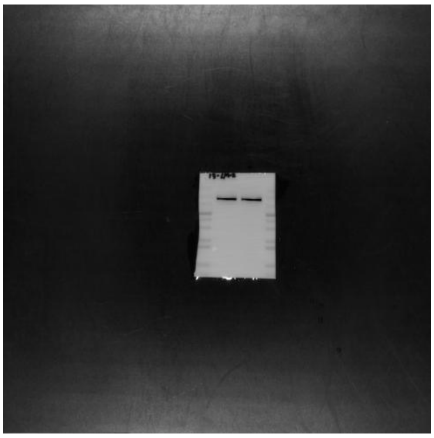

E2F1

GPRIN1

Input

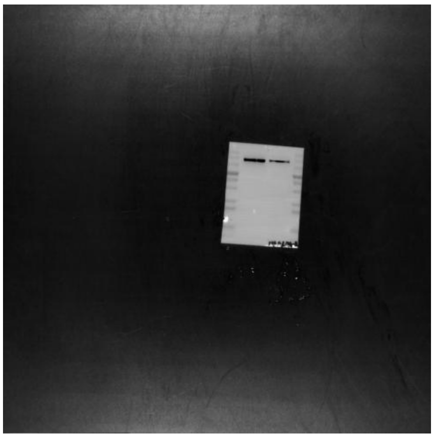

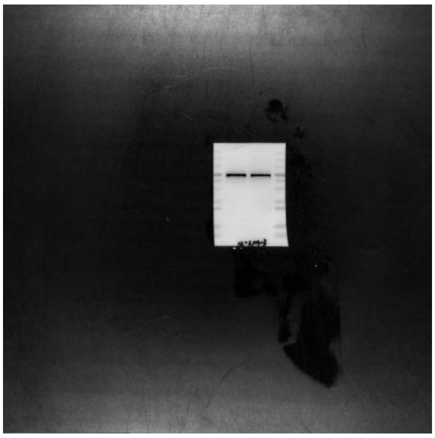

GPRIN1

LaminB1

Figure 5G

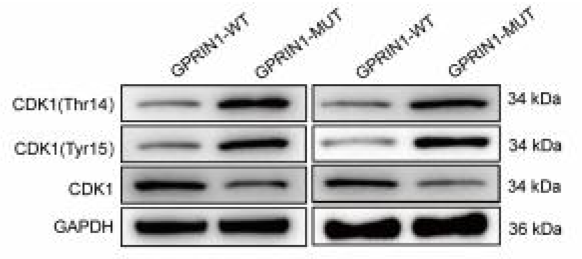

GBC-SD

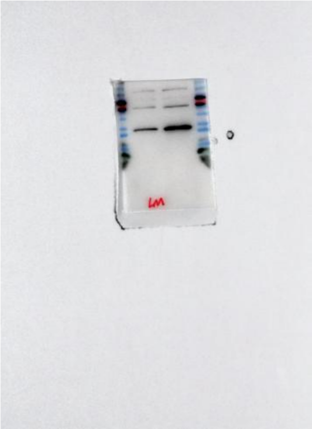

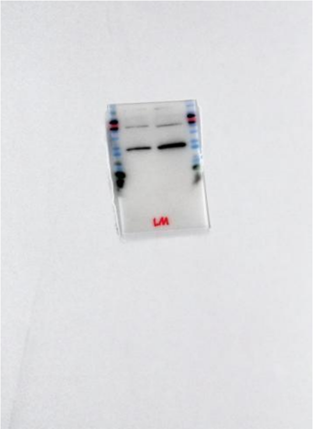

CDK1(Thr14)

CDK1(Tyr15)

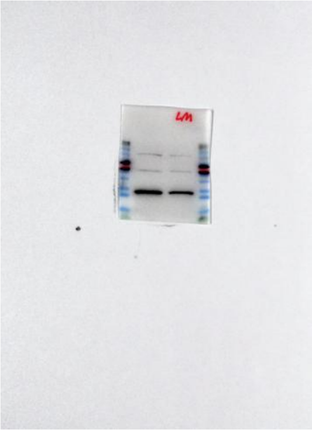

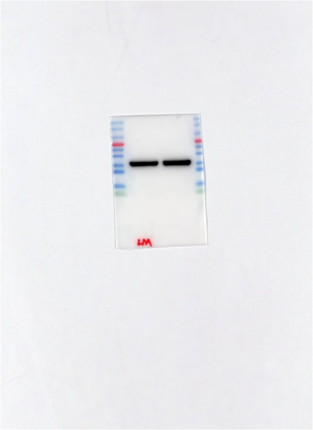

CDK1 GAPDH

NOZ

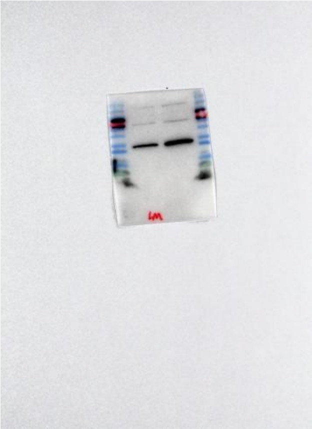

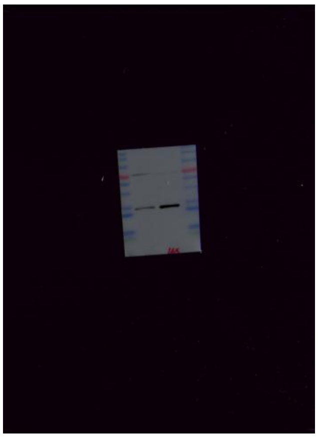

CDK1(Thr14) CDK1(Tyr15)

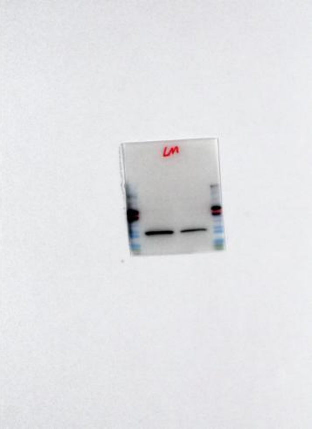

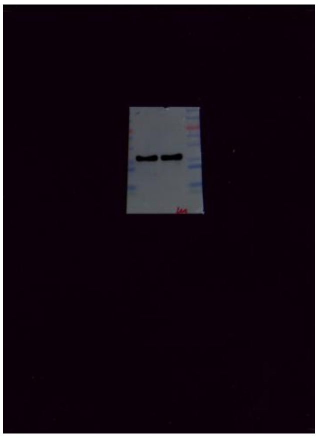

CDK1 GAPDH

Figure 5I

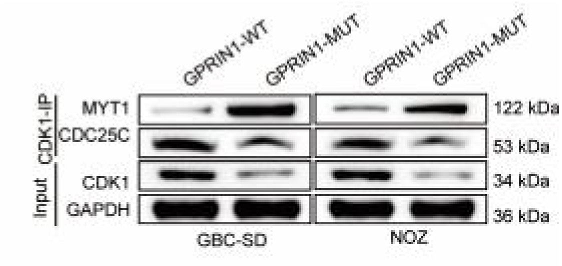

GBC-SD

CDK1-IP

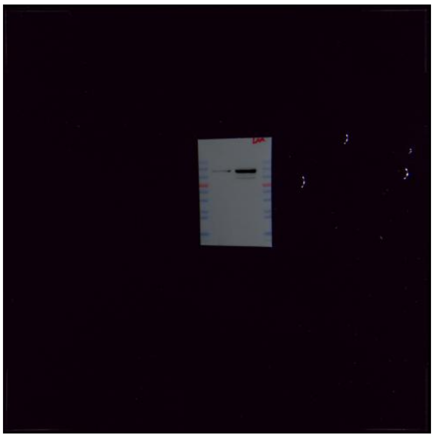

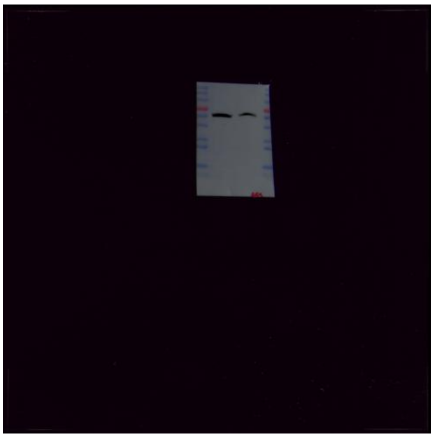

MYT1

CDC25C

INPUT

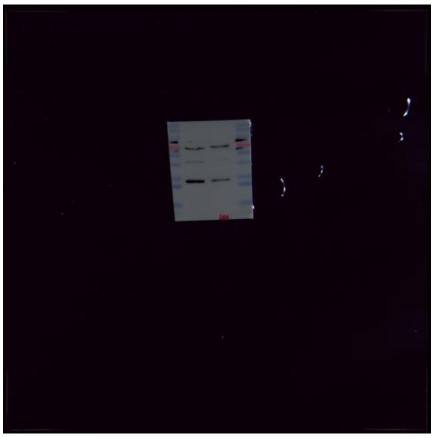

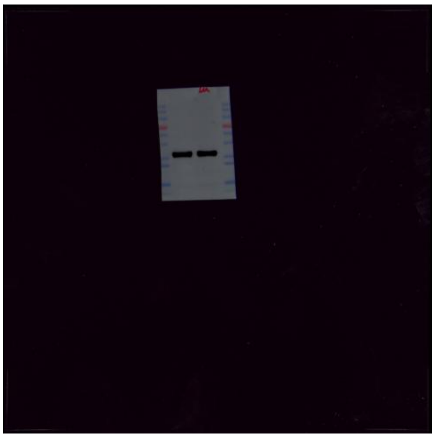

CDK1

GAPDH

NOZ

CDK1-IP

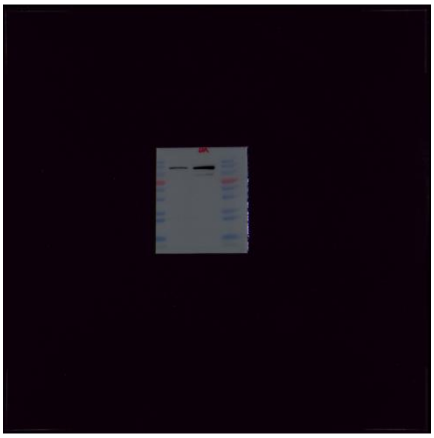

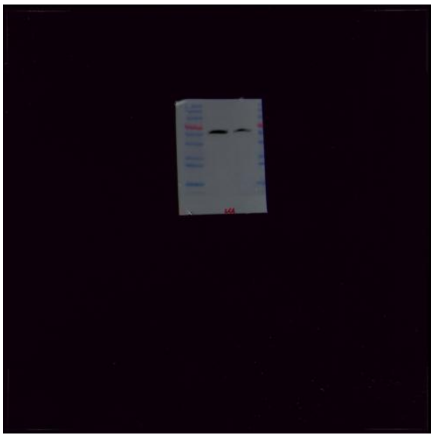

MYT1

CDC25C

INPUT

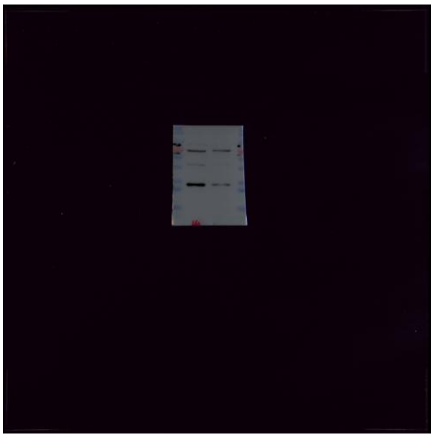

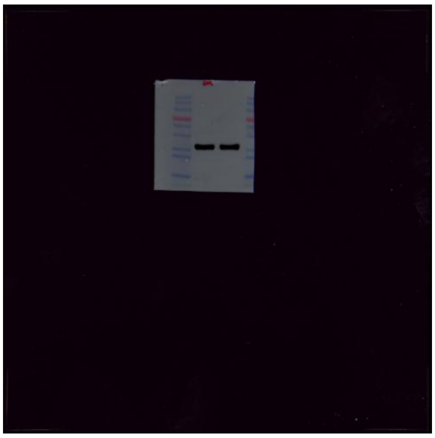

CDK1

GAPDH

Figure 6B

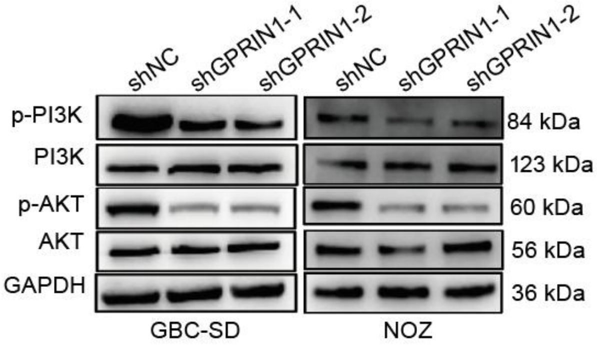

GBC-SD

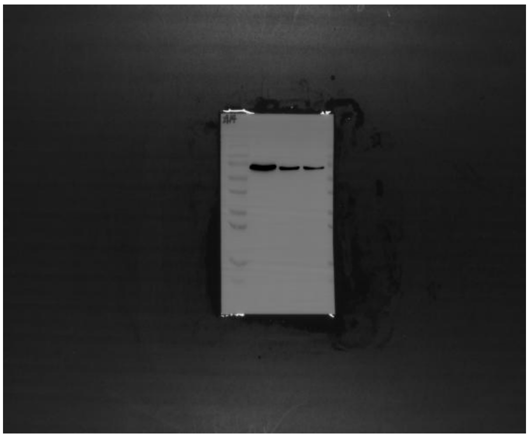

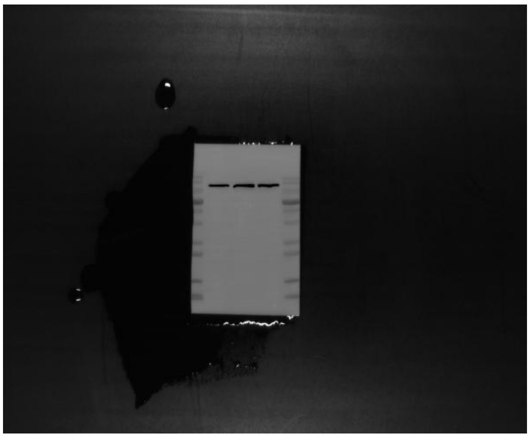

p-PI3K

PI3K

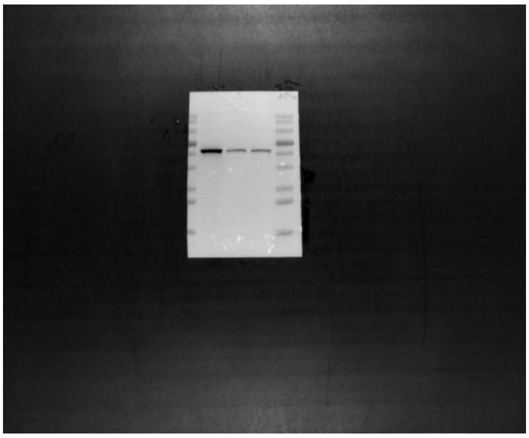

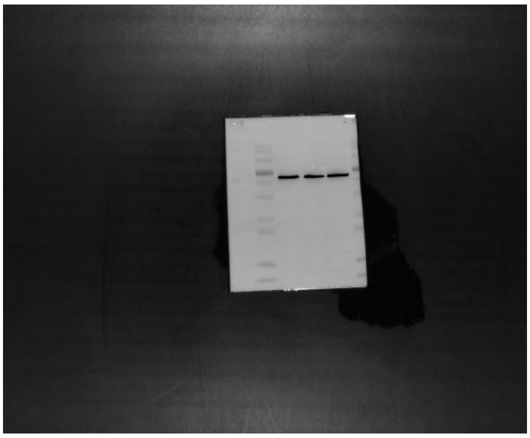

p-AKT

AKT

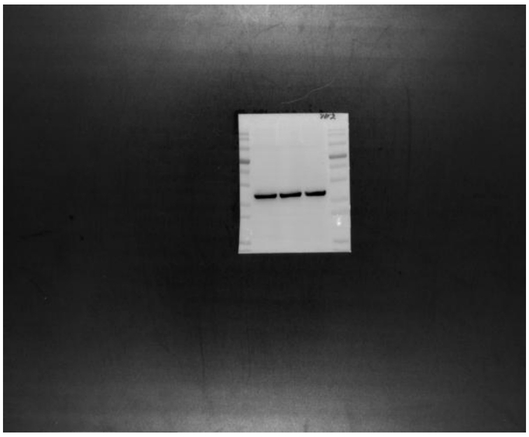

GAPDH

NOZ

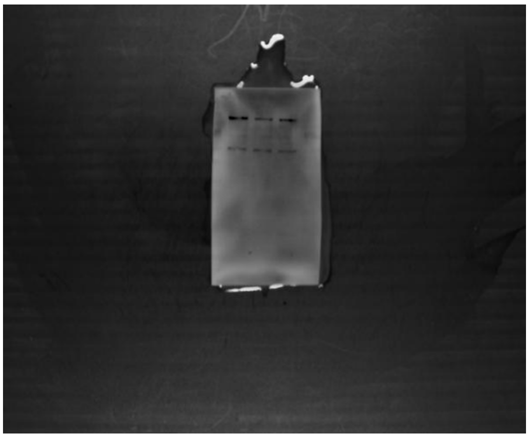

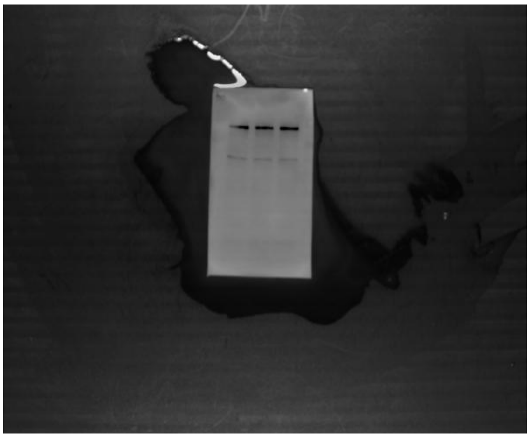

p-PI3K

PI3K

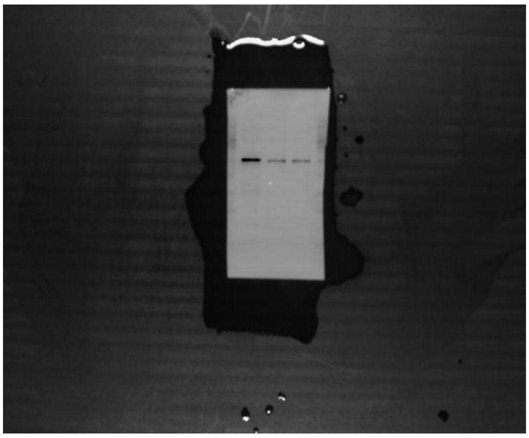

p-AKT

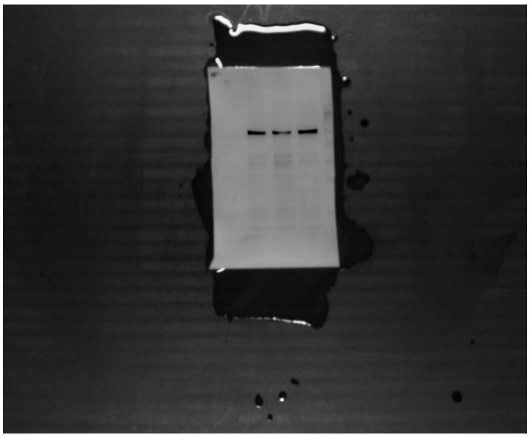

AKT

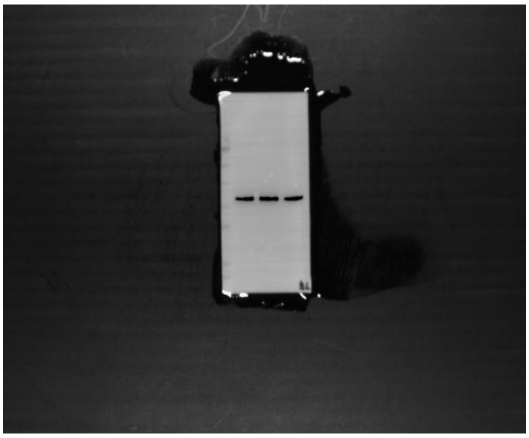

GAPDH

Figure 6C

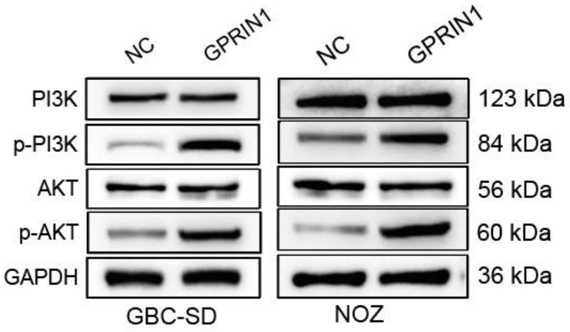

GBC-SD

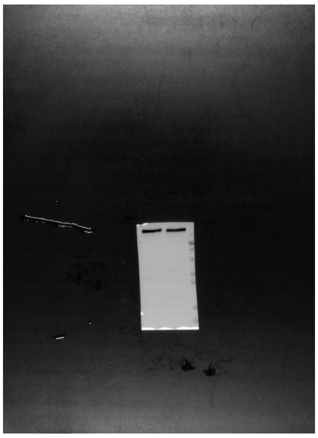
PI3K

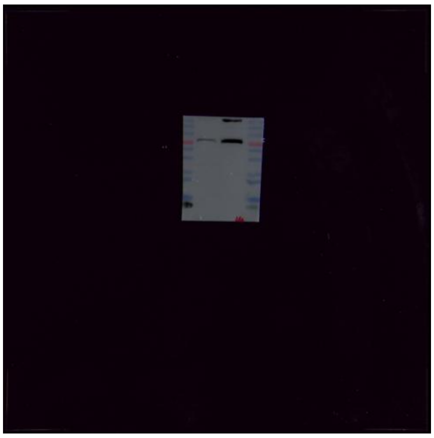
P-PI3K

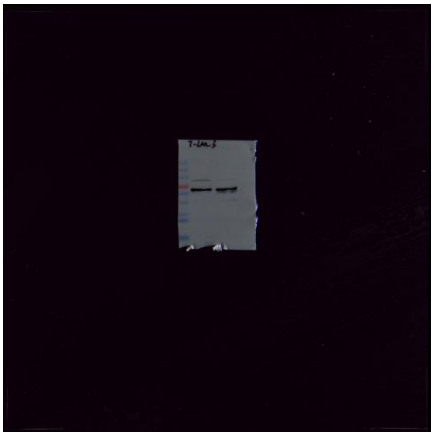
AKT

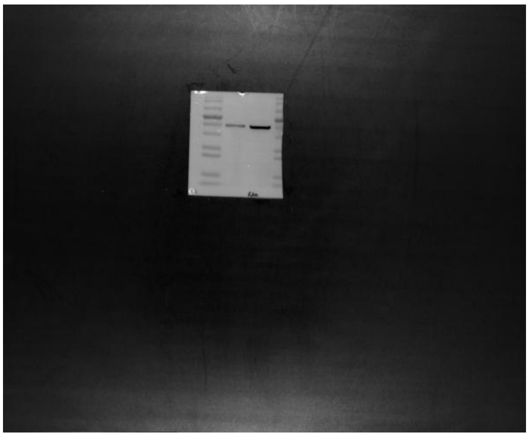
P-AKT

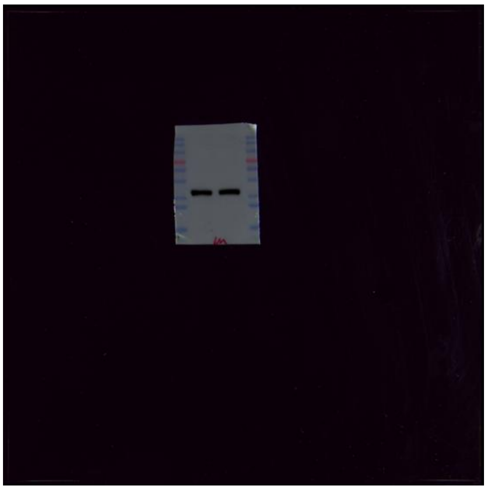
GAPDH

NOZ

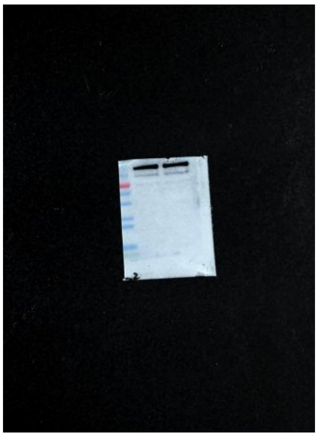

PI3K

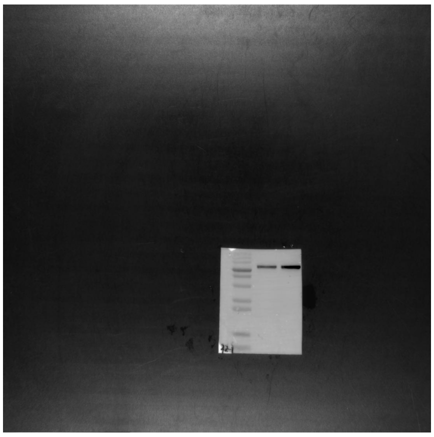

P-PI3K

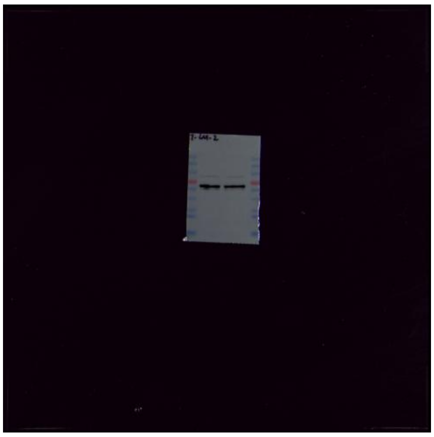

AKT

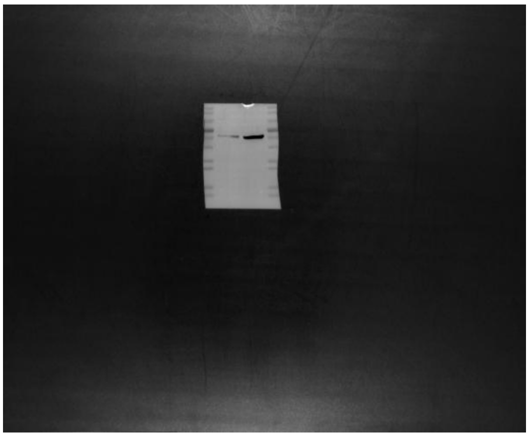

P-AKT

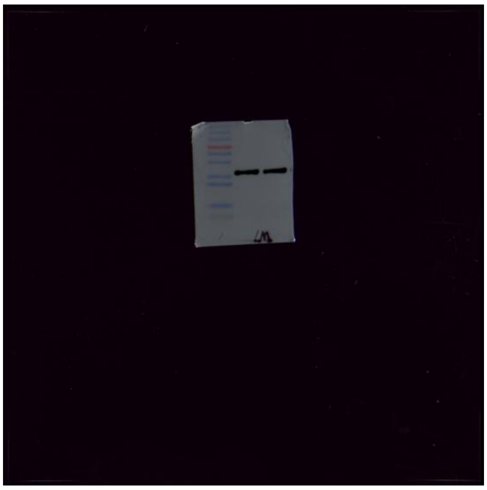

GAPDH

Figure 6E

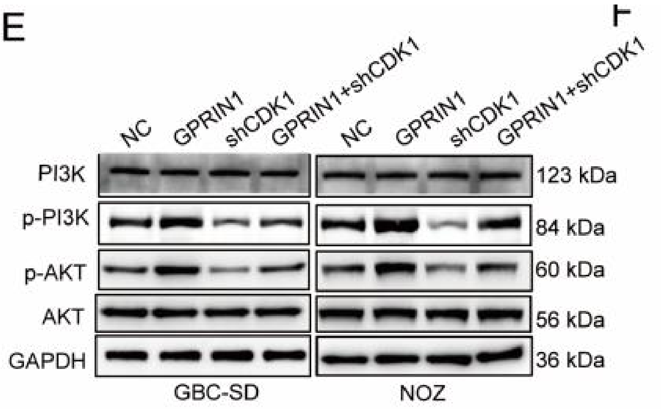

GBC-SD

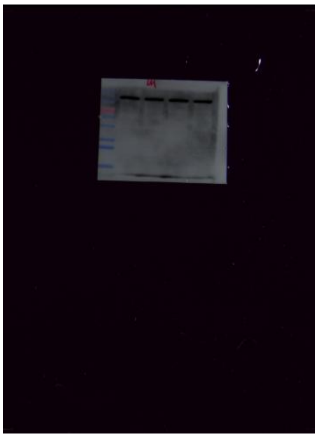

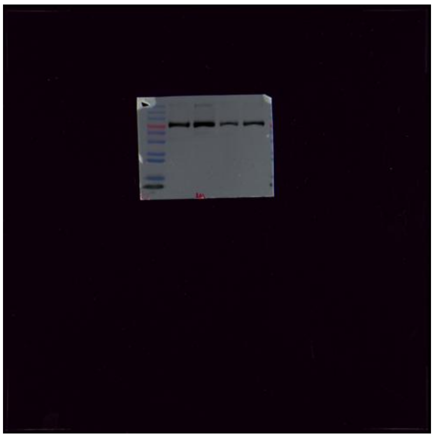

PI3K

P-PI3K

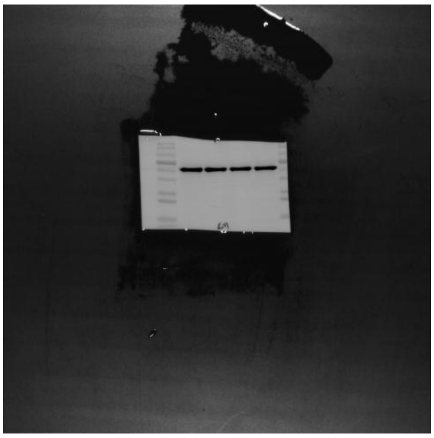

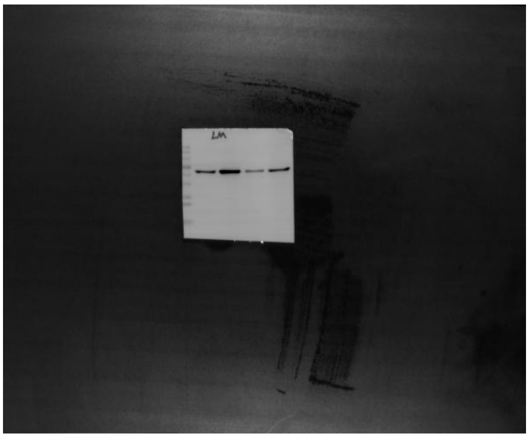

AKT

P-AKT

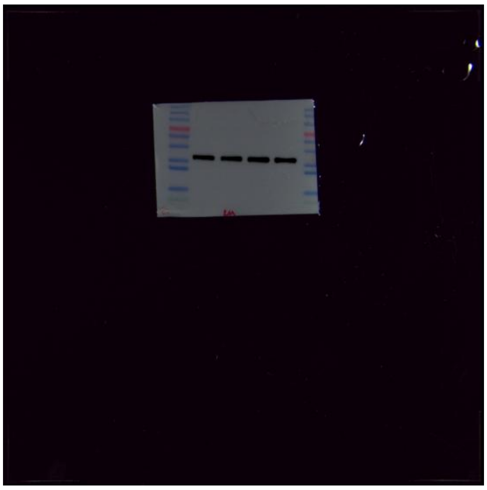

GAPDH

NOZ

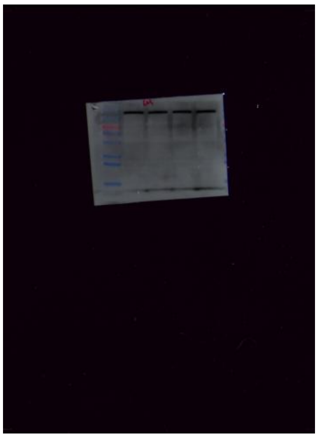

PI3K

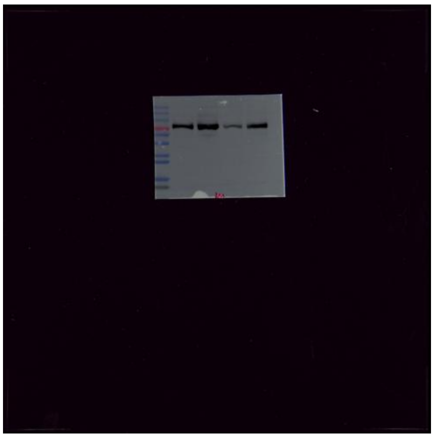

P-PI3K

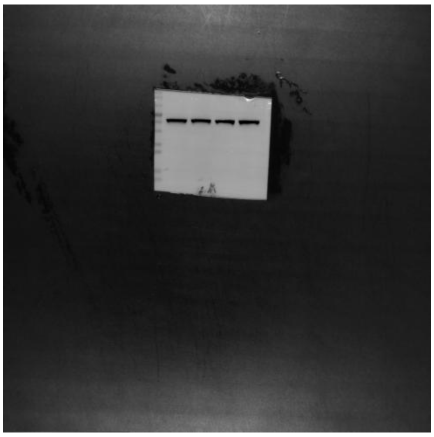

AKT

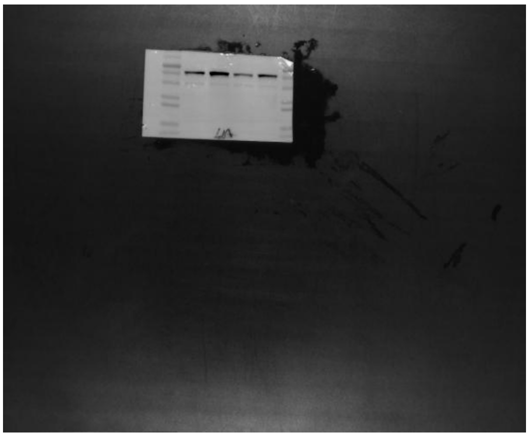

P-AKT

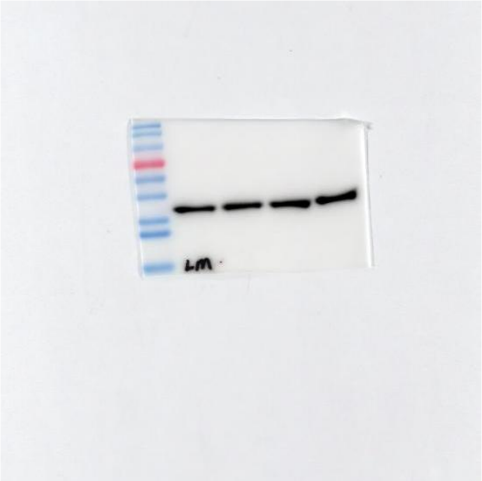

GAPDH

Figure 6G

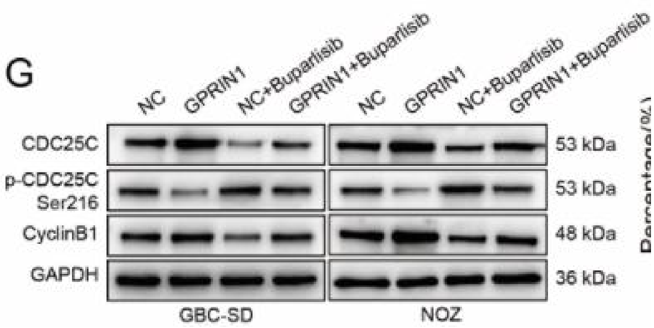

GBC-SD

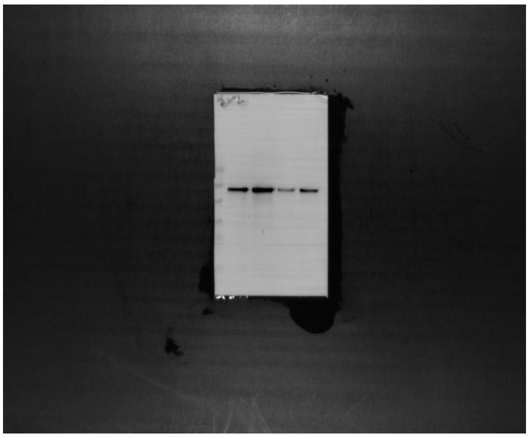

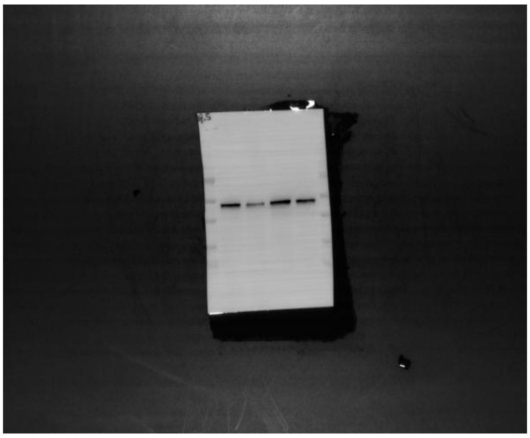

CDC25C

P-CDC25C

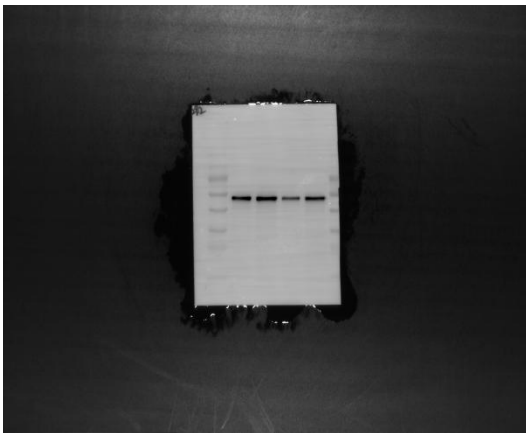

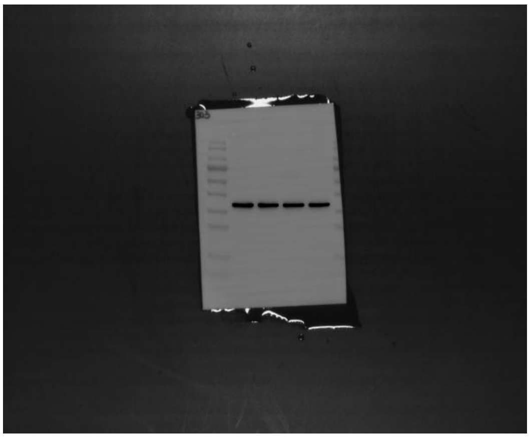

CyclinB1

GAPDH

NOZ

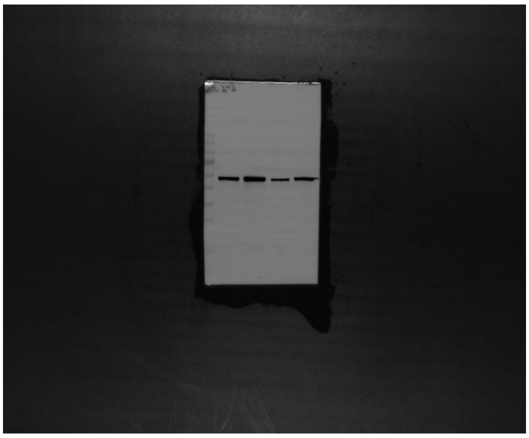

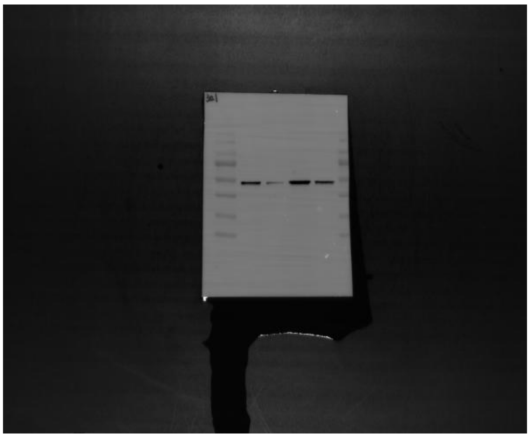

CDC25C

P-CDC25C

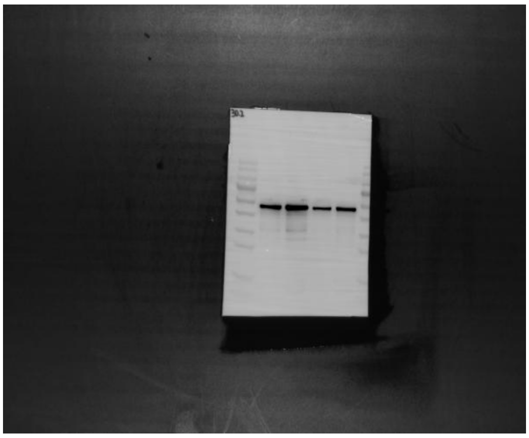

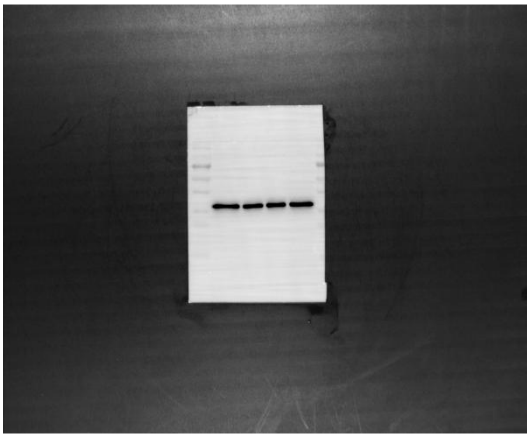

CyclinB1

GAPDH

Figure 6J

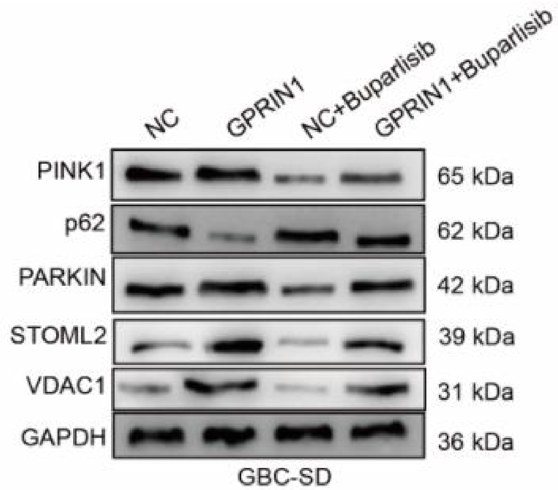

GBC-SD

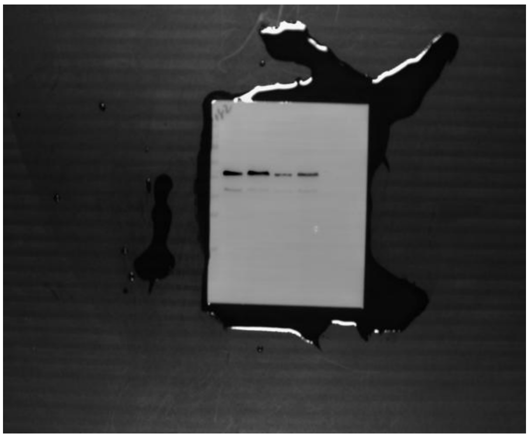

PINK1

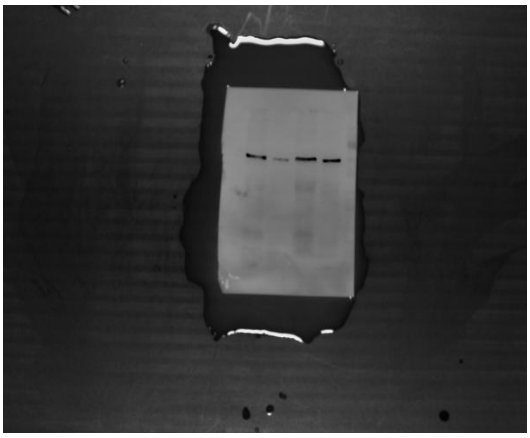

P62

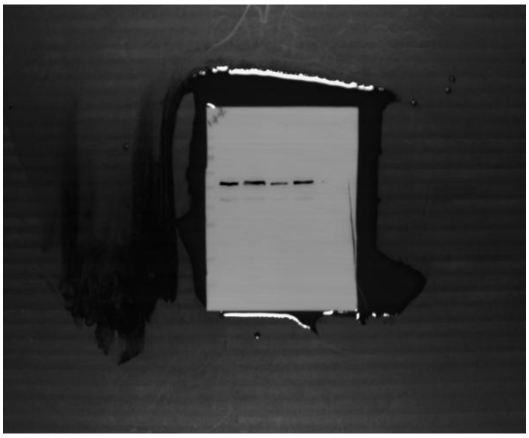

PARKIN

STOML2

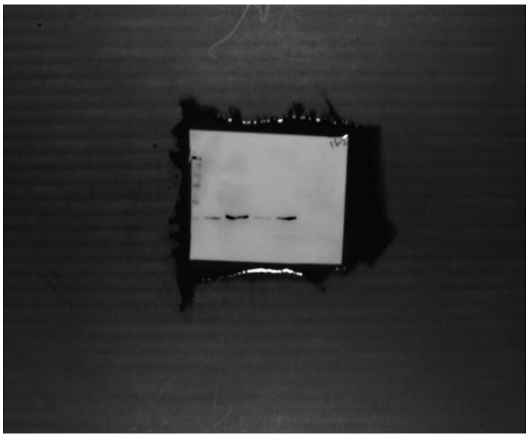

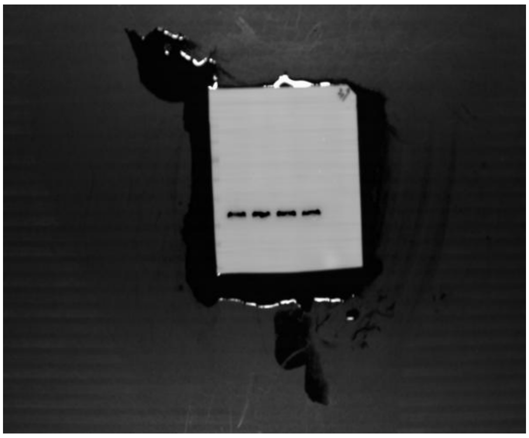

VDAC1 GAPDH

NOZ

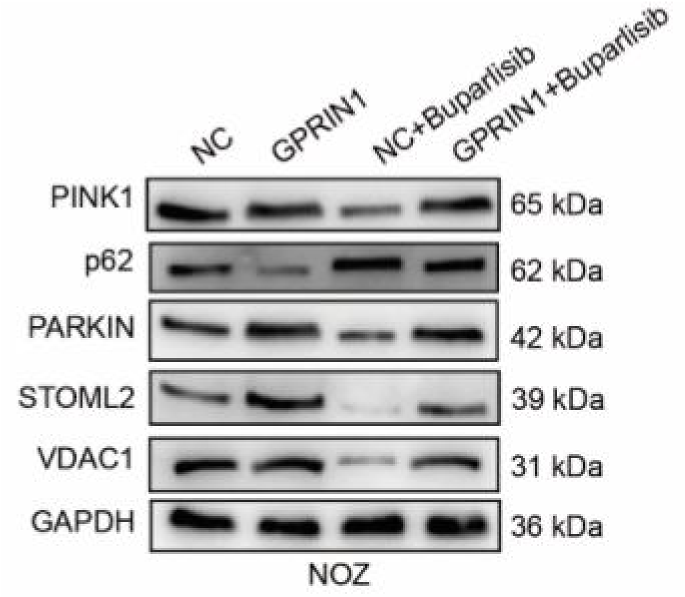

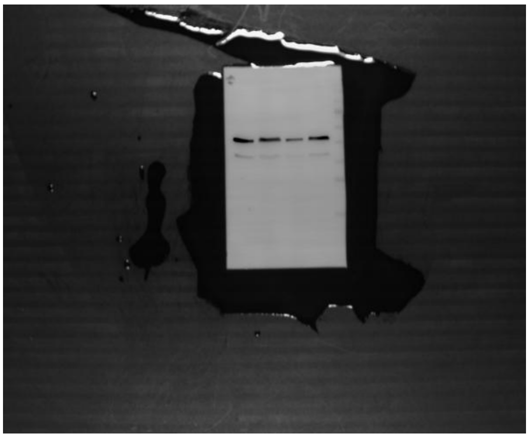

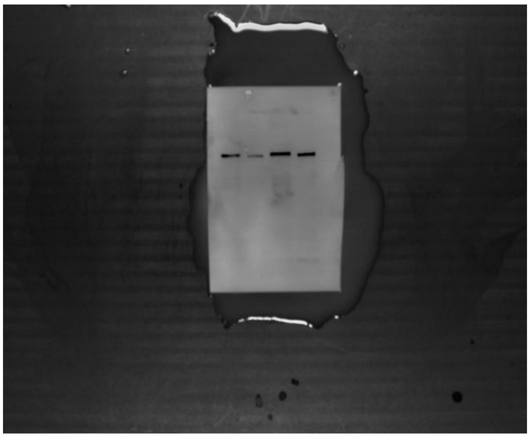

PINK1 P62

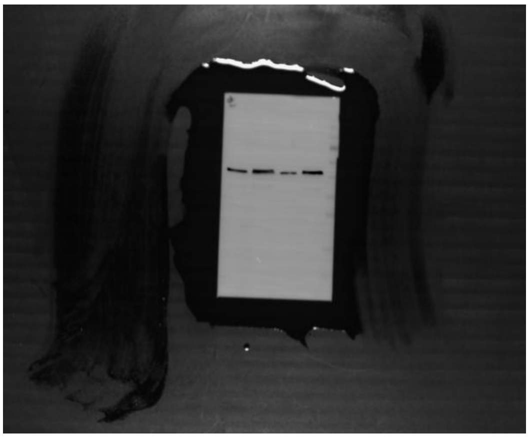

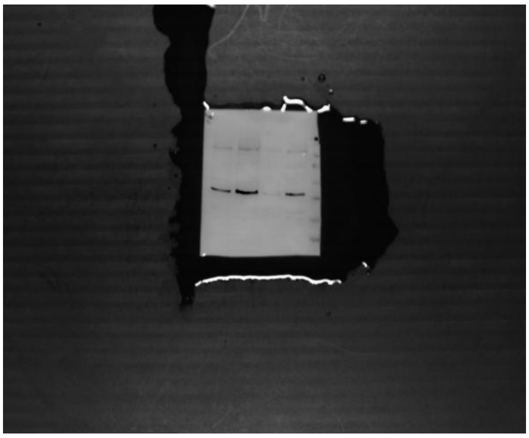

PARKIN

STOML2

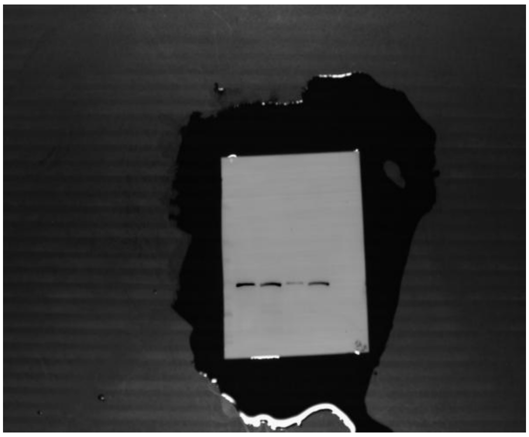

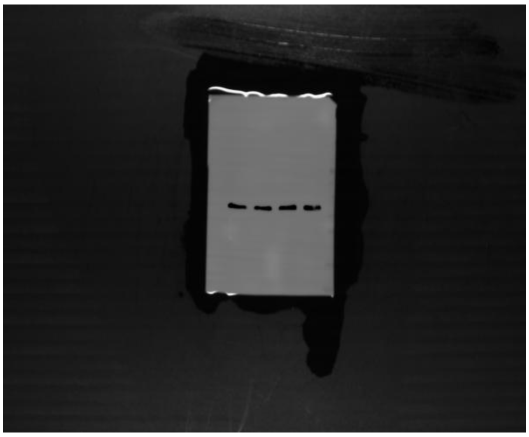

VDAC1

GAPDH

Figure S1A

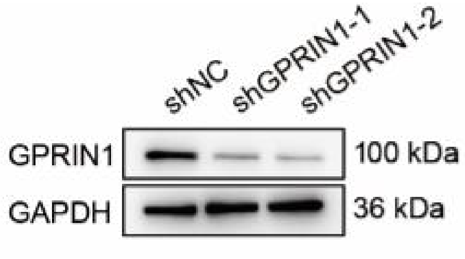

GBC-SD

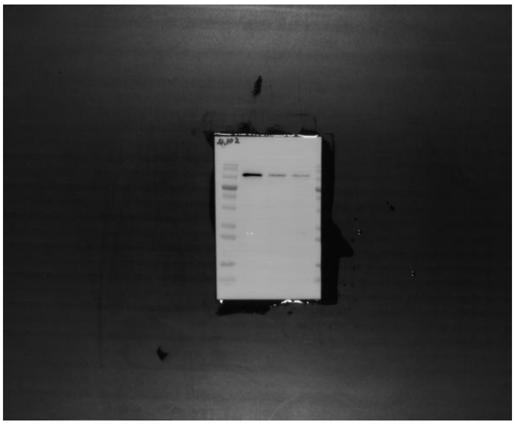

GPRIN1

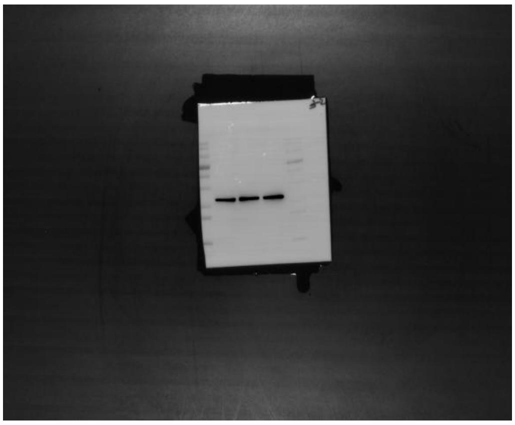

GAPDH

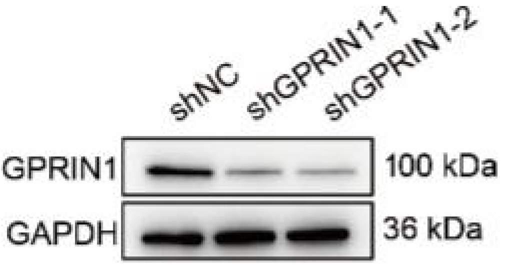

NOZ

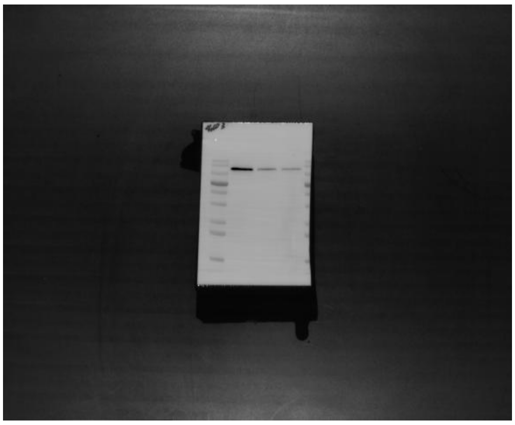

GPRIN1

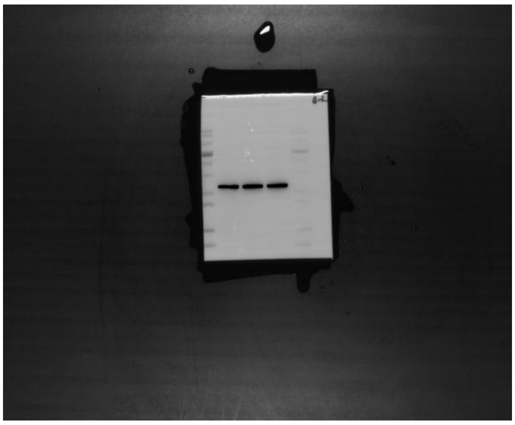

GAPDH

Figure S1B

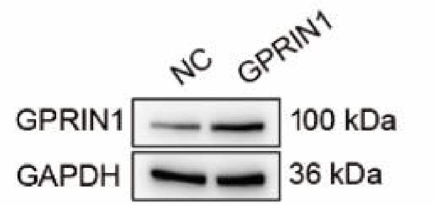

GBC-SD

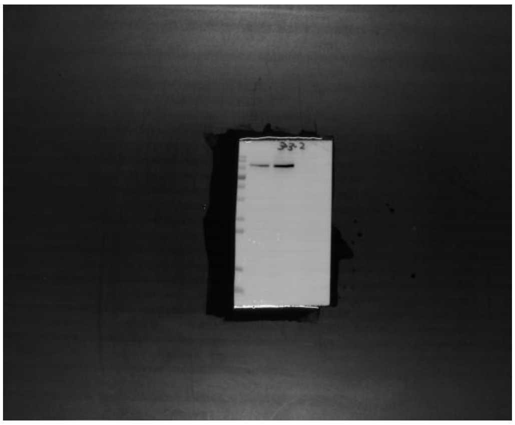

GPRIN1

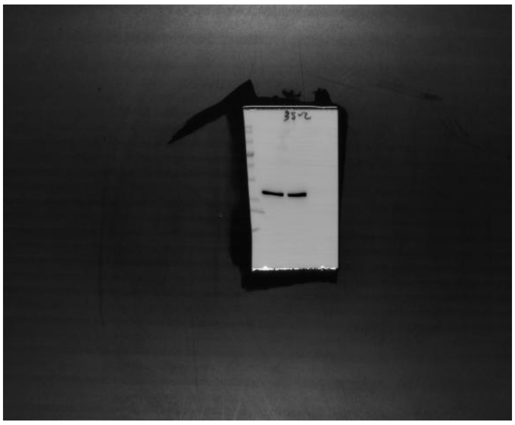

GAPDH

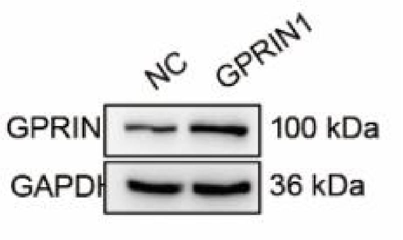

NOX

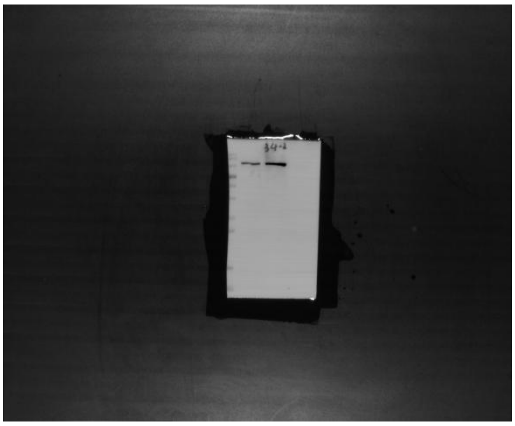

GPRIN1

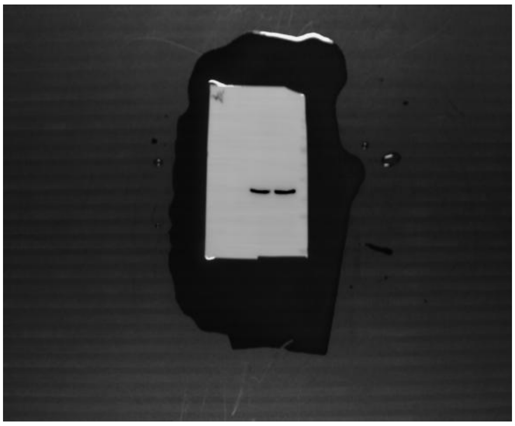

GAPDH

Figure S1H

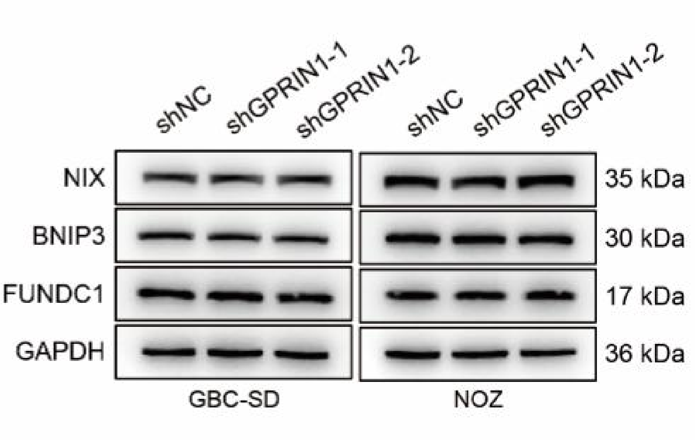

GBC-SD

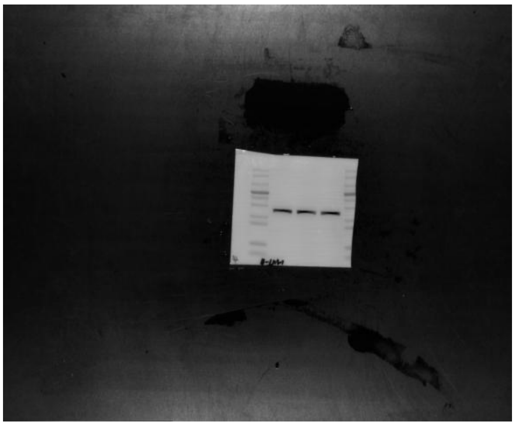

NIX

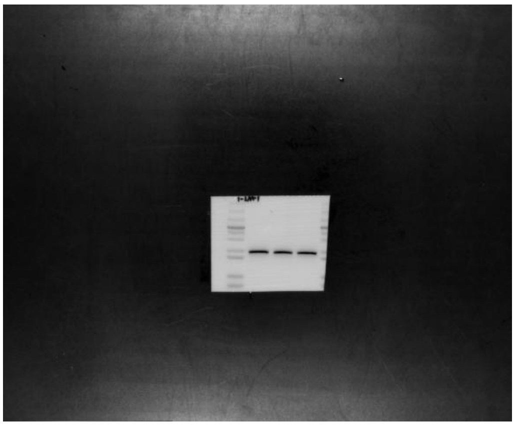

BNIP3

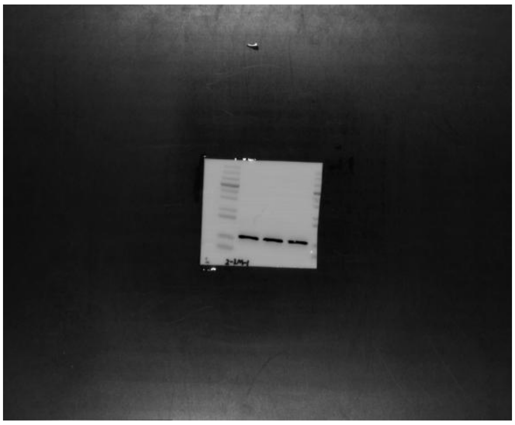

FUNDC1

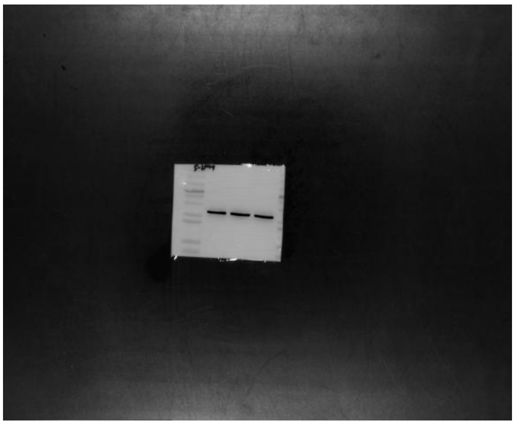

GAPDH

NOZ

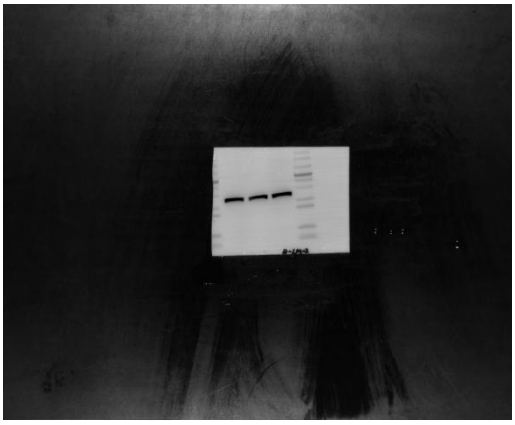

NIX

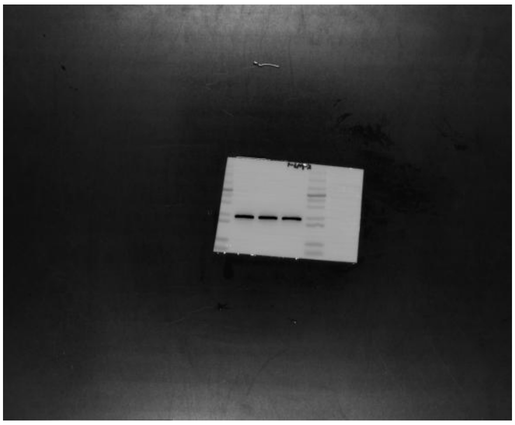

BNIP3

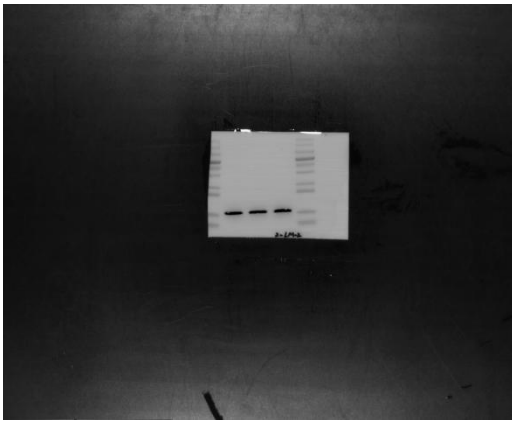

FUNDC1

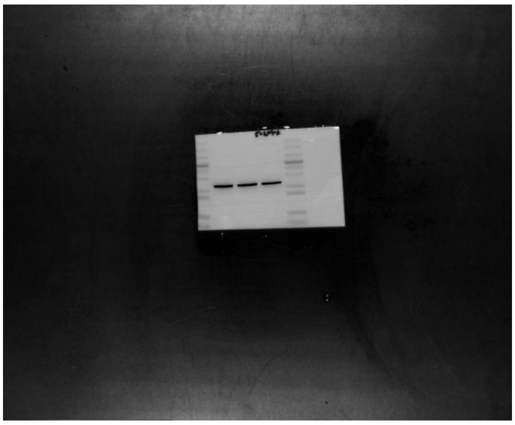

GAPDH

Figure S3B

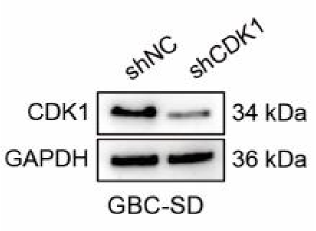

GBC-SD

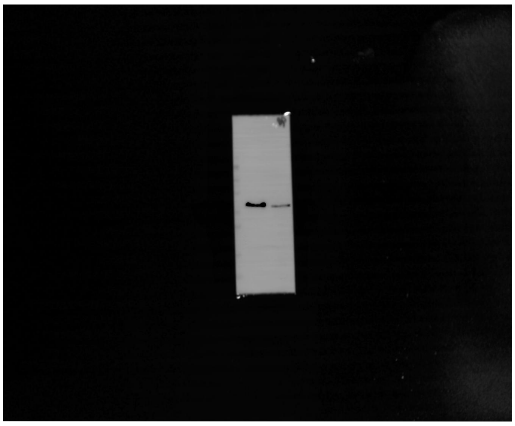

CDK1

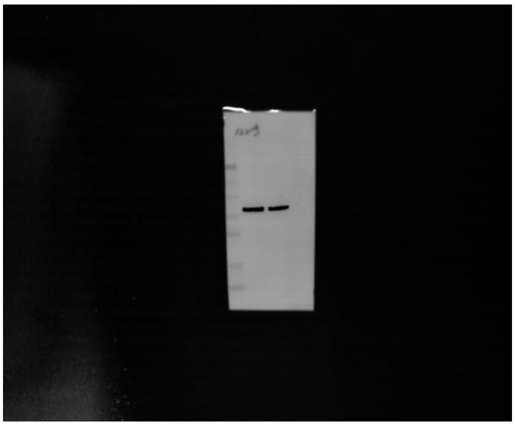

GAPDH

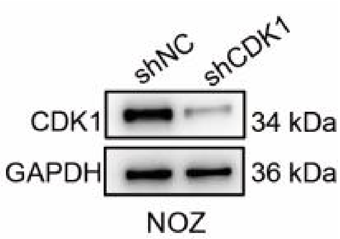

NOZ

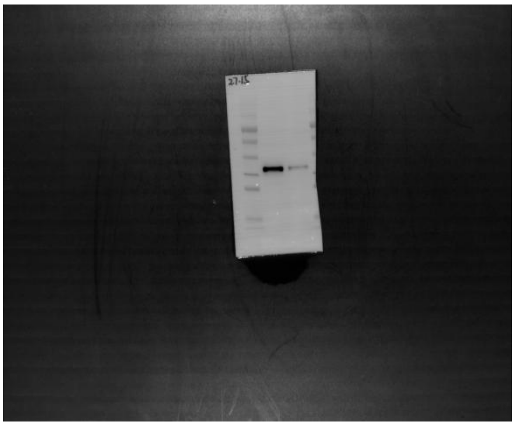

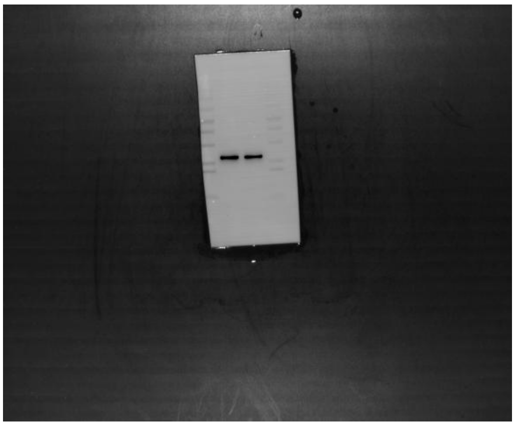

CDK1 GAPDH

Figure S4

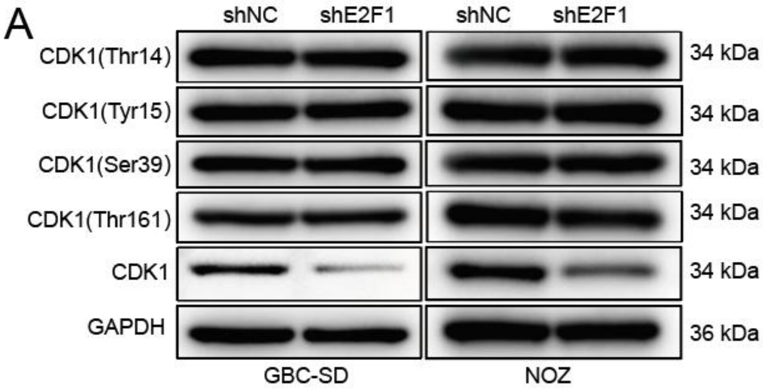

GBC-SD

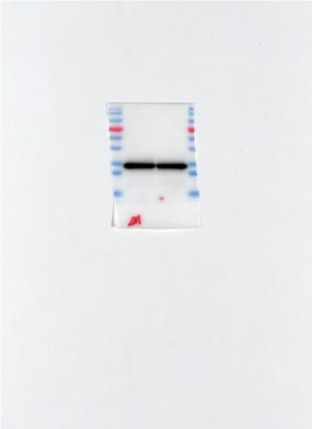

CDK1(Thr14)

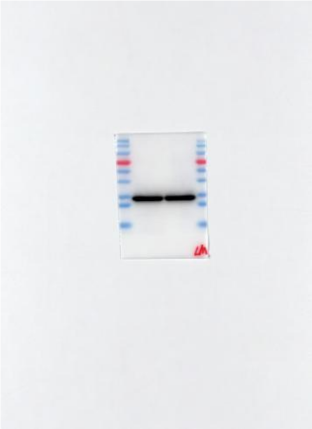

CDK1(Tyr15)

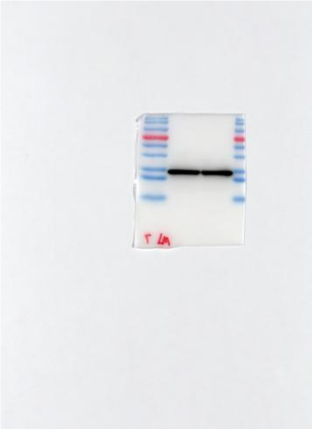

CDK1(Ser39)

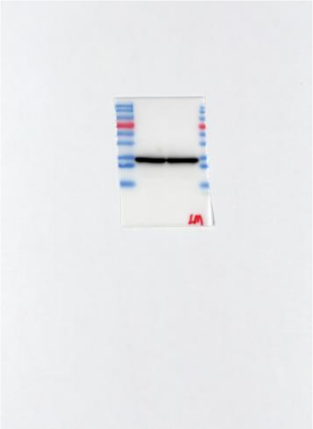

CDK1(Thr161)

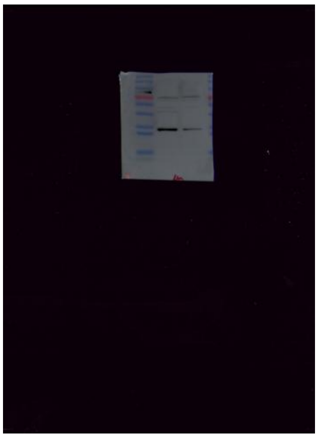

CDK1

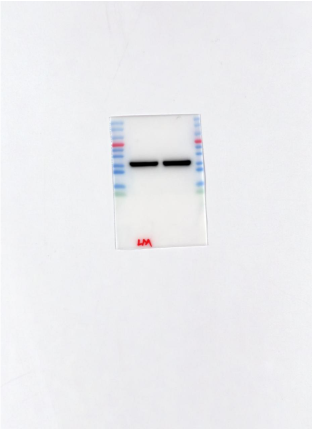

GAPDH

NOZ

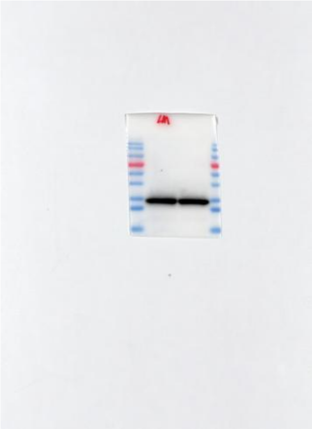

CDK1(Thr14)

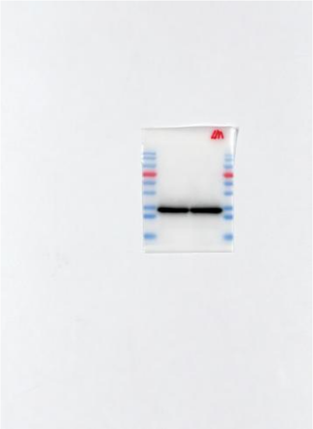

CDK1(Tyr15)

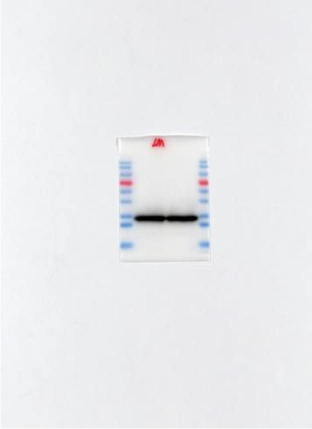

CDK1(Ser39)

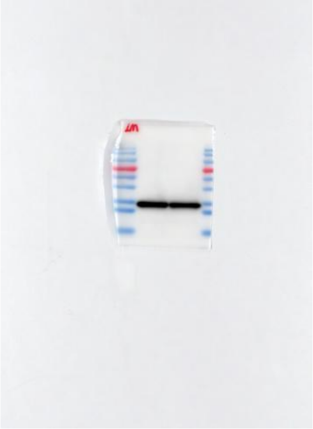

CDK1(Thr161)

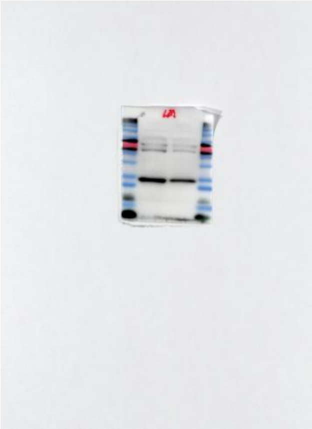

CDK1

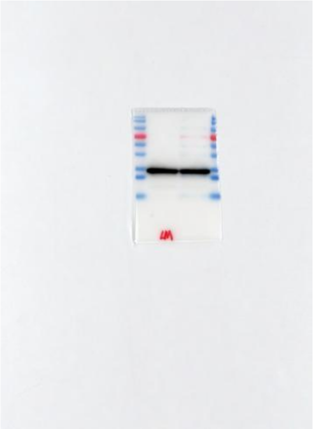

GAPDH

Figure S4

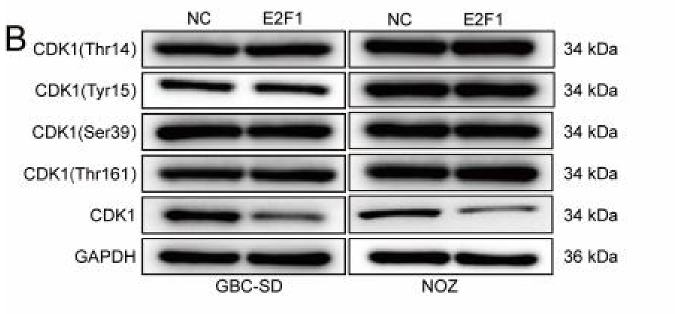

GBC-SD

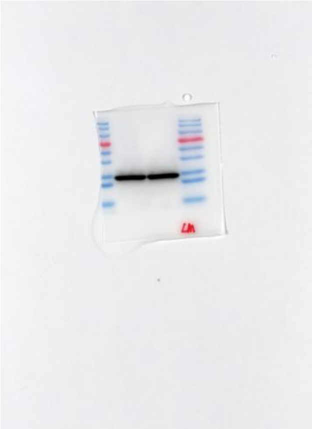

CDK1(Thr14)

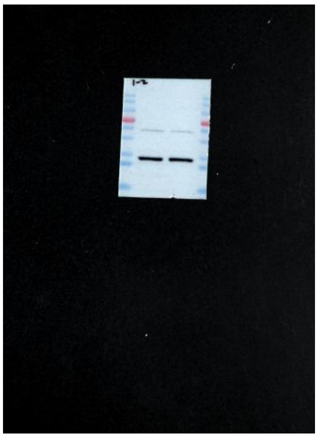

CDK1(Tyr15)

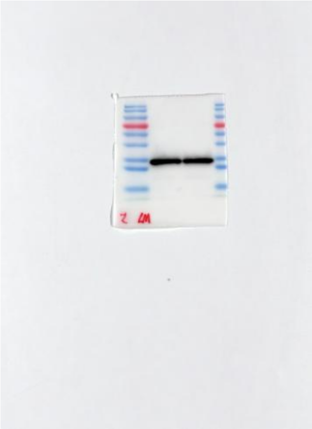

CDK1(Ser39)

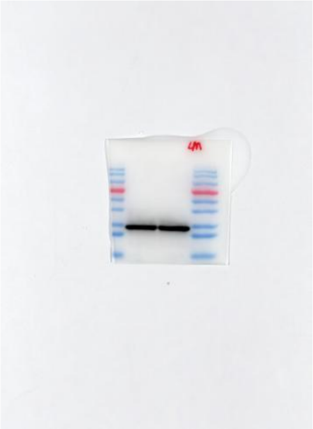

CDK1(Thr161)

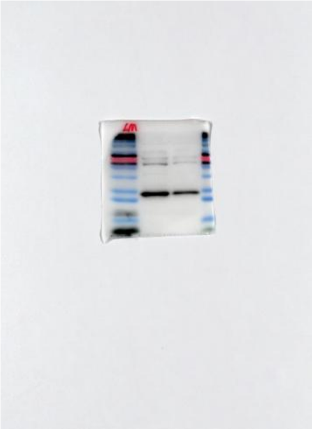

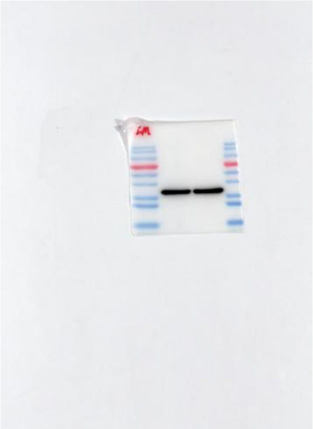

CDK1

GAPDH

NOZ

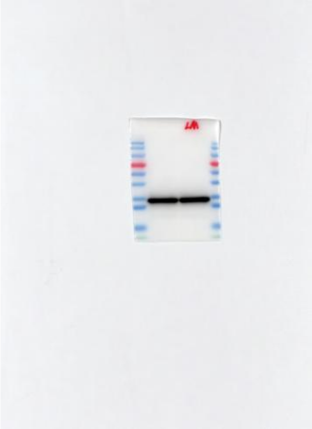

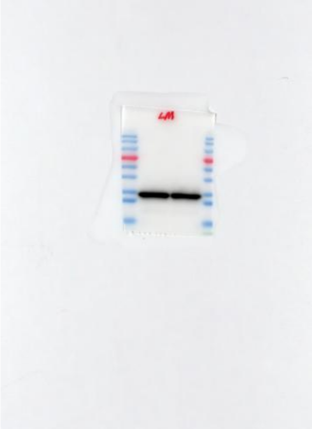

CDK1(Thr14)

CDK1(Tyr15)

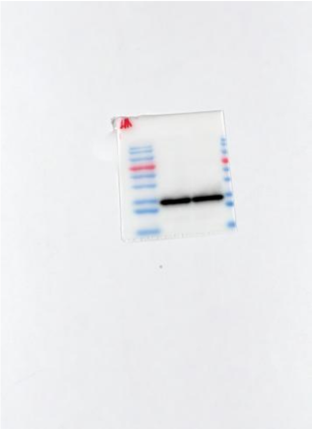

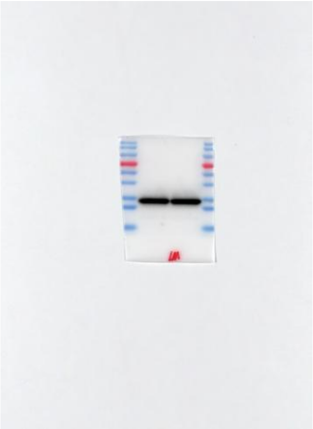

CDK1(Ser39)

CDK1(Thr161)

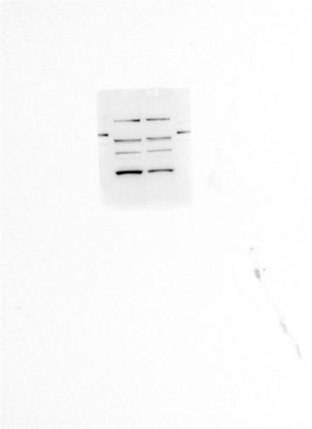

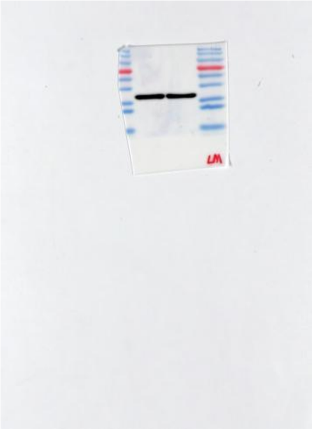

CDK1

GAPDH
