# Supplementary material for: Oncogenic GPRIN1 sustains proliferation and mitochondrial homeostasis via dual‑layer CDK1-PI3K/Akt signalling in gallbladder cancer
Source: Cell Death Dis. 2026 Mar 21;17(1):333. doi: 10.1038/s41419-026-08550-2 (PMC13039753; doi:10.1038/s41419-026-08550-2)
Supplement: Supplementary file 2 — Supplement Table [file 41419_2026_8550_MOESM2_ESM.docx]

**Supplementary Table 1** **ShRNA Sequences**

| Gene | Sequence |
| --- | --- |
| sh-GPRIN1#1 | 5'-GAGTCTTCAGGAAAGACAAAC-3' |
| sh-GPRIN1#2 | 5'-CCATGTTCTTAGAGAAGATGG-3' |
| sh-CDK1 | 5'-GGATGTGCTTATGCAGGATTC-3' |
| shE2F1 | 5'-CGCTATGAGACCTCACTGAAT-3' |
| shNC | 5'-TTCTCCGAACGTGTCACGT-3' |

**Supplementary Table 2** **qRT-PCR primer**

| Gene | Sequence |
| --- | --- |
| GPRIN1 | F: 5'-GAAGGCAGATCCCATGTTTA-3'  R: 5'-GACACTGTATACGTCTTGCT-3' |
| CDK1 | F: 5'-CATTTGGAGTATAGGCACCA-3'  R: 5'-CTGGCCACACTTCATTATTG-3' |
| GAPDH | F: 5'-GGAGCGAGATCCCTCCAAAAT-3'  R: 5'-GGCTGTTGTCATACTTCTCATGG-3' |

**Supplementary Table 3** **Antibodies**

| -Anti- | Brand | Art.No.(article number) | dilution factor |
| --- | --- | --- | --- |
| GPRIN1 | Proteintech | 13771-1-AP | 1:700 |
| GAPDH | Proteintech | 60004-1-Ig | 1:10000 |
| β-Actin | Cell Signaling Technology | 4967 | 1:1000 |
| CDC25C | Proteintech | 16485-1-AP | 1:8000 |
| p-CDC25C | Cell Signaling Technology | 9528 | 1:1000 |
| CyclinB1 | Proteintech | 55004-1-AP | 1:2000 |
| Ki67 | Proteintech | 27309-1-AP | 1:8000 |
| CDK1 | Proteintech | 10762-1-AP | 1:2000 |
| p-CDK1(Thr14) | Bio-Techne | NBP3-13300 | 1:700 |
| p-CDK1(Tyr15) | Invitrogen | PA1-4617 | 1:1000 |
| p-CDK1(Thr161) | Cell Signaling Technology | 9114 | 1:1000 |
| Myt1 | Proteintech | 26204-1-AP | 1:500 |
| E2F1 | Proteintech | 66515-1-Ig | 1:2000 |
| His-Tag | Proteintech | HRP-66005 | 1:10000 |
| GFP-Tag | Proteintech | 66002-1-Ig | 1:15000 |
| PINK1 | Cell Signaling Technology | 6946 | 1:1000 |
| PARKIN | Proteintech | 14060-1-AP | 1:2000 |
| p62/SQSTM1 | Proteintech | 66184-1-Ig | 1:5000 |
| STOML2 | Proteintech | 10348-1-AP | 1:4000 |
| VDAC1 | Cell Signaling Technology | 4866 | 1:1000 |
| p-PI3K | Cell Signaling Technology | 4228 | 1:1000 |
| PI3K | Cell Signaling Technology | 4249 | 1:1000 |
| p-Akt (Ser473) | Cell Signaling Technology | 4060 | 1:2000 |
| Akt | Cell Signaling Technology | 9272 | 1:1000 |

**Supplementary Table 4: CDK1-related phosphopeptides identified in the phosphoproteomic dataset**

| \| **Protein** \| \| --- \| | **Peptide sequence** | **Assigned site (CDK1)** | **Modification** | **log2(KD/NC)** | **P value** |
| --- | --- | --- | --- | --- | --- | --- |
| CDK1 | IRLESEEEGVPST | Ser39 | Phospho (S39) | 0.89 | Not significant |
| CDK1 / CDK2 | IGEGTYGVVYK | Thr14 / Tyr15 region* | Phospho (Y/T), ambiguous | 1.38 | 2.20136E-05 |

*This phosphopeptide corresponds to the N-terminal region of CDK1/CDK2 containing the inhibitory residues Thr14 and Tyr15. Due to sequence identity between CDK1 and CDK2, and limited site localization, the exact residue and kinase cannot be unambiguously assigned.
