# Supplementary material for: Oncogenic GPRIN1 sustains proliferation and mitochondrial homeostasis via dual‑layer CDK1-PI3K/Akt signalling in gallbladder cancer
Source: Cell Death Dis. 2026 Mar 21;17(1):333. doi: 10.1038/s41419-026-08550-2 (PMC13039753; doi:10.1038/s41419-026-08550-2)
Supplement: Supplementary file 3 — Supplementary Figure legends [file 41419_2026_8550_MOESM3_ESM.docx]

**Supplementary figure legends**

**Figure S1. GPRIN1 overexpression protects mitochondrial homeostasis in GBC cells.**
(A, B) Efficient GPRIN1 knockdown (A) and overexpression (B) in GBC-SD and NOZ cells, confirmed by qRT-PCR and Western blot.(C, D) Overexpression of GPRIN1 increased ATP (C) and decreased ROS levels (D).(E, F) GPRIN1 overexpression preserved mitochondrial membrane potential (E, JC-1 assay) and active mitochondrial mass (F, MitoTracker Red staining) upon CCCP challenge. Red JC-1 aggregates indicate high membrane potential.(G) Representative TEM images show attenuated CCCP-induced mitophagy (red arrows) in GPRIN1-overexpressing cells.(H) Levels of non-canonical mitophagy receptors were unaffected by GPRIN1 knockdown.Scale bars are indicated. Data are presented as mean ± SD (n=3). **P < 0.01, ***P < 0.001.

**Figure S2. Multi-step proteomic workflow identifying the GPRIN1–CDK1 signaling axis in gallbladder cancer.**(A, B) Volcano plots showing global proteomic (A) and phosphoproteomic (B) alterations in GBC cells following GPRIN1 knockdown. Red and green dots indicate significantly upregulated and downregulated proteins or phosphosites (P < 0.05; fold change ≥ 1.2), respectively; grey dots indicate non-significant changes.(C) Gene Ontology enrichment analysis of proteins with downregulated phosphosites highlights processes related to cell-cycle progression, including mitotic nuclear division and microtubule organization.(D) Motif-X analysis of downregulated phosphopeptides reveals a strong enrichment of the CDK-associated proline-directed motif (.R….SP…..), suggesting reduced activity of CDK family kinases.(E) Kinase–substrate network analysis further identifies CDK1 (pink square) as a central hub kinase whose predicted activity correlates with reduced phosphorylation of established CDK1 substrates, including Lamin B receptor (LBR) and MAP4, supporting a GPRIN1–CDK1 regulatory relationship.

**Figure S3. Validation of CDK1 knockdown and effect of GPRIN1 on CDK1 mRNA expression.**(A, B) Validation of CDK1 knockdown efficiency in GBC-SD and NOZ cells. Relative CDK1 mRNA levels were measured by qRT-PCR (A), and protein levels were assessed by Western blot (B).(C, D) GPRIN1 regulates CDK1 mRNA expression in GBC cells. (C) qRT-PCR analysis showing that knockdown of GPRIN1 with two independent shRNAs reduces CDK1 mRNA levels. (D) qRT-PCR analysis showing that overexpression of GPRIN1 increases CDK1 mRNA levels. *P < 0.05, **P < 0.01, ***P < 0.001.

**Figure S4. E2F1 modulation alone does not significantly alter CDK1 phosphorylation or cellular free phosphate.** (A, B) Western blots for CDK1 phosphorylation (Thr14, Tyr15, Ser39, Thr161) and total CDK1 in GBC-SD and NOZ cells with (A) E2F1 knockdown (shE2F1 vs. shNC) or (B) E2F1 overexpression (E2F1 vs. NC). (C, D) Intracellular free phosphate levels in GBC-SD and NOZ cells with (C) shE2F1 or (D) E2F1 overexpression.
