# Supplementary figures and images for: Oncogenic GPRIN1 sustains proliferation and mitochondrial homeostasis via dual‑layer CDK1-PI3K/Akt signalling in gallbladder cancer
Source: Cell Death Dis. 2026 Mar 21;17(1):333. doi: 10.1038/s41419-026-08550-2 (PMC13039753; doi:10.1038/s41419-026-08550-2)

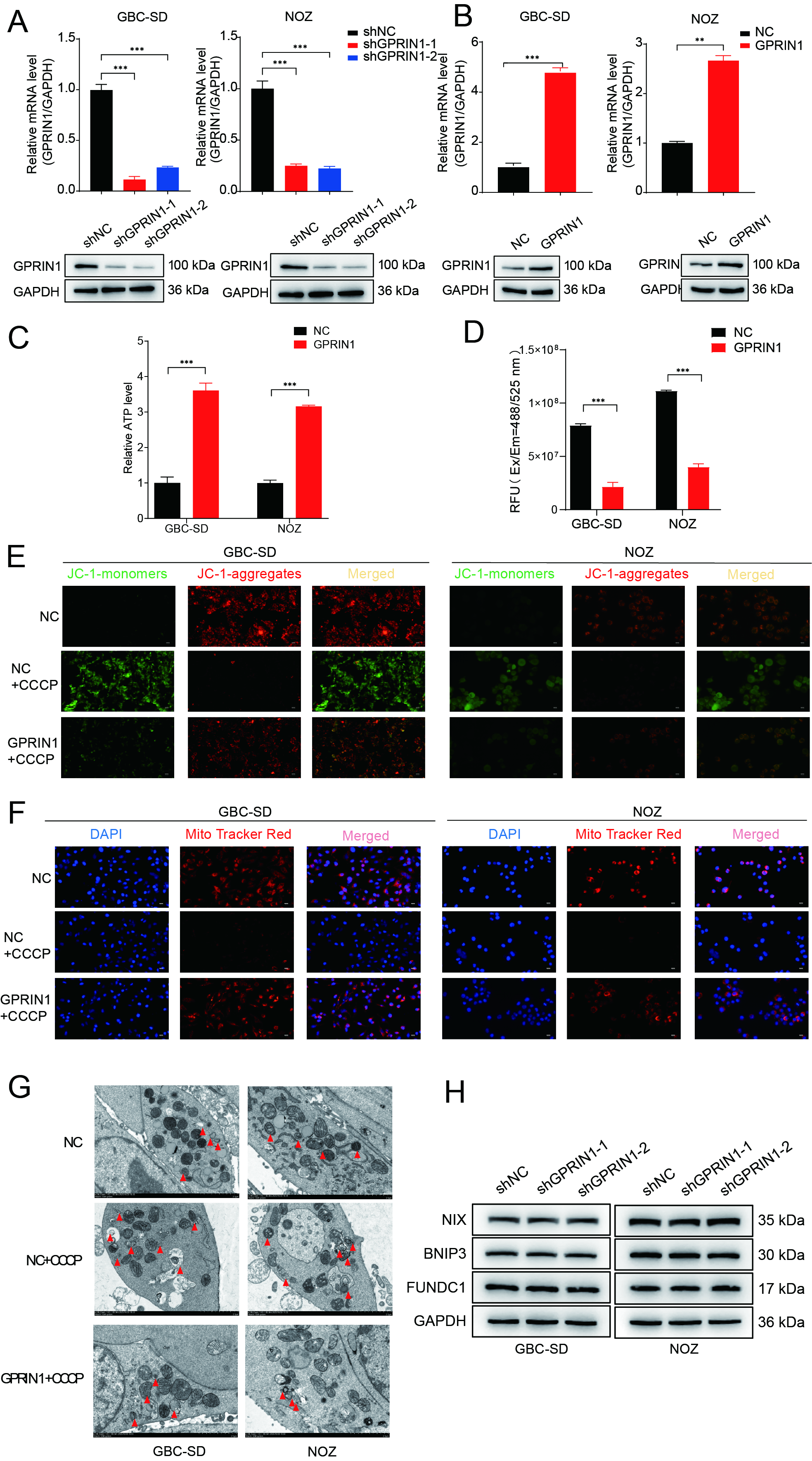

Supplement: Supplementary file 4 — Figure S1 [file 41419_2026_8550_MOESM4_ESM.tif]

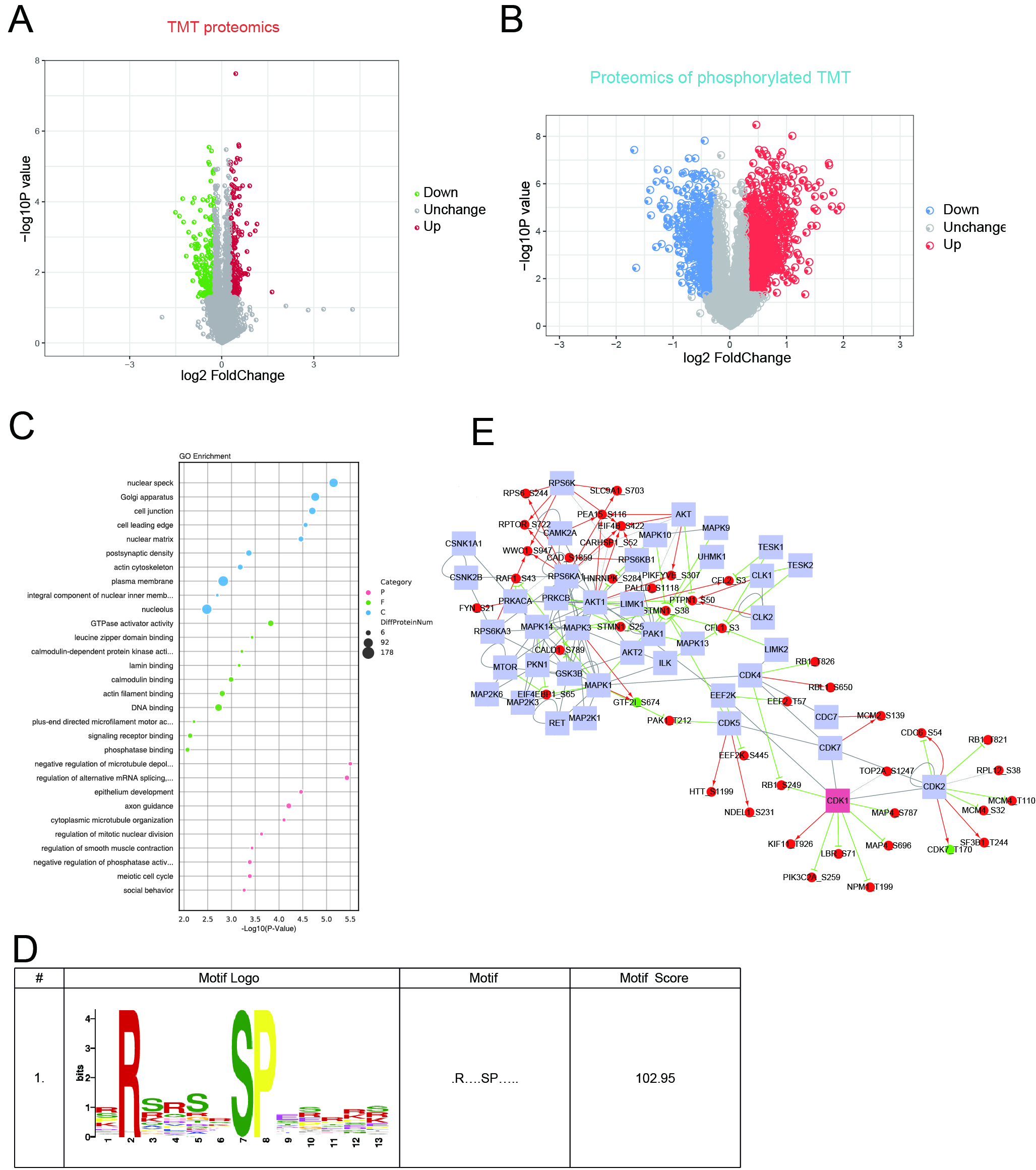

Supplement: Supplementary file 5 — Figure S2 [file 41419_2026_8550_MOESM5_ESM.tif]

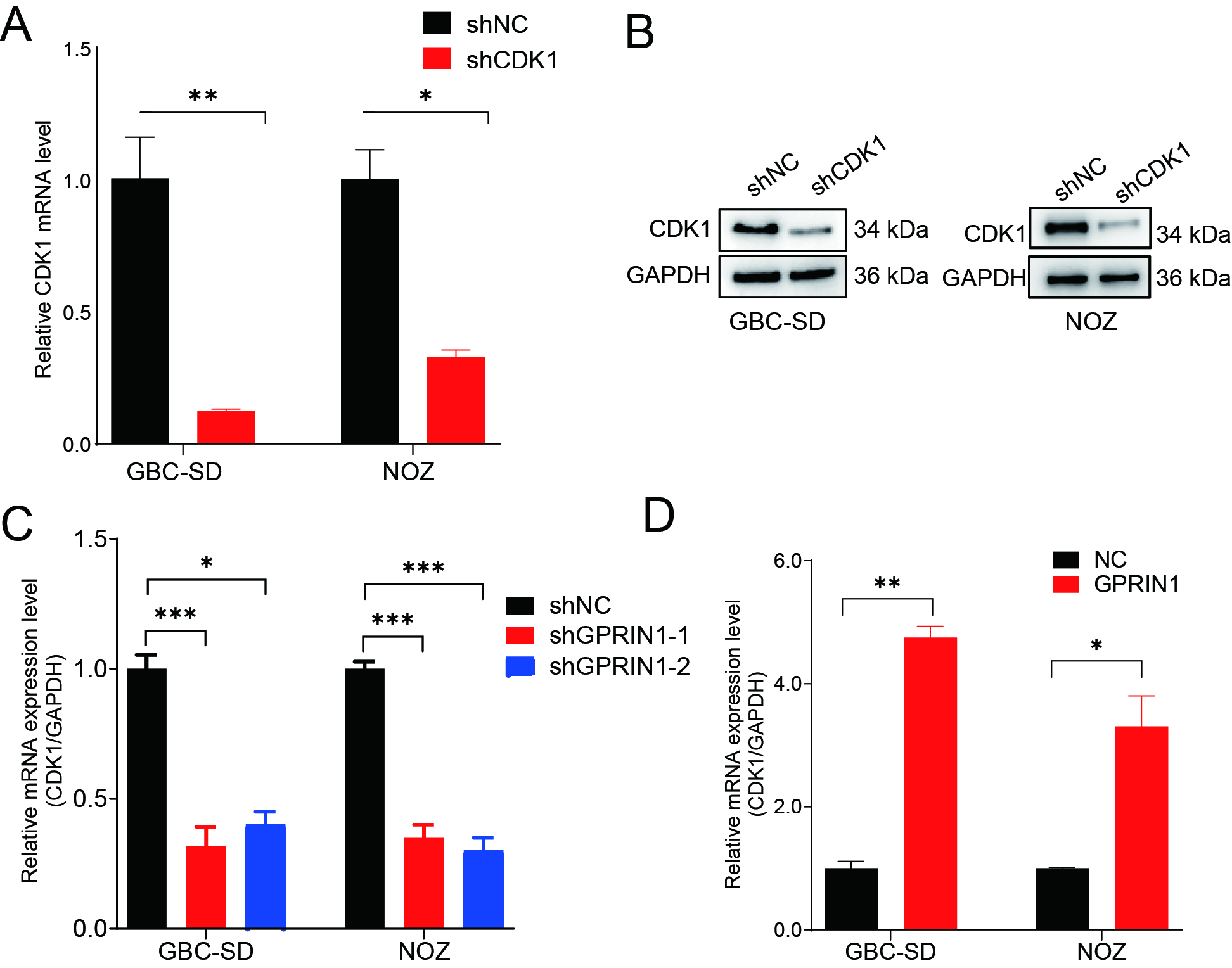

Supplement: Supplementary file 6 — Figure S3 [file 41419_2026_8550_MOESM6_ESM.tif]

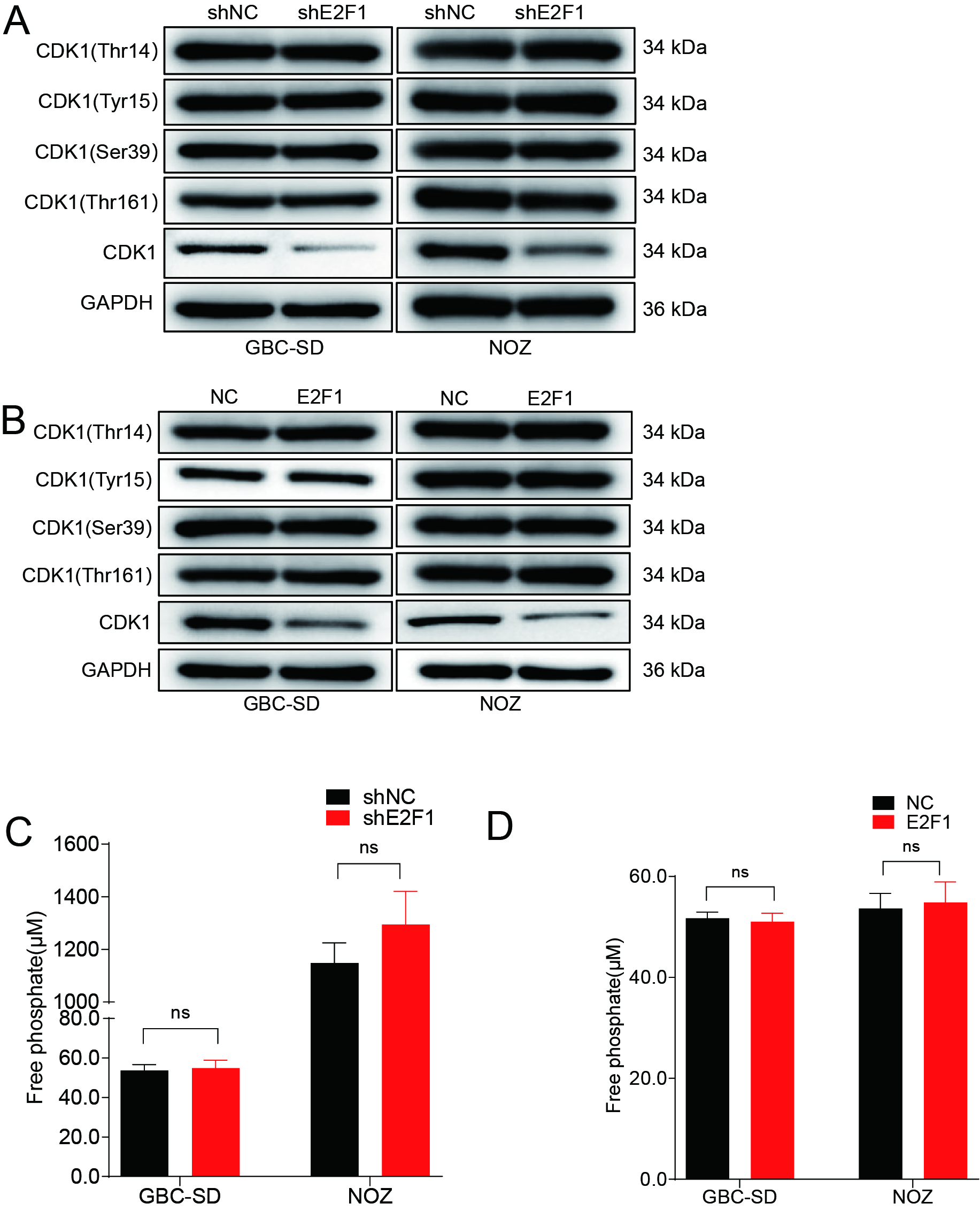

Supplement: Supplementary file 7 — Figure S4 [file 41419_2026_8550_MOESM7_ESM.tif]
